# Incidence rate and associated patient characteristics of liver disease in Wales 2004–2022: a retrospective population-scale observational study

**DOI:** 10.1136/bmjopen-2024-093335

**Published:** 2025-02-10

**Authors:** Jingwei Gao, Ashley Akbari, Haroon Ahmed, Aled Davies, Andrew Yeoman, Thomas Peter Ignatius Pembroke

**Affiliations:** 1 Population Data Science, Faculty of Medicine, Health & Life Science, Swansea University, Swansea, UK; 2 Division of Population Medicine, Cardiff University, Cardiff, UK; 3 PRIME Centre Wales, Cardiff University, Cardiff, UK; 4 Aneurin Bevan University Health Board, Newport, UK; 5 Department of Gastroenterology and Hepatology, University Hospital of Wales, Cardiff, UK

**Keywords:** EPIDEMIOLOGY, Hepatology, PUBLIC HEALTH

## Abstract

**Abstract:**

**Objective:**

To describe the incidence and key demographic, socioeconomic and clinical characteristics of individuals with liver disease in Wales.

**Design and setting:**

This study is designed as a retrospective observational study that linked data of anonymised identified individuals from primary, secondary care and mortality data from the Secure Anonymised Information Linkage (SAIL) Databank in Wales.

**Participants:**

All Welsh residents who registered with a SAIL-contributing general practitioner (GP) and diagnosed with liver disease from 2004 to 2022.

**Primary and secondary outcome measures:**

Our primary outcome is the annual age-standardised incidence rate of liver disease. Secondary outcome is the numbers and frequencies of underlying aetiology and the associated comorbidities.

**Results:**

Between 2004 and 2022, 111 098 individuals received a diagnosis of liver disease in Wales and were included in this study. The incidence of liver disease increased 2.4 folds during the study period (110.3 per 100 000 inhabitants in 2004 to 269.5 per 100 000 inhabitants in 2022). A total of 79 992 individuals (72%) entered the cohort with the underlying aetiology of liver disease, including alcohol-related liver disease, non-alcoholic fatty liver disease (NAFLD), viral hepatitis, metabolic, haemochromatosis and autoimmune liver diseases. NAFLD has contributed to most of the change in incidence.

**Conclusions:**

We observed increasing incidence rates of liver disease in Wales, with NAFLD showing a particularly sharp increase and frequently identified as an underlying condition. A better understanding of the incidence of liver disease is the first step towards effective prevention, early detection and targeted intervention to improve patient outcomes.

**STRENGTHS AND LIMITATIONS OF THIS STUDY**

- The national-scale population-based cohort and long follow-up maximised the generalisability of our finding.
- By incorporating both inpatient and outpatient data, the study not only expanded the sample size but also enabled a thorough analysis of liver disease incidence, enhancing understanding of its clinical implications.
- With the usage of the Secure Anonymised Information Linkage Databank as the research-ready data asset and the incorporation of reproducible research pipelines, this study strengthened its reliability and reproducibility.
- Sensitive Read codes for conditions like hepatitis B virus and hepatitis C virus could not be included due to regulatory laws, impacting the completeness and precision of the data analysis.
- The study only included individuals with a history of general practitioner registration and residency information in Wales, likely overlooking a subpopulation at a significant risk for liver disease.

## Background

 Liver disease is a significant global public health issue and a major contributor to morbidity and mortality.^1^ Globally, cirrhosis and hepatocellular carcinoma account for an estimated 2 million deaths every year.^2^ In the UK, liver disease has become the third most common cause of premature death,^3^ despite mortality rates for other major non-communicable diseases declining.^4^

The management of chronic liver disease frequently involves lifestyle modification, including weight loss and reduced alcohol use, with the goal of reversing factors that can lead to disease progression.^5^ As a result, public health policies in the UK have focused on prevention and early detection, including the UK Government’s Prevention Green Paper to promote disease prevention,^6^ the National Institute for Health and Care Excellence guidance that focused on tackling obesity^7^ and a series of policies to reduce alcohol-related harm.^8^ In November 2022, the Welsh Government published a Quality Statement for Liver Disease,^9^ underlying the importance of the awareness of risk factors and early detection of liver disease and set out its plans to promote the delivery of better quality, higher value and more accessible services for individuals with liver disease.

To effectively improve liver disease management, clinicians, researchers and policy makers must be aware of the epidemiology and clinical profile of individuals with liver disease. However, a significant gap remains in the integration of primary, secondary and mortality data, particularly across the different liver disease stages and aetiologies. To date, much of the epidemiological data is based on the International Classification of Diseases version 10 (ICD-10) coding, which is largely derived from secondary care data sources and therefore likely to underestimate the real-world incidence and prevalence of liver disease.^10 11^ This limitation currently precludes adequate prioritisation of research, targeting of interventions and recruitment of individuals to clinical trials. Understanding the clinical and sociodemographic features associated with liver disease in a large-scale population is essential for the improvement of disease prevention and treatment. Therefore, we aim to fill this gap by integrating primary, secondary and mortality data, providing a more comprehensive and accurate depiction of liver disease across different stages and aetiologies.

The objective of our study is to describe the incidence of liver disease, as well as the key demographic and socioeconomic characteristics and the associated comorbidity of liver disease patients in Wales, as a first step towards improving capacity and capability for liver disease research.

### Materials and method

### Setting and data source

This study is designed as a retrospective cohort study. We used data from the Secure Anonymised Information Linkage (SAIL) Databank, which contains anonymised, individual-level linked electronic health record data for the Welsh population.^12^,^15^ The SAIL Databank includes complete secondary care data and primary care data covering approximately 86% of the Welsh population. These data reflect the demographic diversity of Wales across age, sex and levels of deprivation^16^ and can be generalisable to the broader UK population due to demographic similarities.^17^ SAIL employs a split-file anonymisation process using National Health Service number, name, sex, date of birth and postcode, ensuring confidentiality while enabling the linkage of individual-level data sources.^12 13 16^

To provide a comprehensive overview of liver disease epidemiology, we combined linked primary care, hospital admissions and mortality data. Primary care data were accessed from the Welsh Longitudinal General Practice (WLGP) data, which currently uses the Read Codes V.2 clinical coding system and collects event histories for people registered with a SAIL-supplying general practice (GP) in Wales. Hospital admission data, including inpatient admissions (emergency, elective and maternity) and daycare procedures, were collected from the Patient Episode Database for Wales (PEDW). Mortality data came from the Annual District Death Extract (ADDE) by the Office for National Statistics (ONS) death and contains the cause of death and contributory comorbidities. Both hospital admission and mortality data were coded using the ICD-10 system. We derived demographic and deprivation data from the Welsh Demographic Service Dataset and used the Welsh Index of Multiple Deprivation (WIMD) 2019 quintiles to measure relative area-level deprivation based on geographical residential location from the 2011 Lower-layer Super Output Areas (LSOAs).

### Study population

We linked data from all individuals in the WLGP, PEDW and ADDE data within the SAIL Databank uses a unique anonymised individual identifier known as an Anonymised Linkage Field (ALF). Individuals were extracted based on ALF and filtered for good data linkage status based on existing methodology.^12 13^ We excluded individuals not registered with a SAIL-contributing GP or lacking residency information and identified those who received a liver disease diagnosis from 1 January 2004 to 31 December 2022. The first liver disease diagnosis across all data sources is considered to be the index liver disease event. Individuals were required to be residents of Wales at the time of cohort entry. GP registration was required if the index event was from WLGP. All individuals were followed until the earliest of GP deregistration (applicable only to individuals whose index event was identified from WLGP), moving out of Wales, death or the end of the study period (31 December 2022). GP deregistration is defined as the termination of an individual’s registration with a SAIL-contributing GP, as indicated by the last end date in the registration records. We divided the full cohort into four distinct groups: individuals identified in WLGP were assigned to the primary care group; those identified in PEDW were assigned to the secondary care group; individuals identified in ADDE were assigned to the mortality group and individuals identified across multiple data sources (two or more) were assigned to a multisource group.

## Measurements

### Definition of stages of liver disease and time of cohort entry

We applied a hierarchy of three tiers of potential aetiological diagnoses and five discrete stages of chronic liver disease based on perceived clinical importance and natural history of liver disease progression as was described in our previous study.^18^ The first stage is the underlying aetiological conditions without the presentation of cirrhosis and was divided into three tiers. Tier 1 aetiologies of liver disease include alcohol-related liver disease (ArLD), non-alcoholic fatty liver disease (NAFLD), metabolic liver disease, hepatitis B virus (HBV), hepatitis C virus (HCV), autoimmune liver disease, haemochromatosis; tier 2 aetiologies include unspecified hepatitis, congestive hepatopathy and toxic liver disease and tier 3 aetiologies were other miscellaneous diagnoses. As the disease progresses, various stages of liver disease are defined as follows: hepatic fibrosis and cirrhosis were categorised as stage 2, portal hypertension as stage 3, hepatic decompensation as stage 4 and hepatocellular carcinoma (HCC), intrahepatic cholangiocarcinoma (ICC) and other primary liver cancers were classified under stage 5. Acute liver diseases were analysed separately from chronic liver diseases as they carry different challenges for primary and secondary care. Acute liver diseases were defined as conditions including acute viral hepatitis, Budd-Chiari syndrome, acute liver failure, infections and infarctions and other unspecified acute liver injuries. A list of ICD-10 and Read V.2 codes to identify individuals with liver disease can be found in the online supplemental table 1.

We defined the time of the first chronic or acute liver disease diagnosis across all data sources as the index date. To assess the proportion of individuals with liver disease who presented late, the diagnoses were sequenced according to the natural history of the disease: aetiological diagnoses (stage 1), followed by cirrhosis, portal hypertension, decompensation and then HCC formation^18^ (online supplemental figure 1).

### Comorbidities and drug prescription history

We collected data on demographic characteristics (age, sex and WIMD 2019 quintile), with age divided by birth year in 10-year intervals, except for the 0–17 age group and 18–29 age group. Comorbidities including cardiovascular disease (CVD)-related conditions (heart failure, transient ischaemic attack, other ischaemic disease, atrial fibrillation, peripheral vascular disease, angina, and stroke), diabetes, hypertension and antihypertensive usage were tracked up to 10 years prior to cohort entry. Lists of ICD-10 and Read V.2 codes to identify the associated conditions are available as supplemental tables (online supplemental tables 2-1 and 2-2).

### Incidence of liver disease

The incidence rate was calculated as the number of incident cases per Welsh residents. An incident case was defined as an individual having a first liver-related diagnosis during the study period (1 January 2004–31 December 2022) with no prior liver disease history from 1 January 1994 to 31 December 2003. To determine the most clinically significant aetiology, we only considered the most advanced stage of diagnosis on the incident event date. The Welsh population data were obtained from the ONS population estimates.^19^ The incidence rate was directly standardised using the European Union standard population 2013 and were reported per 100 000 inhabitants in each calendar year from 2004 to 2022, with corresponding 95% CIs. Analysis was repeated for different data sources, liver disease aetiologies and stages.

Study findings were reported in accordance with the applicable reporting guidelines for observational studies using administrative data (Strengthening the Reporting of Observational Studies in Epidemiology^20^ and REporting of studies Conducted using Observational Routinely-collected Data.^21^ Data cleaning, cohort assembly and statistical analyses were performed using Structured Query Language (IBM Db2 V.11.1) and R (V.4.1.0–V.4.1.3) within the SAIL Databank privacy-protecting trusted research environment.

### Patient and public involvement

The Liver Research Cymru Patient Advisory group was established to provide patient and public involvement (PPI) input into the development of research in Wales. The research questions and methodology addressed in this body of work were developed following the discussion of their priorities based on their lived experience of liver disease. The results were shared with this group, and their feedback was sought in the interpretation and presentation of these data.

## Results

### Demographic and socioeconomic characteristics

**Figure 1:**
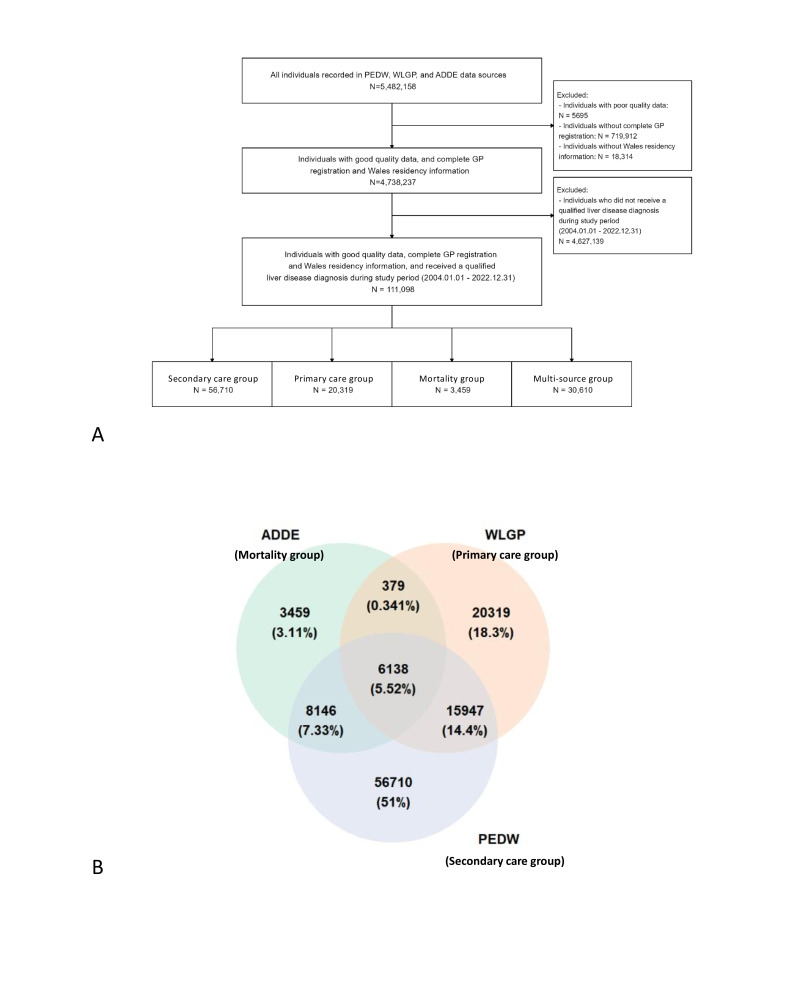
Flowchart of study population selection **(A)** and Venn diagram of study cohort composition **(B)** ADDE, Annual District Death Extract; PEDW, Patient Episode Database for Wales; WLGP, Welsh Longitudinal General Practice.

We identified a total of 111 098 individuals with liver disease from all three data sources from 1 January 2004 to 31 December 2022, contributing 441 885 person-years of follow-up (figure 1A). Of the eligible individuals, 57 491 (51.7 %) were male. The median (IQR) age was 59 (47–72). Approximately 50% of the individuals (55 787) entered the cohort after 2016, and 27 178 (24.5 %) individuals came from the most deprived areas. Of the 111 098 individuals, 20 319 (18.3%) individuals could be tracked from WLGP (primary care data source), forming the primary care group, 56 710 (51.0 %) from PEDW (secondary data source) constituted the secondary care group and 3459 (3.1 %) from ADDE (mortality data source) formed the mortality group. Additionally, 30 610 (27.5%) appeared in multiple sources, forming the multi-source group (figure 1B). The demographic variation is notable with a higher percentage of male and elderly individuals, and a higher degree of socioeconomic deprivation in the mortality group (table 1).

**Table 1:** Demographic and socioeconomic characteristics of liver disease patients

Demographic characteristics	Full cohort, n=111 098*	Primary care group, n=20 319*	Secondary care group, n=56 710*	Mortality group, n=3459*	Multisource group, n=30 610*
Sex					
Male	57 491 (51.7%)	10 478 (51.6%)	27 558 (48.6%)	2019 (58.4%)	17 436 (57.0%)
Female	53 607 (48.3%)	9841 (48.4%)	29 152 (51.4%)	1440 (41.6%)	13 174 (43.0%)
Age					
0–17	1445 (1.3%)	342 (1.7%)	814 (1.4%)	<10 (<0.3%)	<290 (<1.0%)
18–29	5862 (5.3%)	1447 (7.1%)	3486 (6.1%)	<40 (<1.2%)	<900 (<0%)
30–39	10 109 (9.1%)	2520 (12.4%)	5099 (9.0%)	143 (4.1%)	2347 (7.7%)
40–49	15 370 (13.8%)	3744 (18.4%)	6316 (11.1%)	421 (12.2%)	4889 (16.0%)
50–59	22 799 (20.5%)	5356 (26.4%)	9332 (16.5%)	705 (20.4%)	7406 (24.2%)
60–69	23 264 (20.9%)	4271 (21.0%)	11 026 (19.4%)	758 (21.9%)	7209 (23.6%)
70–79	19 189 (17.3%)	2117 (10.4%)	11 335 (20.0%)	693 (20.0%)	5044 (16.5%)
80+	13 060 (11.8%)	522 (2.6%)	9302 (16.4%)	702 (20.3%)	2534 (8.3%)
Cohort entry year					
2004–2007	17 099 (15.4%)	1949 (9.6%)	7345 (13.0%)	617 (17.8%)	7188 (23.5%)
2008–2011	17 554 (15.8%)	2513 (12.4%)	8484 (15.0%)	705 (20.4%)	5852 (19.1%)
2012–2015	20 658 (18.6%)	3109 (15.3%)	10 682 (18.8%)	731 (21.1%)	6136 (20.0%)
2016–2019	30 448 (27.4%)	6683 (32.9%)	15 521 (27.4%)	749 (21.7%)	7495 (24.5%)
2020–2022	25 339 (22.8%)	6065 (29.8%)	14 678 (25.9%)	657 (19.0%)	3939 (12.9%)
WIMD 2019 quintiles					
1, most deprived	27 178 (24.5%)	4417 (21.7%)	14 012 (24.7%)	928 (26.8%)	7821 (25.6%)
2	24 391 (22.0%)	4203 (20.7%)	12 411 (21.9%)	858 (24.8%)	6919 (22.6%)
3	21 066 (19.0%)	3828 (18.8%)	10 802 (19.0%)	647 (18.7%)	5789 (18.9%)
4	19 619 (17.7%)	3949 (19.4%)	9855 (17.4%)	545 (15.8%)	5270 (17.2%)
5, least deprived	18 844 (17.0%)	3922 (19.3%)	9630 (17.0%)	481 (13.9%)	4811 (15.7%)

* n (%).

WIMD, Welsh Index of Multiple Deprivation.

### Incidence of chronic liver disease

**Figure 2:**
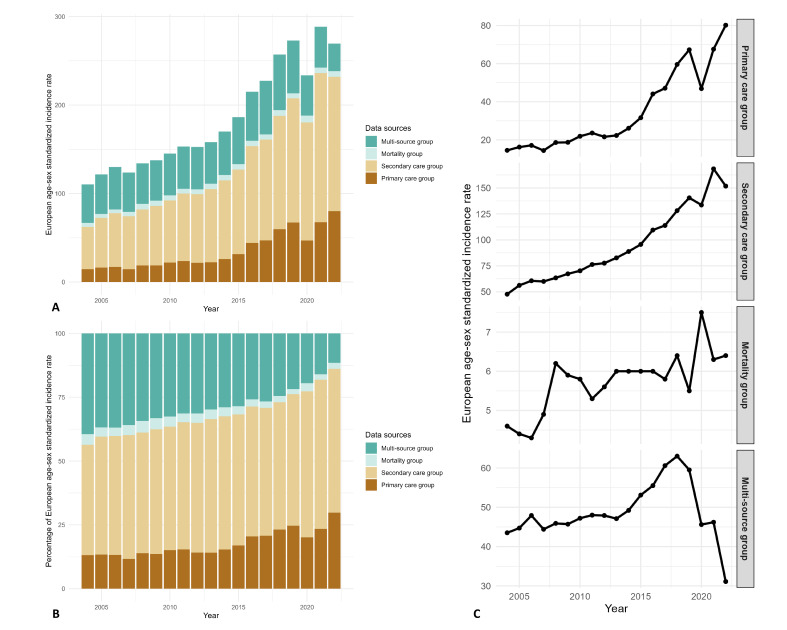
Changes in standardised incidence per 100 000 inhabitants by data source (2004–2022). Bar chart presenting the changes in annual European age-sex standardised incidence rate and percentages by data sources. The x-axis represents time in years, and the y-axis represents the incidence rate in **(A), **percentages in **(B). **Line chart presenting the trends in annual European age-sex standardised incidence rate. The x-axis represents time in years, and the y-axis represents incidence rate in **(C)**.

We observed an increasing trend of liver disease from 2004 to 2022 in the Welsh population. The age-sex standardised incidence rate increased by 2.4 times during the 18 years of follow-up (110.3 per 100 000 inhabitants in 2004; 269.5 per 100 000 inhabitants in 2022, table 2). This was significantly contributed by a 5.5 times increase in the incidence rate of the primary care group, followed by 3.2 times increase in the secondary care group. The proportion of incident cases in primary care and secondary care groups increased by 14%–16% during the past two decades (figure 2A–C, online supplemental table 3-1).

**Table 2:** Crude and standardised incidence of chronic liver disease from 2004 to 2022

Year	Wales population	Incident cases	Crude incidence (95% CI)	STD incidence (95% CI)
2004	2 957 422	3060	103.5 (99.8 to 107.2)	110.3 (106.3 to 114.3)
2005	2 969 309	3403	114.6 (110.8 to 118.5)	121.5 (117.4 to 125.8)
2006	2 985 668	3670	122.9 (119.0 to 127.0)	129.8 (125.6 to 134.2)
2007	3 006 299	3537	117.7 (113.8 to 121.6)	123.8 (119.7 to 128.0)
2008	3 025 867	3867	127.8 (123.8 to 131.9)	134.2 (129.9 to 138.5)
2009	3 038 872	4008	131.9 (127.8 to 136.0)	137.5 (133.2 to 141.9)
2010	3 049 971	4268	139.9 (135.8 to 144.2)	145.1 (140.7 to 149.6)
2011	3 063 758	4519	147.5 (143.2 to 151.9)	153.2 (148.7 to 157.7)
2012	3 070 928	4546	148.0 (143.8 to 152.4)	152.6 (148.2 to 157.2)
2013	3 071 058	4733	154.1 (149.8 to 158.6)	158.0 (153.5 to 162.6)
2014	3 073 788	5145	167.4 (162.8 to 172.0)	170.1 (165.5 to 174.9)
2015	3 072 739	5659	184.2 (179.4 to 189.0)	186.2 (181.4 to 191.2)
2016	3 077 165	6548	212.8 (207.7 to 218.0)	215.1 (209.9 to 220.4)
2017	3 081 366	6981	226.6 (221.3 to 231.9)	227.4 (222.1 to 232.9)
2018	3 083 840	7953	257.9 (252.3 to 263.6)	257.1 (251.5 to 262.9)
2019	3 087 732	8481	274.7 (268.9 to 280.6)	272.8 (266.9 to 278.7)
2020	3 104 483	7327	236.0 (230.6 to 241.5)	233.6 (228.2 to 239.1)
2021	3 105 633	9136	294.2 (288.2 to 300.3)	288.6 (282.6 to 294.6)
2022	3 131 640	8596	274.5 (268.7 to 280.4)	269.5 (263.8 to 275.3)

STD, standardised.

The incidence of liver disease has risen across all stages, with stage 2 (cirrhosis) and stage 3 diagnoses (portal hypertension) experiencing the most notable increase from 2004 to 2022 (both stage 2 and stage 3 by 2.84 times). However, the increases in the higher-stage liver disease cases were less pronounced, particularly for stage 4 (1.1 times). Consequently, the proportion of more advanced liver disease diagnoses decreased (stage 4 and stage 5: 9.1% in 2004, 4.9% in 2022, decreased by 1.86 times since 2004, figure 3A–C, online supplemental table 3-2).

**Figure 3:**
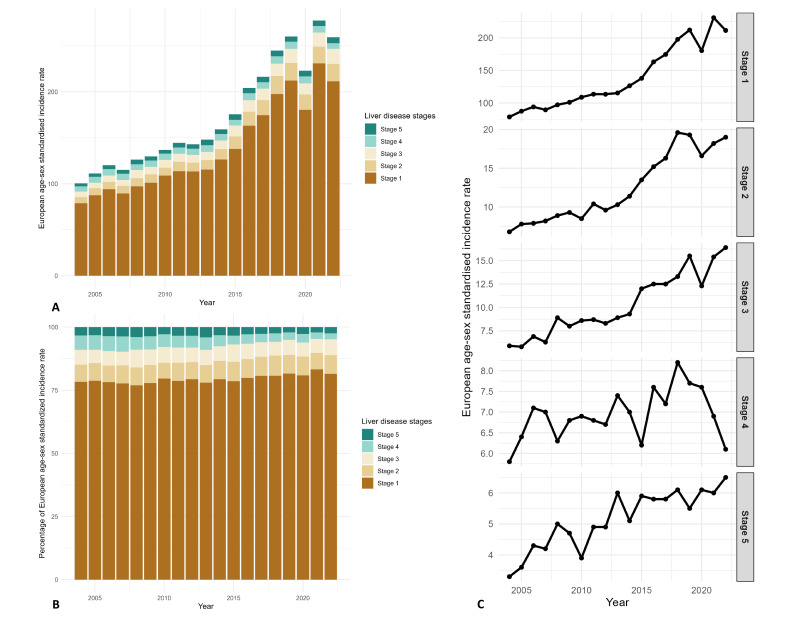
Changes in standardised incidence per 100 000 inhabitants by stages (2004–2022). Bar chart presenting the changes in annual European age-sex standardised incidence rate and percentages by stages. The x-axis represents time in years, and the y-axis represents the incidence rate in **(A),** percentages in **(B).** Line chart presenting the trends in annual European age-sex standardised incidence rate. The x-axis represents time in years, and the y-axis represents the incidence rate in **(C)**.

**Figure 4:**
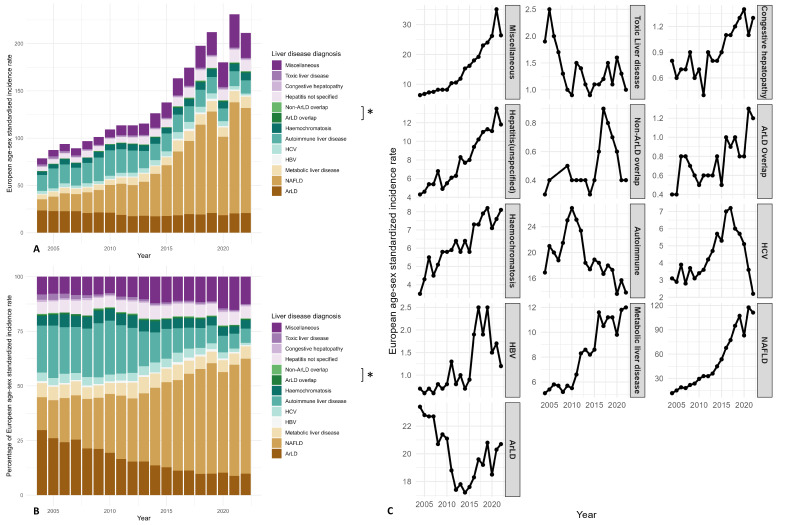
Changes in standardised incidence per 100 000 inhabitants by liver disease aetiologies (2004–2022). Bar chart presenting the changes in annual European age-sex standardised incidence rate and percentages by aetiologies. The x-axis represents time in years, and the y-axis represents the incidence rate in **(A),** percentages in **(B).** Line chart presenting the trends in annual European age-sex standardised incidence rate. The x-axis represents time in years, and the y-axis represents the incidence rate in **(C). **ArLD, alcohol-related liver disease; HBV, hepatitis B virus; HCV, hepatitis C virus; NAFLD, non-alcoholic fatty liver disease. * ArLD overlap: if an individual had two or more diagnoses from liver disease aetiologies (ArLD, NAFLD, HBV, HCV, metabolic, haemochromatosis and autoimmune liver diseases) and one of them was ArLD; non-ArLD overlap: if an individual had two or more diagnoses from liver disease aetiologies but none of them was ArLD.

We further analysed the stage 1 aetiologies by each diagnosis. We observed the most notable increase in the incidence of NAFLD (11.8 per 100 000 inhabitants in 2004, 111.2 per 100 000 inhabitants in 2022, increased 9.4 times since 2004), resulting in a 3.5 time increase in the proportion and ultimately accounting for over half of the cases by the end of our follow-up. Conversely, the proportion of ArLD cases decreased by 67.1%, given that the incidence rate decreased during the past two decades (figure 4A,B, online supplemental table 3-3). A breakdown of NAFLD incidence by data sources demonstrated 12 and 14 times increases in the primary care and the secondary care group, respectively (online supplemental table 4-1). In contrast, the increase in the incidence of ArLD in the primary care group was more than twice that of the secondary care group (online supplemental table 4-2).

We did not observe an increasing trend for HBV and HCV. Both conditions fluctuated during the past two decades, with HBV cases increasing by 3.6 times and HCV cases 2.3 times from 2004 to 2017, followed by a decrease from 2017 to 2022 (HBV by 2.1 times; HCV by 3.3 times). Consequently, the proportion of HBV and HCV cases decreased from 2004 to 2022 (HBV by 1.7 times; HCV by 4.5 times, figure 4A–C, online supplemental table 3-3).

### Underlying liver disease conditions

Of the 111 098 individuals with liver disease, 79 992 (72.0 %) entered the cohort due to a tier 1 aetiology. This was mainly contributed by the high prevalence of NAFLD (33 655 (30.3%)). Most NAFLD cases were identified in the primary care group (13 565 (66.8%)) and the least NAFLD cases in the mortality group (727 (21.0%)). This aligns with the distribution of individuals with tier 1 aetiology, whose proportion was the highest in the primary care group (18 453 (90.8%)) and the lowest in the mortality group (1728 (50.0%)). In contrast, tier 2 and tier 3 diagnoses were higher in the secondary care group. The proportion of advanced liver disease stages was lower in the primary care group (stage 3: 166 (0.8%), stage 4: 53 (0.3%), stage 5: 121 (0.6%)), but higher in the mortality group (stage 4: 276 (8.0%), stage 5: 473 (13.7%)) (table 3).

**Table 3:** The underlying liver disease conditions at cohort entry

Phenotype	Full cohort, n=111 098*	Primary care group, n=20 319*	Secondary care group, n=56 710*	Mortality group, n=3459*	Multi-source group, n=30 610*
**Chronic liver disease**					
Stage 1—tier 1	79 992 (72.0%)	18 453 (90.8%)	35 102 (61.9%)	1728 (50.0%)	24 709 (80.7%)
ArLD	19 760 (17.8%)	2116 (10.4%)	4566 (8.1%)	887 (25.6%)	12 191 (39.8%)
NAFLD	33 655 (30.3%)	13 565 (66.8%)	13 444 (23.7%)	727 (21.0%)	5919 (19.3%)
Metabolic liver disease	5469 (4.9%)	782 (3.8%)	3887 (6.9%)	69 (2.0%)	731 (2.4%)
HBV	1063 (1.0%)	0 (0.0%)	905 (1.6%)	0 (0.0%)	158 (0.5%)
HCV	3539 (3.2%)	0 (0.0%)	2697 (4.8%)	13 (0.4%)	829 (2.7%)
Autoimmune liver disease	13 582 (12.2%)	815 (4.0%)	9060 (16.0%)	199 (5.8%)	3508 (11.5%)
Haemochromatosis	4111 (3.7%)	1209 (6.0%)	<940 (<1.7%)	<10(<0.2%)	1958 (6.4%)
ArLD overlap	799 (0.7%)	15 (0.1%)	158 (0.3%)	173 (5.0%)	453 (1.5%)
Non-ArLD overlap	371 (0.3%)	<20 (<0.1%)	228 (0.4%)	<10 (<0.2%)	123 (0.4%)
Stage 1—tier 2	6111 (5.5%)	81 (0.4%)	4906 (8.7%)	127 (3.7%)	997 (3.3%)
Hepatitis not specified	4783 (4.3%)	11 (0.1%)	3867 (6.8%)	102 (2.9%)	803 (2.6%)
Congestive hepatopathy	574 (0.5%)	<10 (<0.1%)	514 (0.9%)	<10 (<0.2%)	<60 (<0.2%)
Toxic liver disease	757 (0.7%)	68 (0.3%)	526 (0.9%)	21 (0.6%)	142 (0.5%)
Stage 1—tier 3	8426 (7.6%)	36 (0.2%)	8132 (14.3%)	37 (1.1%)	221 (0.7%)
Miscellaneous	8426 (7.6%)	36 (0.2%)	8132 (14.3%)	37 (1.1%)	221 (0.7%)
Stage 2	4562 (4.1%)	506 (2.5%)	1664 (2.9%)	690 (19.9%)	1702 (5.6%)
Hepatic fibrosis	261 (0.2%)	64 (0.3%)	135 (0.2%)	<10 (<0.2%)	<60 (<0.2%)
Cirrhosis	4301 (3.9%)	442 (2.2%)	1529 (2.7%)	682 (19.7%)	1648 (5.4%)
Stage 3	3040 (2.7%)	166 (0.8%)	2171 (3.8%)	43 (1.2%)	660 (2.2%)
Portal hypertension	3040 (2.7%)	166 (0.8%)	2171 (3.8%)	43 (1.2%)	660 (2.2%)
Stage 4	3221 (2.9%)	53 (0.3%)	2517 (4.4%)	276 (8.0%)	375 (1.2%)
Hepatic decompensation	3221 (2.9%)	53 (0.3%)	2517 (4.4%)	276 (8.0%)	375 (1.2%)
Stage 5	2233 (2.0%)	121 (0.6%)	341 (0.6%)	473 (13.7%)	1298 (4.2%)
Hepatocellular carcinoma	748 (0.7%)	71 (0.3%)	83 (0.1%)	86 (2.5%)	508 (1.7%)
Intrahepatic cholangiocarcinoma	1455 (1.3%)	47 (0.2%)	232 (0.4%)	387 (11.2%)	789 (2.6%)
Other primary liver cancer	39 (0.0%)	<10 (<0.1%)	26 (0.0%)	<10 (<0.2%)	<10 (<0.1%)
**Acute liver disease**					
Acute liver disease	3513 (3.2%)	903 (4.4%)	1877 (3.3%)	85 (2.5%)	648 (2.1%)
Budd-Chiari	184 (0.2%)	<10 (<0.1%)	144 (0.3%)	<20 (<0.1%)	20 (0.1%)
Acute liver failure	27 (0.0%)	<30 (<0.2%)	0 (0.0%)	0 (0.0%)	<10 (<0.1%)
Infection/infarction	1343 (1.2%)	62 (0.3%)	757 (1.3%)	67 (1.9%)	457 (1.5%)
Other non-specified acute liver injuries	71 (0.1%)	45 (0.2%)	<20 (<0.1%)	0 (0.0%)	<10 (<0.1%)

* n (%).

ArLD, alcohol-related liver disease; HBV, hepatitis B virus; HCV, hepatitis C virus; NAFLD, non-alcohol fatty liver disease.

### Comorbidities associated with liver disease

Among all eligible individuals with liver disease, 40 427 (36.4%) individuals had onset hypertension or initiated an antihypertensive medication, followed by 8923 (8.0%) individuals who had CVD-related conditions and 7658 (6.9%) had diabetes. We observed the higher proportions of comorbidities in the mortality group (CVD: 11.2%; diabetes: 8.1%; hypertension/antihypertensive: 39.7%) and the lowest in the primary care group (CVD: 4.2%; diabetes: 6.3%; hypertension/antihypertensive: 35.7%) (table 4). The burden of comorbidities was the lowest for individuals with stage 1 liver disease conditions but higher for late-stage (CVD highest for stage 4: 14.2%; hypertension/antihypertensive for stage 5: 45.1%) and stage 2 individuals (diabetes: 13.4%) (online supplemental table 5-1). Among the individuals who entered the cohort with a stage 1 aetiology, individuals with congestive hepatopathy had the highest rates of hypertension/antihypertensive (47.6%) and CVD conditions (23.6%), while those with NAFLD had the highest rates of diabetes (8.4%) (online supplemental table 5-2).

**Table 4:** Comorbidities associated with liver disease

Comorbidities	Full cohort, n=111 098*	Primary care group, n=20 319*	Secondary care group, n=56 710*	Mortality group, n=3459*	Multisource group, n=30 610*
CVD-related conditions	8923 (8.0%)	852 (4.2%)	5362 (9.5%)	385 (11.2%)	2324 (7.6%)
Diabetes	7658 (6.9%)	1271 (6.3%)	3577 (6.3%)	279 (8.1%)	2531 (8.3%)
Hypertension/antihypertensives	40 427 (36.4%)	7248 (35.7%)	20 361 (35.9%)	1368 (39.7%)	11 450 (37.4%)

* n (%).

COPD, chronic obstructive pulmonary disease; CVD, cardiovascular disease.

## Discussion

In this large-scale population-based study, we observed that the incidence of liver disease had increased dramatically in Wales during the past two decades. Notably, NAFLD played a significant role in this rise, with its incidence and proportion increasing sharply.

The rapid increase in the incidence rate of liver disease observed in our study is similar to our previous findings in Wales,^18^ where a 3.6-fold increase was observed in the inpatient chronic liver disease cases between 2001 and 2019, aligning with the trends presented in our secondary care data. This was mainly driven by the 9-fold increase in NAFLD incidence. Other studies, such as P Nasr *et al*’s work in Sweden,^22^ reported a twofold increase in the incidence rate of NAFLD during 2005–2019. Similarly, H Tian *et al*^23^ used data from the Global Burden of Disease study 2019 and showed an increase of 95.4% in NAFLD globally from 1990 to 2019. A systematic review that included 578 studies demonstrated a 13% higher prevalence of NAFLD during the year 2011–2021 compared with year 2000–2010.^24^ A community study in the USA reported a 5-fold increase in the NAFLD incidence from 1997 to 2014.^25^ However, none of these findings compare to our observation in Wales. This substantial difference may be partly attributed to our inclusion of primary care data, which experienced more rapid increase than inpatient data and thus boosted the NAFLD incidence across the study population. The high prevalence of obesity^26^ and rapidly increasing incidence of diabetes^27^ in Wales in the recent decades may also have attributed to the sharp increase of NAFLD, as these metabolic factors including obesity^28 29^ and insulin resistance were considered to be strongly linked to NAFLD due to metabolic dysfunctions.^30 31^ This observation is further supported by our observation of autoimmune liver disease, which is not linked to metabolic factors but experienced a decrease during our study period, highlighting the lifestyle-related nature of the increase in NAFLD. Another interesting observation related to NAFLD is the high proportion of those with comorbidities. NAFLD evolves in those with metabolic syndrome and these comorbidities are used to define the more recently adopted term metabolic-associated fatty liver disease.^32^ In keeping with this definition of NAFLD, we observed a high proportion of individuals having onset comorbidities such as hypertension, diabetes, CVD-related conditions up to 10 years prior to their first liver disease diagnosis. The multimorbidity among people living with liver disease is anticipated considering the increasing incidence of NAFLD and the high prevalence of obesity in Wales, and this will require engagement with primary care practitioners to address their complex multidisciplinary healthcare needs and identify those at risk of significant liver disease early in the disease trajectory.^33^

Contrary to previous European studies showing a decline in ArLD incidence,^18 34^ our research observed a resurgence after 2015 following a slight decrease from 2004 to 2014. We believe that changes in drinking habits^35 36^ and government actions on alcohol pricing and taxation^37^ likely influenced the declining trend before 2015, while the resurgence after 2015 was contributed by the inclusion of outpatient data from the primary care group. The addition of primary care data could explain the discrepancy in our results compared with the prior studies that focused solely on inpatients, filling the gap by providing a broader perspective by including outpatient data.^18 34^ Similar to ArLD, the incidence of HBV increased until 2017, then sharply dropped after the year of 2018. The observed decrease in the incidence of HBV and HCV post-2020 could be attributed to the initiatives outlined in the Welsh Health Circular, which seeks to meet the goal of eradicating HBV and HCV as a major public health concern.^38^ This trend may represent the effort in better case finding and eradication, which led to reduced transmission for HCV and enhanced vaccination and viral suppression for HBV.^39^ Additionally, disruptions caused by the COVID-19 pandemic may also have played a role in this decline.^40^

It is worth to mention our observation on the increasing number of incident cases identified from primary care data, particularly after 2015. This observation, together with the rapid increase in NAFLD and ArLD incidence, indicated an increasing detection rate of liver disease in primary care at an early stage. This change might be attributed to the implementation of the Wales Liver Disease Delivery Plan, which emphasised the importance of early detection and ensured that ‘excellent care’ is accessible when necessary.^41^ Another notable trend observed during our study period is the decreasing proportion of severe late-stage presentations, such as hepatic decompensation. Additionally, we observed a declining trend in the proportion of individuals who died from liver disease without receiving a prior diagnosis. This is to say, despite the increasing incidence of liver disease, there is a promising indication that more individuals were identified as the result of early detection. This provides the healthcare system with more opportunities for early-stage intervention. Early detection of liver disease has been linked to improved long-term outcomes, and a series of reviews and guidelines^42 43^ emphasise the importance of thorough evaluation and timely referral for patients with abnormal liver function tests. Despite these recommendations, adherence remains inconsistent, and the factors influencing clinicians’ referral practices—including potential barriers and motivating factors—are not well understood. This issue is especially pressing in the context of rising obesity rates and the emergence of new weightloss therapies, both of which carry significant implications for liver health. Consequently, further research is essential to assess how integrated care models and early detection strategies might reduce liver disease progression and mortality.

Our study has several strengths. First, it used the national-scale setting and created a population-based cohort with long-term follow-up, which allowed us to maximise the generalisability of our findings to the wider population. Furthermore, by combining primary care, secondary care and mortality data, our study uniquely expands on previous research and addresses a significant gap in understanding liver disease.^10 11^ This novel approach increased our sample size by at least 20%, allowing us to provide a more comprehensive view of liver disease incidence and characteristics. Specifically, this integrated data approach enabled us to (1) capture a broader range of liver disease cases, reducing the likelihood of underestimating case numbers and ensuring a more accurate representation of disease incidence and (2) include patients from diverse healthcare settings, each with unique clinical characteristics, which allowed for a detailed comparison across these settings and a deeper understanding of liver disease in varied clinical contexts. Additionally, a significant strength of the study is the consistency of coding practices in Wales, which have not changed in response to funding incentives.^44^ This stability helps mitigate potential biases and misdiagnoses, providing a more reliable and objective reflection of disease incidence and management trends. Another strength lies in the reproducibility and the use of the SAIL Databank as a research-ready data asset. By incorporating reproducible research pipelines, we standardised and documented the cohort curation process. This enhances the study’s reliability and reproducibility, enabling other researchers to replicate our findings and explore further research questions within the same framework.

Our study is also vulnerable to several limitations. Regulatory laws associated with the SAIL Databank prohibited the inclusion of conditions classified as sensitive in our analysis. For instance, the sensitive Read codes for HBV and HCV could not be extracted along with the rest of the cohort, resulting in potentially incomplete data for these conditions, affecting the completeness and precision of our data analysis. Our study exclusively included individuals who had a history of GP registration and residency information in Wales. Consequently, we likely overlooked individuals who were unable to register with a GP, a subpopulation that represents a group at significant risk for liver disease. The exclusion may also introduce a potential underestimation of the true incidence rate of liver disease in the primary care data. Moreover, there may be changes in diagnostic practices and healthcare-seeking behaviour over the study period that could influence the observed trends and introduce variations, potentially affecting the accuracy of our findings regarding liver disease incidence and management. Furthermore, due to the observational nature and design of our study, we were unable to establish causal relationships between the onset of liver disease and other comorbidities.

## Conclusion

Our study observed a significant rise in the incidence of liver disease in Wales over the past two decades, primarily driven by the increase in NAFLD. The high prevalence of comorbid conditions among liver disease patients and the increased role of primary care in disease identification highlight the need for integrated healthcare approaches to address this growing public health concern. A better understanding of the incidence of liver disease, driven by a more comprehensive analysis of the integrated primary and secondary care data, is essential as a foundation for effective prevention, early detection and targeted interventions to improve patient outcomes.

## Supplementary material

10.1136/bmjopen-2024-093335 online supplemental file 1

## Data Availability

Data may be obtained from a third party and are not publicly available.
