# Correction: Incidence rate and associated patient characteristics of liver disease in Wales 2004–2022: a retrospective population-scale observational study

**DOI:** 10.1136/bmjopen-2024-093335corr1

**Published:** 2025-09-03

 Gao J, Akbari A, Ahmed H, *et al*. Incidence rate and associated patient characteristics of liver disease in Wales 2004–2022: a retrospective population-scale observational study. BMJ Open 2025;15:e093335. doi: 10.1136/bmjopen-2024-0 93 335

This article was previously published with an error.

An error was identified in the alignment of the annual Office for National Statistics (ONS) population estimates. Specifically, population figures for later years were mistakenly applied to earlier years (eg, 2022 population used for 2004), leading to an overestimation of incidence rates.

## Sections Affected

- Abstract – Results
- Results (paragraphs (2–4)) – Updated incidence figures and trend magnitude.
- Discussion (paragraph (2)) – Minor rewording to reflect adjusted trend size.
- Table 2 – Population, crude and standardised incidence rates corrected.
- Online supplemental tables 3 and 4 – Updated with corrected incidence values.
- Figures 2-4 – Regenerated to reflect corrected rates and trends.

**Table 2:** Crude and standardised incidence of chronic liver disease from 2004 to 2022

Year	Wales population	Incident cases	Crude incidence (95% CI)	STD incidence (95% CI)
2004	2 957 422	3060	103.5 (99.8 to 107.2)	110.3 (106.3 to 114.3)
2005	2 969 309	3403	114.6 (110.8 to 118.5)	121.5 (117.4 to 125.8)
2006	2 985 668	3670	122.9 (119.0 to 127.0)	129.8 (125.6 to 134.2)
2007	3 006 299	3537	117.7 (113.8 to 121.6)	123.8 (119.7 to 128.0)
2008	3 025 867	3867	127.8 (123.8 to 131.9)	134.2 (129.9 to 138.5)
2009	3 038 872	4008	131.9 (127.8 to 136.0)	137.5 (133.2 to 141.9)
2010	3 049 971	4268	139.9 (135.8 to 144.2)	145.1 (140.7 to 149.6)
2011	3 063 758	4519	147.5 (143.2 to 151.9)	153.2 (148.7 to 157.7)
2012	3 070 928	4546	148.0 (143.8 to 152.4)	152.6 (148.2 to 157.2)
2013	3 071 058	4733	154.1 (149.8 to 158.6)	158.0 (153.5 to 162.6)
2014	3 073 788	5145	167.4 (162.8 to 172.0)	170.1 (165.5 to 174.9)
2015	3 072 739	5659	184.2 (179.4 to 189.0)	186.2 (181.4 to 191.2)
2016	3 077 165	6548	212.8 (207.7 to 218.0)	215.1 (209.9 to 220.4)
2017	3 081 366	6981	226.6 (221.3 to 231.9)	227.4 (222.1 to 232.9)
2018	3 083 840	7953	257.9 (252.3 to 263.6)	257.1 (251.5 to 262.9)
2019	3 087 732	8481	274.7 (268.9 to 280.6)	272.8 (266.9 to 278.7)
2020	3 104 483	7327	236.0 (230.6 to 241.5)	233.6 (228.2 to 239.1)
2021	3 105 633	9136	294.2 (288.2 to 300.3)	288.6 (282.6 to 294.6)
2022	3 131 640	8596	274.5 (268.7 to 280.4)	269.5 (263.8 to 275.3)

**Figure 2:**
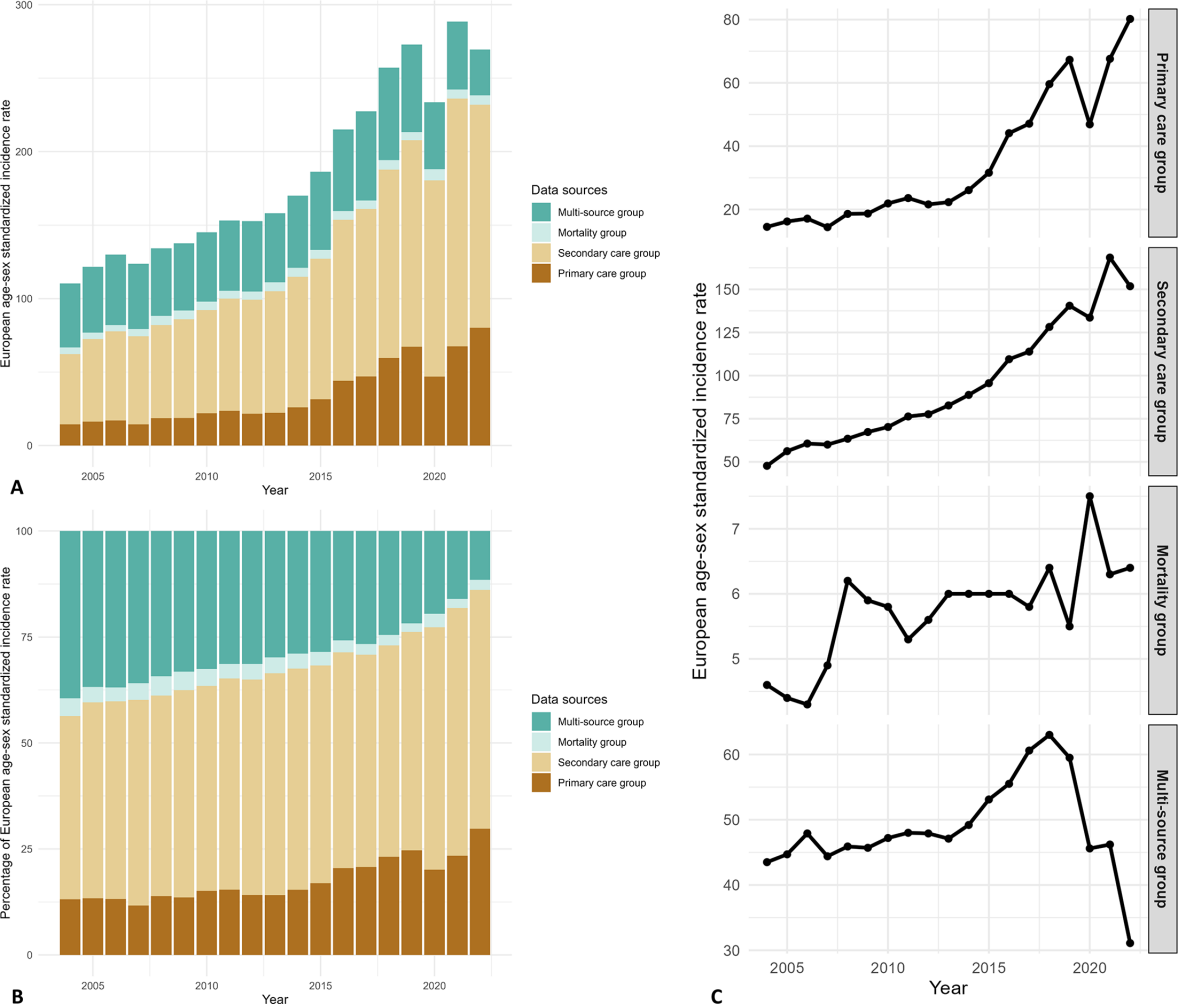
Changes in standardised incidence per 100 000 inhabitants by data source (2004–2022). Bar chart presenting the changes in annual European age-sex standardised incidence rate and percentages by data sources. The x-axis represents time in years, and the y-axis represents the incidence rate in (A), percentages in (B). Line chart presenting the trends in annual European age-sex standardised incidence rate. The x-axis represents time in years, and the y-axis represents incidence rate in (C).

**Figure 3:**
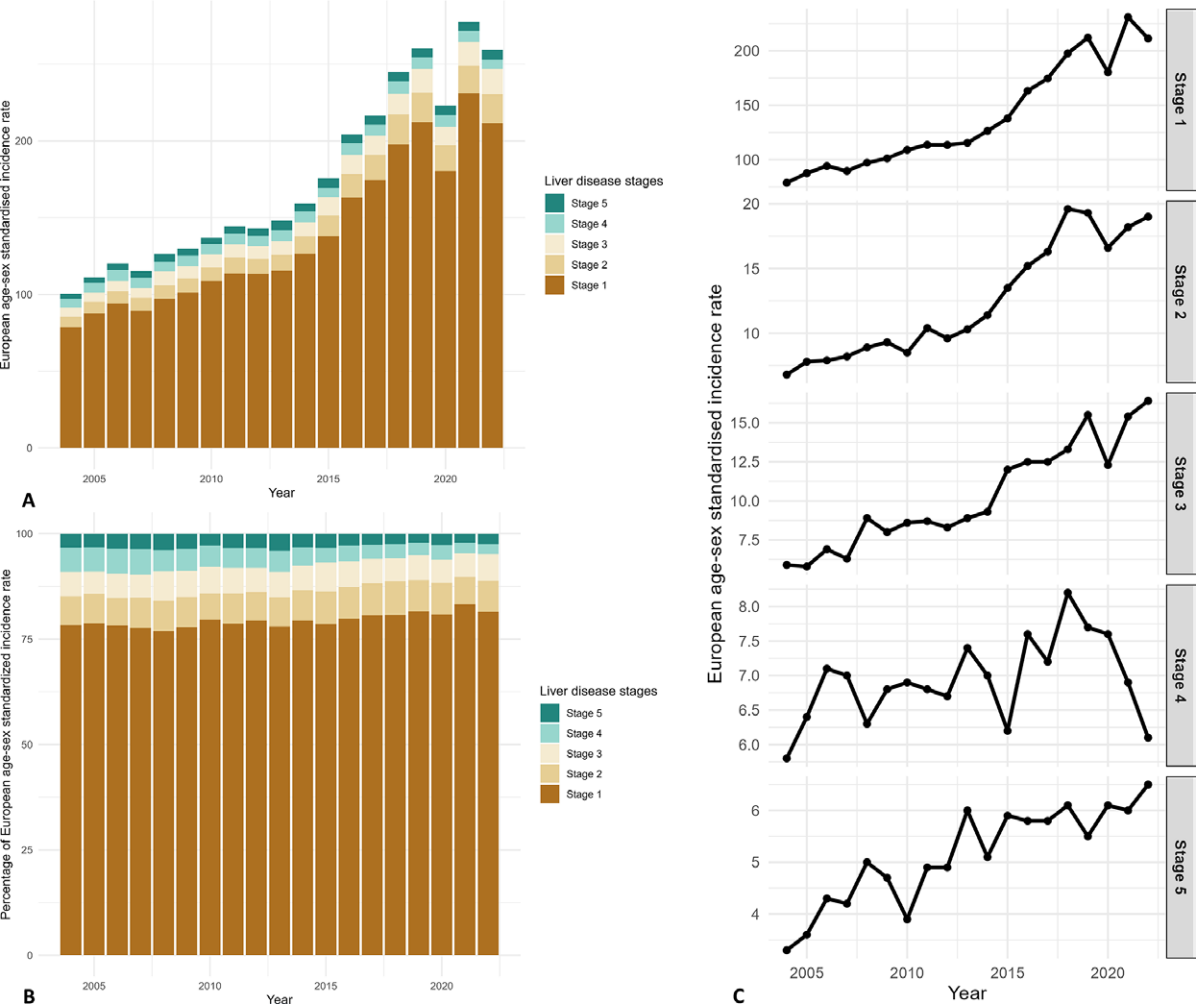
Changes in standardised incidence per 100 000 inhabitants by stages (2004–2022). Bar chart presenting the changes in annual European age-sex standardised incidence rate and percentages by stages. The x-axis represents time in years, and the y-axis represents the incidence rate in (A), percentages in (B). Line chart presenting the trends in annual European age-sex standardised incidence rate. The x-axis represents time in years, and the y-axis represents the incidence rate in (C).

**Figure 4:**
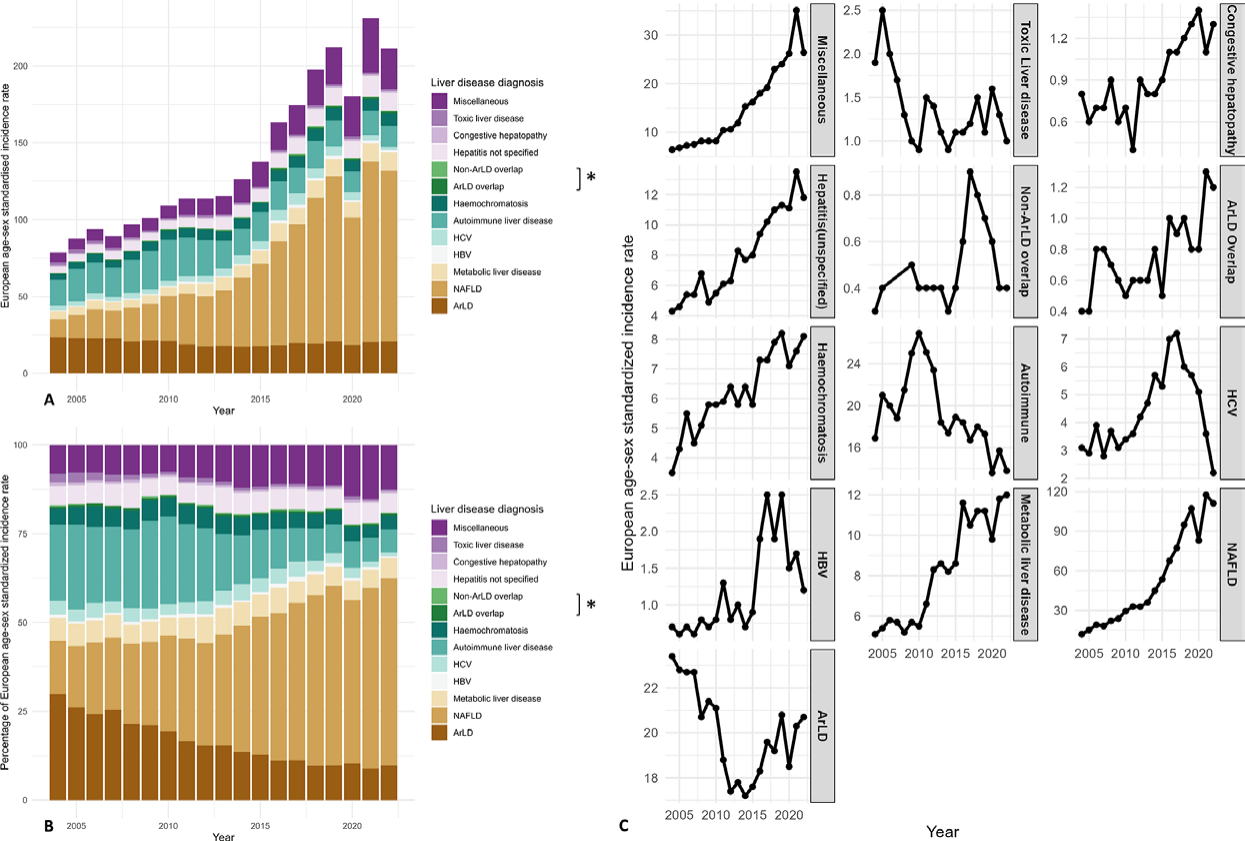
Changes in standardised incidence per 100 000 inhabitants by liver disease aetiologies (2004–2022). Bar chart presenting the changes in annual European age-sex standardised incidence rate and percentages by aetiologies. The x-axis represents time in years, and the y-axis represents the incidence rate in (A), percentages in (B). Line chart presenting the trends in annual European age-sex standardised incidence rate. The x-axis represents time in years, and the y-axis represents the incidence rate in (C). ArLD, alcohol-related liver disease; HBV, hepatitis B virus; HCV, hepatitis C virus; NAFLD, non-alcoholic fatty liver disease. *ArLD overlap: if an individual had two or more diagnoses from liver disease aetiologies (ArLD, NAFLD, HBV, HCV, metabolic, haemochromatosis and autoimmune liver diseases) and one of them was ArLD; non-ArLD overlap: if an individual had two or more diagnoses from liver disease aetiologies but none of them was ArLD.

## Example Correction

Original sentence (Results):

‘The age-sex standardised incidence rate increased by three times during the 18 years of follow-up (97.7 per 100 000 inhabitants in 2004; 316.2 per 100 000 inhabitants in 2022, table 2).’

Corrected sentence:

‘The age-sex standardised incidence rate increased by 2.4 times during the 18 years of follow-up (110.3 per 100 000 inhabitants in 2004; 269.5 per 100 000 inhabitants in 2022, table 2).’

## Impact on Conclusions

The correction does not affect the study’s core conclusions, as the overall trend of increasing liver disease incidence remains. The correction only modifies the magnitude of the change reported.

## Supplementary material

10.1136/bmjopen-2024-093335corr1 online supplemental file 1

