# Supplementary material for: Incidence rate and associated patient characteristics of liver disease in Wales 2004–2022: a retrospective population-scale observational study
Source: BMJ Open. 2025 Feb 10;15(2):e093335. doi: 10.1136/bmjopen-2024-093335 (PMC11815464; doi:10.1136/bmjopen-2024-093335)
Supplement: online supplemental file 1 [file bmjopen-15-2-s001.docx]

**Supplemental Materials**


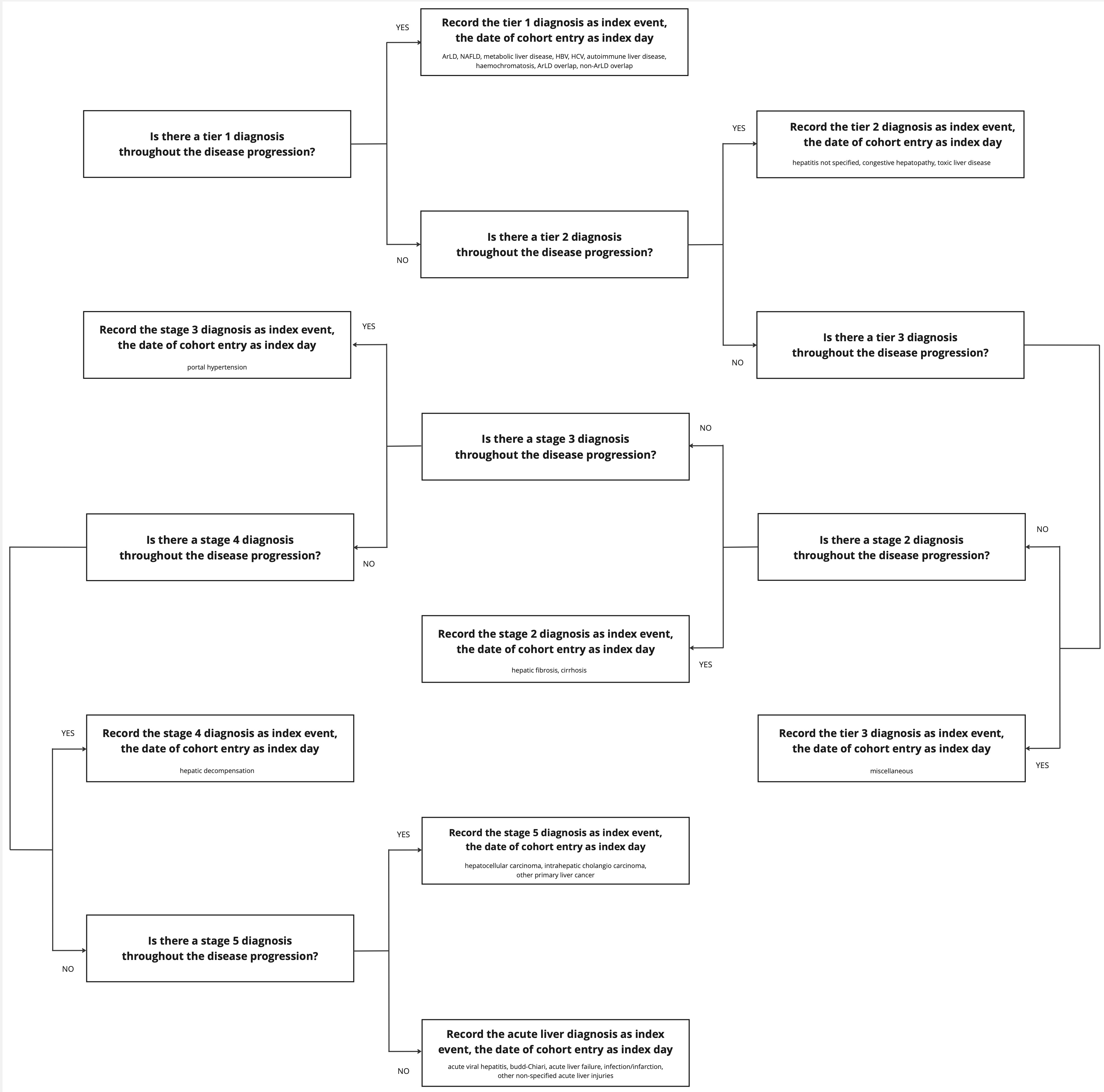


**Supplemental Fig. 1 Flowchart for Identifying Index Events and Determining Cohort Entry Date.**

This flowchart illustrates the decision rules for identifying the index event and determining the cohort entry date for study participants. The index date is defined as the date of the first diagnosis between 2004 and 2022. The aetiological diagnoses throughout the disease progression were identified as the index event entering the cohort. If no aetiological diagnosis is present, the index event is defined based on the following sequence: stage 2 diagnoses, stage 3 diagnoses, stage 4 diagnoses, and stage 5 diagnoses.

The decision flow proceeds as follows:

**Tier 1 Diagnosis**: If present at any point during the disease progression, the tier 1 diagnosis is recorded as the index event, and the date of the cohort entry is the index day. Tier 1 diagnoses include ArLD, NAFLD, metabolic liver disease, HBV, HCV, autoimmune liver disease, and haemochromatosis.

**Tier 2 Diagnosis**: If there is no tier 1 diagnosis, the presence of a tier 2 diagnosis is checked. If present, it is recorded as the index event, and the date of cohort entry is the index day. Tier 2 diagnoses include hepatitis not specified, congestive hepatopathy, and toxic liver disease.

**Tier 3 Diagnosis**: In the absence of tier 1 and tier 2 diagnoses, the presence of a tier 3 diagnosis is checked. If found, it is recorded as the index event. Tier 3 diagnoses include miscellaneous conditions.

**Stage 2 Diagnosis**: If none of the Stage 1 diagnoses are present, the presence of a stage 2 diagnosis is considered. If found, it is recorded as the index event. Stage 2 diagnoses include hepatic fibrosis and cirrhosis.

**Stage 3 Diagnosis**: If no stage 1 or stage 2 diagnoses are present, a stage 3 diagnosis is checked next. If found, it is recorded as the index event. Stage 3 diagnoses include portal hypertension.

**Stage 4 Diagnosis**: If the stage 1, stage 2, and stage 3 diagnoses are absent, a stage 4 diagnosis is checked. If present, it is recorded as the index event. Stage 4 diagnoses include hepatic decompensation.

**Stage 5 Diagnosis**: If no above diagnoses are found, a stage 5 diagnosis is considered. If found, it is recorded as the index event. Stage 5 diagnoses include hepatocellular carcinoma, intrahepatic cholangiocarcinoma, other primary liver cancer.

**Acute Liver Diagnosis**: If none of the chronic diagnoses are present, an acute liver diagnosis is checked. If found, it is recorded as the index event. Acute liver diagnoses include acute viral hepatitis, Budd-Chiari, acute liver failure, infections/sepsis, and other non-specified acute liver injuries.

**Supplemental Tables**

Supplemental Table 1 Code list for identifying liver disease

| Phenotype | ICD10 codes | Read codes |
| --- | --- | --- |
| Acute viral hepatitis | B15, B19, B16, B17 (B171 excluded), B172, B178, B179, B180, B159, B169, B199 | A70z1, AyuB0, XE2u., Q4090, A700., A701., A7052, A70.., A706., A708., A709., A70z., A70G., A704., AyuB3 |
| Acute liver failure | K720 | J6000 |
| Budd-Chiari | I820 | G820. |
| Infection/infarction | K750, K763, K751 | J62.., J620., J6200, J6201, J6202, J6203, J6204, J620z, A053., J634., J621. |
| Other non-specified acute liver injuries | K752 | J63y1 |
| Autoimmune liver disease | K754, K743, K831, K753 | J63B., J6141, J6160, J6617, J63X. |
| Haemochromatosis | E831 | C3500 |
| Metabolic liver disease | E880, E830 | C3762, C3761, C3510 |
| HBV ^*^ | B181, B180 |  |
| HCV ^*^ | B182 | A70E., A70F. |
| Alcohol-related liver disease | K70 | J613., J6130, J612., J6120, J610., J617., J6170, J611. |
| Non-alcoholic fatty liver disease | K760, K7581 | J61y1, J61y8 |
| Hepatitis not specified | K769, K7589, K73 | Jyu72, J614y |
| Congestive hepatopathy | K761, K762, K765 | J630., J636., J637. |
| Toxic liver disease | K71 | J635., J6350, J6351, J63252, J6353, J6354, J6355, J6356, J6357, J635X |
| Miscellaneous | K764, K768, K77 | J638., Jyu73, J63yz, Jyu75 |
| Hepatic fibrosis | K740, K741, K742 | J61y4, J61y6, J61y5 |
| Cirrhosis | K703, K744, K745, K746, K749 | J6161, J616z, J615z |
| Portal hypertension | K766, I81, I859, I982, I85 | J623., G81.., G8523, G852., G8521, G8522, G852z |
| Hepatic decompensation | K721, K767, I850, K72, C220 | J624., SP143, G850., G8520, J625., B1503, BB5D7, BB5D5, BB5D8 |
| Hepatocellular carcinoma (HCC) | C220 | B1503, BB5D7, BB5D5, BB5D8 |
| Intrahepatic cholangio carcinoma (ICC) | C221 | B150. |
| Other primary liver cancers | C222, C223, C224, C225, C226. C227 | B808. |

*We identified Read codes(A7071, A7073, ZV02B, Q4091, 43B4., A7070, A7051, A7072, A70z0, A70A., A70B., A70C., A70D., A70E.,A70F., ZV02C) and ICD-10 codes (B180, B181, B182) for HBV and HCV. However, in order to comply with Data Protection Act 2018 and the UK General Data Protection Regulation, we could not include Read codes (A7071, A7073, ZV02B, Q4091, 43B4., A7070, A7051, A7072, A70z0, A70A., A70B., A70C., A70D., ZV02C) and ICD-10 codes (B171) as these were flagged as sensitive in the latest version of known sensitive code list of SAIL Databank.

Supplemental Table 2-1 ICD-10 code list for identifying comorbidities

| Comorbidities | ICD-10 codes |
| --- | --- |
| Atrial fibrillation | I481, I482 |
| Angina | I200, I201, I208, I209 |
| Asthma | J45 |
| Diabetes | E09, E10, E102, E103, E11, E13, K86 |
| Heart failure | I50, I501, I502, I503, I508 |
| Hypertension | I10, I11, I12, I13, I15 |
| Peripheral vascular disease | E106, E116, I70 |
| Renal disease | N18 |
| Stroke | I691, I61, I63, I64, I60, I66, G45.0, G45.1, G45.3, G46.0, G46.2, G45.8, I65, G46.1, 45.9, G45.2, G45.4 |
| Transient ischaemic attack | I61, I63, I66 |
| Other ischaemic | I20, I21, I22, I23, I24, I25 |

Supplemental Table 2-2 Read codes list for identifying comorbidities

| Comorbidities | Read codes |
| --- | --- |
| Atrial fibrillation | 14AN., 14AR., 3272, 3273, 8CMW2, G573., G5730, G5731, G5732, G5733, G5734, G5735, G5736, G5737, G5738, G5739, G573z, 3272, 3273, 793M1, 793M3 |
| Angina | G3112, G33.., G330., G3300, G330z, G33z., G33z3, G33z7, G33zz, 662K., 662K0, 662K1, 662K2, 662Kz, 8B27., G33z1, G33z2, G33z5, G33z6, G34y0, Gyu30 |
| Asthma | H33.., H330., H3300, H3301, H330z, H331., H3310, H3311, H331z, H332., H333., H334., H335., H33z., H33z0, H33z1, H33z2, H33zz, |
| Diabetes | 66AJ., 66AJ1, 66AJz, 66An., 66Ao., 8CR2., 9OLA., 9OLA., C10.., C100., C1000, C100z, C101., C1010, C1011, C101y, C102., C1020, C1021, C102z, C103., C1030, C1031, C103y, C103z, C104., C1040, C1041, C104y, C104z, C105., C1050, C1051, C105y, C105z, C106., C1060, C1061, C106y, C106z, C107., C1070, C1071, C1072, C1073, C1074, C107y, C107z, C108., C1080, C1081, C1082, C1083, C1084, C1085, C1086, C1087, C1088, C1089, C108A, C108B, C108C, C108D, C108E, C108F, C108G, C108H, C108J, C108y, C108z, C109., C1090, C1091, C1092, C1093, C1094, C1095, C1096, C1097, C1099, C109A, C109B, C109C, C109D, C109E, C109F, C109G, C109H, C109J, C109K, C10A., C10A0, C10A1, C10A2, C10A3, C10A4, C10A5, C10A6, C10A7, C10AW, C10AX, C10B., C10B0, C10C., C10D., C10E., C10E0, C10E1, C10E2, C10E3, C10E4, C10E5, C10E6, C10E7, C10E8, C10E9, C10EA, C10EB, C10EC, C10ED, C10EE, C10EF, C10EG, C10EH, C10EJ, C10EK, C10EL, C10EM, C10EN, C10EP, C10EP, C10EQ, C10ER, C10F., C10F0, C10F1 C10F2, C10F3, C10F4, C10F5, C10F6, C10F7, C10F9, C10FA, C10FB, C10FC, C10FD, C10FE, C10FF, C10FG, C10FH, C10FJ, C10FK, C10FL, C10FM, C10FN, C10FP, C10FQ, C10FR, C10FS, C10G., C10G0, C10H., C10H0, C10K., C10K0, C10M., C10M0, C10N., C10N0, C10N1, C10P., C10P0, C10P1, C10y., C10y1, C10yy, C10yz, C10z., C10z0, C10z1, C10zy, C10zz, F372., F3720, F3721, F3722, 1434, 14F4., 14P3., 9OL9. |
| Heart failure | G58.., G580., G5800, G5801, G5802, G5803, G5804, G581., G5810, G582., G583., G584., G58z., G232., G234., G1yz1, 1O1.., 662W., 662p., 8B29., 8H2S., 9Or0., G400., G41z., G5540, G5540, G5yy9, G5yyA, R2y10, 585f., 585g., 14A6., 14AM., 1736, 1J60., 23E1., 388D., 662T., 662f., 662g., 662h., 662i., 679X., 8CL3., 8HBE., 8HHz., 8Hg8., 8Hk0., 9N0k., 9N2p., 9N4s., 9N4w., 9N6T., 9On.., 9On0., 9On1., 9On2., 9On3., 9On4., 9Or.., 9Or1., 9Or2., 9Or3., 9Or4., 9Or5., 9h1.., 9h11., 9h12., 9hH.., 9hH0., 9hH1., G581., H54.., H541., H5410, H541z, H54z., H584., H584z, ZRad. |
| Hypertension | 14A2., G2..., G20.., G200., G201., G202., G203., G20z., G21.., G210., G2100, G2101, G210z, G211., G2110, G2111, G211z, G21z., G21z0, G21z1, G21zz, G22.., G220., G221., G222., G22z., G23.., G230., G231., G232., G233., G234., G23z., G24.., G240., G2400, G240z, G241., G2410, G241z, G244., G24z., G24z0, G24z1, G24zz, G25.., G250., G251., G26.., G27.., G28.., G2y.., G2z.., 6627, 6628, 662F., 662G., 662O., 662b., 662c., 662d., 662r., 7Q01., 8B26., 8BL0., 8I3N., F4042, F4213, G672., Gyu2., L122., L1220, L1221, L1223, L122z, L127., L127z, L128., L1280, L1282, Gyu21 |
| Peripheral vascular disease | G73.., G734., G73y., G73z., G73z0, G73zz, Gyu74, 2G63., A3A0F, C107., C1070, C1071, C1073, C1074, C107z, C108G, C109F, C109F, C10EG, C10FF, G700., G702., G702z, G731., G7310, G731z, G732., G7320, G7321, G733., G73y0, G73y1, G73yz, G740., G742z, M271., M2710, M2713, R0550, R0550 |
| Renal disease | 1Z13., 1Z14., 1Z1H., 1Z1J., 1Z1K., 1Z1L., K050., K054., K055., K060., K060., K08z., K0D.., 1Z10., 1Z17., 1Z18., 1Z11., 1Z19., 1Z1A., 1Z12., 1Z15., 1Z16., 1Z1B., 1Z1C., 1Z1D., 1Z1E., 1Z1F., 1Z1G., |
| Stroke | G6..., G61.., G610., G611., G612., G613., G614., G615., G616., G617., G618., G619., G61X., G61X0, G61X1, G61z., G63.., G630., G631., G632., G633., G634., G63y., G63y0, G63y1, G63z., G64.., G640., G6400, G641., G6410, G64z., G64z0, G64z1, G64z2, G64z3, G64z4, G66.., G660., G661., G662., G663., G664., G665., G666., G667., G668., G67.., G670., G671., G6710, G6711, G671z, G677., G6770, G6771, G6772, G6773, G6774, G679., G67y., G67z., G6y.., G67z. |
| Transient ischaemic attack | G65z., G65zz, G65z1, G65y., 14AB., G65z0, Fyu55, G65.., G65.., G650., G651., G6510, G652., G653., G654., G655., G656., G657., G65y., F4236, 14AB0 |
| Other ischaemic | G33z4, G34.., G34y., G34y0, G34y1, G34yz, G34z., G34z0, G3y.., G3z.., G31y3, G332., 6A2.., 6A4.., 8B3k., 8H2V., G3..., G31.., G3110, G31y., G31y2, G31yz, G340., G343., G344., Gyu3., Gyu32, Gyu33 |
| Anti-hypertensive | bi1.., bi11., bi12., bi13., bi14., bi15., bi16., bi17., bi18., bi19., bi1A., bi1B., bi1C., bi1H., bi1I., bi1J., bi1K., bi1a., bi1b., bi1c., bi1d., bi1g., bi1h., bi1i., bi1j., bi1k., bi1l., bi1m., bi1n., bi1o., bi1p., bi1q., bi1r., bi1v., bi1w., bi1x., bi1y., bi1z., bi2.., bi21., bi22., bi23., bi24., bi25., bi26., bi27., bi29., bi2A., bi2B., bi2C., bi2D., bi2E., bi2F., bi2G., bi2H., bi2J., bi2K., bi2L., bi2M., bi2a., bi2t., bi2u., bi2v., bi2w., bi2x., bi2y., bi2z., bi3.., bi31., bi32., bi33., bi34., bi35., bi36., bi37., bi38., bi39., bi3a., bi3b., bi3c., bi3c., bi3d., bi3d., bi3e., bi3f., bi3g., bi3h., bi3i., bi3j., bi3k., bi3l., bi3m., bi3q., bi3r., bi3y., bi4.., bi41., bi42., bi43., bi44., bi45., bi46., bi47., bi49., bi4A., bi4B., bi4C., bi4D., bi4E., bi5.., bi51., bi52., bi53., bi54., bi57., bi58., bi6.., bi61., bi62., bi63., bi64., bi65., bi66., bi67., bi68., bi69., bi6A., bi6B., bi6C., bi6D., bi6E., bi6F., bi6G., bi6o., bi6p., bi6q., bi6r., bi6s., bi6t., bi6u., bi6v., bi6w., bi6x., bi6y., bi6z., bi7.., bi71., bi72., bi73., bi74., bi8.., bi81., bi82., bi82., bi83., bi83., bi84., bi84., bi85., bi86., bi86., bi87., bi87., bi88., bi88., bi89., bi89., bi8a., bi9.., bi91., bi92., bi93., bi94., bi94., bi95., bi95., bi96., bi96., bi97., bi98., bi99., bi9A., bi9A., bi9z., biA.., biA1., biA2., biA3., biA4., biB.., biB1., biB2., biB3., biBx., biBy., biBz., biC.., biC1., biC2., biC3., biC4., biC5., biC6., bk3.., bk31., bk32., bk33., bk34., bk37., bk38., bk3B., bk3C., bk3D., bk3E., bk3F., bk3G., bk3H., bk4.., bk41., bk42., bk43., bk44., bk45., bk46., bk4A., bk4B., bk4C., bk4s., bk4t., bk4u., bk4v., bk4w., bk5.., bk51., bk52., bk53., bk54., bk55., bk56., bk7.., bk71., bk72., bk73., bk74., bk75., bk76., bk77., bk78., bk79., bk7z., bk8.., bk81., bk82., bk83., bk84., bk85., bk8z., bk9.., bk91., bk92., bk92., bk93., bk9x., bk9y., bk9z., bkB.., bkB1., bkB2., bkB3., bkB4., bkB5., bkB6., bkJ.., bkJ1., bkJ2., bkJ3., bkJ4., bkJ5., bkJ6., bdc, bd1.., bd11., bd12., bd13., bd14., bd15., bd16., bd17., bd18., bd19., bd1A., bd1B., bd1C., bd1D., bd1E., bd1F., bd1G., bd1I., bd1J., bd1K., bd1L., bd1M., bd1N., bd1O., bd1P., bd1Q., bd1R., bd1S., bd1T., bd1U., bd1V., bd1W., bd1X., bd1Y., bd1Z., bd1a., bd1b., bd1c., bd1d., bd1e., bd1f., bd1g., bd1h., bd1i., bd1j., bd1k., bd1l., bd1l., bd1m., bd1n., bd1o., bd1p., bd1r., bd1r., bd1s., bd1t., bd1u., bd1v., bd1w., bd1x., bd1y., bd1z., bd2.., bd21., bd22., bd23., bd2w., bd2x., bd2y., bd3.., bd31., bd32., bd34., bd35., bd36., bd37., bd3a., bd3b., bd3c., bd3c., bd3d., bd3e., bd3f., bd3g., bd3h., bd3i., bd3j., bd3k., bd3l., bd3x., bd3z., bd4.., bd41., bd4z., bd5.., bd51., bd52., bd53., bd54., bd55., bd56., bd57., bd58., bd59., bd5a., bd5t., bd5u., bd5v., bd5w., bd5x., bd5y., bd6.., bd61., bd62., bd64., bd65., bd66., bd67., bd68., bd6b., bd6c., bd6d., bd6e., bd6w., bd6x., bd6z., bd7.., bd71., bd72., bd7y., bd7z., bd8.., bd81., bd82., bd83., bd84., bd85., bd86., bd87., bd88., bd89., bd8a., bd8b., bd8c., bd8d., bd8e., bd8f., bd8g., bd8h., bd8i., bd8k., bd8l., bd8m., bd8n., bd8o., bd8u., bd9.., bda.., bda1., bda2., bda3., bda4., bday., bdaz., bdb.., bdc.., bdc1., bdc1., bdc2., bdc3., bdc4., bdc5., bdcu., bdcv., bdcw., bdcx., bdd.., bdd1., bdd1., bdd2., bddz., bde.., bde1., bde2., bde3., bde4., bde5., bde6., bde7., bde8., bde9., bdeQ., bdeR., bdea., bdeb., bdec., bded., bdee., bdef., bdeg., bdeh., bdei., bdej., bdek., bdel., bdf.., bdf1., bdf2., bdf3., bdf4., bdf5., bdf5., bdf6., bdf6., bdf7., bdf8., bdf9., bdfA., bdfB., bdfC., bdfD., bdfE., bdfF., bdfG., bdfH., bdfI., bdfJ., bdfK., bdfL., bdfM., bdfw., bdfx., bdfy., bdfz., bdg.., bdg1., bdg2., bdh.., bdh1., bdh2., bdh3., bdh4., bdi.., bdi1., bdi2., bdj.., bdj1., bdj2., bdj3., bdj4., bdj5., bdl.., bdl1., bdl1., bdl2., bdl3., bdl4., bdl5., bdl6., bdl7., bdl8., bdl8., bdm.., bdm1., bdm2., bdmy., bdmz., bdn.., bdn1., bdn2., bdn3., bdn4., bdn5., bdn6., bb3.., bb31., bb32., bb33., bb34., bb35., bb36., bb37., bb38., bb39., bb3A., bb3B., bb3C., bb3D., bb3F., bb3G., bb3H., bb3J., bb3K., bb3L., bb3M., bb3N., bb3O., bb3P., bb3Q., bb3a., bb3b., bb3d., bb3e., bb3f., bb3g., bb3h., bb3i., bb3j., bb3k., bb3l., bb3m., bb3n., bb3p., bb3q., bb3r., bb3s., bb3v., bb3w., bb3x., bb3y., bb3z., bl5.., bl51., bl52., bl53., bl54., bl55., bl56., bl57., bl58., bl59., bl5A., bl5B., bl5C., bl5D., bl5E., bl5F., bl5G., bl5H., bl5I., bl5J., bl5K., bl5L., bl5M., bl5N., bl5O., bl5P., bl5Q., bl5R., bl5S., bl5T., bl5U., bl5V., bl5V., bl5W., bl5W., bl5X., bl5Y., bl5Z., bl5a., bl5b., bl5c., bl5d., bl5e., bl5f., bl5g., bl5h., bl5j., bl5k., bl5l., bl5m., bl5n., bl5o., bl5p., bl5q., bl5r., bl5s., bl5t., bl5u., bl5v., bl5w., bl5x., bl5y., bl5z., bl7.., bl71., bl72., bl73., bl74., bl7w., bl7x., bl7y., bl7z., bl8.., bl81., bl82., bl83., bl84., bl85., bl86., bl89., bl8A., bl8B., bl8C., bl8D., bl8E., bl8F., bl8G., bl8H., bl8J., bl8K., bl8L., bl8M., bl8O., bl8P., bl8Q., bl8R., bl8S., bl8T., bl8U., bl8V., bl8W., bl8X., bl8Y., bl8Z., bl8a., bl8b., bl8c., bl8d., bl8e., bl8f., bl8g., bl8h., bl8i., bl8j., bl8k., bl8l., bl8m., bl8n., bl8o., bl8p., bl8q., bl8r., bl8s., bl8t., bl8u., bl8v., bl8w., bl8x., bl8y., bl8z., bla.., bla.., bla1., bla1., bla2., bla2., blb.., blb1., blb2., blb3., blb4., blb5., blb5., blb6., blb6., blb7., blb8., blc.., blc1., blc2., blc3., blc4., blc5., blc6., blc7., blc8., blc9., blca., blcb., blcc., blcd., blce., blcf., blcg., blch., blci., blcj., blck., blcl., blcm., blcn., blco., blcp., blcq., blcr., blcs., blct., ble.., ble1., ble2., ble3., ble4., ble5., blg.., blg1., blg2., blg3., blg4., blg5., blg6., blh.., blh1., blh2., blh3., blh4., blj.., blj1., blj2., blj3., blj4., blj5., blj6., blj7., blj8., blj9., bljA., bljB., bljC., bljD., bljE., bljF., bljG., bljH., bljJ., bljK., bljL., bljM., bljN., bljO., bljP., bljQ., bljR., bljS., bljT., bljU., bljV., bljW., bljX., bljY., bljZ., blja., bljb., bljc., bljd., blje., bljf., bll.., bll1., bll2., bll3., bll4., bll5., bll6., bll7., bll8., bll9., blla., bllb., bllc., blld., blle., bllf., bllg., bllh., blli., bllj., bllk., blll., dt1.., dt13., dt14., b2..., b21.., b211., b212., b213., b214., b215., b216., b217., b218., b219., b21A., b21B., b21a., b21b., b22.., b221., b222., b22y., b22z., b23.., b231., b232., b23y., b23z., b24.., b25.., b251., b25z., b26.., b261., b262., b263., b264., b26y., b26z., b27.., b271., b27z., b28.., b281., b282., b283., b284., b285., b286., b287., b288., b289., b28z., b29.., b291., b29z., b2a.., b2a1., b2az., b2b.., b2b1., b2b2., b2b3., b2bz., b2c.., b2c1., b2cz., b2d.., b2d1., b2dz., bA1.., bA11., bA12., bA1y., bA1z., bi1D., bi1E., bi1F., bi1G., bi1e., bi1f., bi1s., bi28., bi2b., bi3n., bi3p., bi3s., bi3t., bi3u., bi3v., bi3w., bi3x., bi48., bi4F., bi55., bi56., biC7., biC8., bk35., bk36., bk39., bk3A., bk3y., bk3z., bk47., bk48., bk49., bk4x., bk4y., bk4z., bk57., bk58., bk59., bk5x., bk5y., bk5z., bk86., bk87., bk88., bk8w., bk8x., bk8y., bkC.., bkC1., bkC2., bkC3., bkCx., bkCy., bkCz., bkH.., bkH1., bkH2., bkH3., bkHx., bkHy., bkHz., bkI.., bkI1., bkI2., bkI3., bkI4., bkI5., bkL.., bkL1., bkL2., bkL3., bkL4., bkL5., bkL6., bd38., bd39., bdeA., bdeB., bdeC., bdeD., bdeE., bdeF., bdeG., bdeH., bdeJ., bdeK., bdeL., bdeM., bdeN., bdeO., bdeP., bdem., bden., bdeo., bdep., bdeq., bder., bdes., bdes., bdet., bdeu., bdev., bdew., bdex., bdey., bdez., bl5i., bh4.., bh5y., bh56., bh41., bh4x., bh5z., bh63., bh55., bh54., bh6B., bh1y., bh65., bh4z., bh14., bh4D., bh6A., bh68., bh4v., bh4B., bh6F., bh61., bh69., bh46., bh21., bh6E., bh4y., bh45., bh47., bh6H., bh42., bh6y., bh1z., bh5.., bh6C., bh6D., bh57., bh4C., bh4A., bh6G., bh66., bh1.., bh4w., bh53., bh44., bh52., bh43., bh5x., bh6z., bh13., bh51., bh64., bh67., bh49., bh48., bh11., bh2y., bh6.., bh12., bh62., bf39., bf26., bf1w., bf1x., bf35., bf3b., bf42., bf44., bf3a., bf4.., bf2.., bf2d., bf22., bf2v., bf13., bf2c., bf27., bf2j., bf23., bf24., bf12., bf3d., bf25., bf3c., bf31., bf43., bf36., bf2e., bf33., bf2b., bf2g., bf21., bf11., bf2h., bf2z., bf32., bf34., bf41., bf29., bf2a., bf2f., bf45., bf37., bf38., bf46., be3x., be2y., be3.., be3z., be3y., be1.., be2x., be22., be32., be21., be31., be2.., be33., |

Supplemental Table 3-1. Standardised incidence rate of liver disease by data sources (2004 to 2022)

| Year | ****PEDW only group**** | | ****WLGP only group**** | | ****ADDE only group**** | | ****Two or more data sources**** | |
| --- | --- | --- | --- | --- | --- | --- | --- | --- |
|  | **STD incidence** | **STD 95% CI** | **STD incidence** | **STD 95% CI** | **STD incidence** | **STD 95% CI** | **STD incidence** | **STD 95% CI** |
| 2004 | 47.7 | ( 45.1 to 50.4 ) | 14.5 | ( 13.1 to 16.0 ) | 4.6 | ( 3.8 to 5.6 ) | 43.5 | ( 41.1 to 46.1 ) |
| 2005 | 56.2 | ( 53.4 to 59.1 ) | 16.2 | ( 14.8 to 17.8 ) | 4.4 | ( 3.6 to 5.3 ) | 44.7 | ( 42.2 to 47.2 ) |
| 2006 | 60.6 | ( 57.7 to 63.6 ) | 17.1 | ( 15.6 to 18.6 ) | 4.3 | ( 3.5 to 5.1 ) | 47.9 | ( 45.4 to 50.5 ) |
| 2007 | 60.0 | ( 57.1 to 63.0 ) | 14.4 | ( 13.1 to 15.9 ) | 4.9 | ( 4.1 to 5.8 ) | 44.4 | ( 42.0 to 47.0 ) |
| 2008 | 63.4 | ( 60.5 to 66.5 ) | 18.6 | ( 17.1 to 20.3 ) | 6.2 | ( 5.3 to 7.3 ) | 45.9 | ( 43.4 to 48.5 ) |
| 2009 | 67.3 | ( 64.3 to 70.4 ) | 18.7 | ( 17.1 to 20.3 ) | 5.9 | ( 5.0 to 6.9 ) | 45.7 | ( 43.2 to 48.2 ) |
| 2010 | 70.2 | ( 67.2 to 73.4 ) | 21.9 | ( 20.2 to 23.6 ) | 5.8 | ( 5.0 to 6.8 ) | 47.2 | ( 44.7 to 49.7 ) |
| 2011 | 76.3 | ( 73.1 to 79.5 ) | 23.6 | ( 21.9 to 25.4 ) | 5.3 | ( 4.5 to 6.2 ) | 48.0 | ( 45.5 to 50.6 ) |
| 2012 | 77.6 | ( 74.4 to 80.8 ) | 21.6 | ( 19.9 to 23.3 ) | 5.6 | ( 4.7 to 6.5 ) | 47.9 | ( 45.5 to 50.5 ) |
| 2013 | 82.7 | ( 79.4 to 86.0 ) | 22.3 | ( 20.6 to 24.1 ) | 6.0 | ( 5.1 to 6.9 ) | 47.1 | ( 44.6 to 49.6 ) |
| 2014 | 88.8 | ( 85.5 to 92.3 ) | 26.1 | ( 24.3 to 28.0 ) | 6.0 | ( 5.2 to 7.0 ) | 49.2 | ( 46.7 to 51.7 ) |
| 2015 | 95.6 | ( 92.1 to 99.1 ) | 31.6 | ( 29.6 to 33.7 ) | 6.0 | ( 5.1 to 6.9 ) | 53.1 | ( 50.5 to 55.7 ) |
| 2016 | 109.5 | ( 105.8 to 113.3 ) | 44.1 | ( 41.8 to 46.6 ) | 6.0 | ( 5.1 to 6.9 ) | 55.5 | ( 52.9 to 58.3 ) |
| 2017 | 113.9 | ( 110.1 to 117.8 ) | 47.1 | ( 44.7 to 49.6 ) | 5.8 | ( 5.0 to 6.7 ) | 60.6 | ( 57.9 to 63.5 ) |
| 2018 | 128.2 | ( 124.2 to 132.3 ) | 59.6 | ( 56.8 to 62.4 ) | 6.4 | ( 5.5 to 7.4 ) | 63.0 | ( 60.2 to 65.9 ) |
| 2019 | 140.5 | ( 136.4 to 144.8 ) | 67.3 | ( 64.4 to 70.2 ) | 5.5 | ( 4.7 to 6.4 ) | 59.5 | ( 56.8 to 62.3 ) |
| 2020 | 133.6 | ( 129.5 to 137.7 ) | 46.9 | ( 44.5 to 49.4 ) | 7.5 | ( 6.6 to 8.5 ) | 45.6 | ( 43.3 to 48.1 ) |
| 2021 | 168.4 | ( 163.9 to 173 ) | 67.6 | ( 64.7 to 70.6 ) | 6.3 | ( 5.5 to 7.3 ) | 46.2 | ( 43.8 to 48.6 ) |
| 2022 | 151.8 | ( 147.6 to 156.2 ) | 80.2 | ( 77.0 to 83.4 ) | 6.4 | ( 5.5 to 7.3 ) | 31.1 | ( 29.2 to 33.1 ) |

Abbreviation: STD: standardized; CI: confidence interval

Supplemental Table 3-2 Standardised incidence rate of liver disease by disease stages (2004 to 2022)

| Year | Stage 1 | | Stage 2 | | Stage 3 | | Stage 4 | | Stage 5 | |
| --- | --- | --- | --- | --- | --- | --- | --- | --- | --- | --- |
|  | **STD incidence** | **STD 95% CI** | **STD incidence** | **STD 95% CI** | **STD incidence** | **STD 95% CI** | **STD incidence** | **STD 95% CI** | **STD incidence** | **STD 95% CI** |
| 2004 | 78.7 | ( 75.4 to 82.1 ) | 6.8 | ( 5.8 to 7.8 ) | 5.9 | ( 5.0 to 6.9 ) | 5.8 | ( 4.9 to 6.8 ) | 3.3 | ( 2.6 to 4.0 ) |
| 2005 | 87.5 | ( 84.0 to 91.0 ) | 7.8 | ( 6.7 to 8.9 ) | 5.8 | ( 5.0 to 6.8 ) | 6.4 | ( 5.4 to 7.5 ) | 3.6 | ( 2.9 to 4.5 ) |
| 2006 | 94.1 | ( 90.5 to 97.8 ) | 7.9 | ( 6.9 to 9.0 ) | 6.9 | ( 6.0 to 8.0 ) | 7.1 | ( 6.1 to 8.1 ) | 4.3 | ( 3.5 to 5.1 ) |
| 2007 | 89.5 | ( 86.0 to 93.1 ) | 8.2 | ( 7.1 to 9.3 ) | 6.3 | ( 5.4 to 7.3 ) | 7.0 | ( 6.1 to 8.1 ) | 4.2 | ( 3.5 to 5.0 ) |
| 2008 | 97.2 | ( 93.6 to 100.9 ) | 8.9 | ( 7.8 to 10.1 ) | 8.9 | ( 7.8 to 10.1 ) | 6.3 | ( 5.4 to 7.3 ) | 5.0 | ( 4.2 to 5.9 ) |
| 2009 | 101.1 | ( 97.5 to 104.9 ) | 9.3 | ( 8.2 to 10.4 ) | 8.0 | ( 7.0 to 9.1 ) | 6.8 | ( 5.9 to 7.8 ) | 4.7 | ( 3.9 to 5.7 ) |
| 2010 | 108.9 | ( 105.1 to 112.8 ) | 8.5 | ( 7.4 to 9.6 ) | 8.6 | ( 7.6 to 9.7 ) | 6.9 | ( 5.9 to 7.9 ) | 3.9 | ( 3.2 to 4.7 ) |
| 2011 | 113.6 | ( 109.7 to 117.5 ) | 10.4 | ( 9.3 to 11.7 ) | 8.7 | ( 7.7 to 9.9 ) | 6.8 | ( 5.8 to 7.8 ) | 4.9 | ( 4.1 to 5.9 ) |
| 2012 | 113.5 | ( 109.7 to 117.5 ) | 9.6 | ( 8.5 to 10.8 ) | 8.3 | ( 7.3 to 9.4 ) | 6.7 | ( 5.8 to 7.7 ) | 4.9 | ( 4.1 to 5.8 ) |
| 2013 | 115.4 | ( 111.6 to 119.4 ) | 10.3 | ( 9.1 to 11.5 ) | 8.9 | ( 7.9 to 10.1 ) | 7.4 | ( 6.4 to 8.5 ) | 6.0 | ( 5.2 to 7.0 ) |
| 2014 | 126.4 | ( 122.4 to 130.5 ) | 11.4 | ( 10.2 to 12.6 ) | 9.3 | ( 8.2 to 10.4 ) | 7.0 | ( 6.1 to 8.0 ) | 5.1 | ( 4.3 to 6.0 ) |
| 2015 | 137.9 | ( 133.7 to 142.2 ) | 13.5 | ( 12.2 to 14.9 ) | 12.0 | ( 10.8 to 13.3 ) | 6.2 | ( 5.4 to 7.2 ) | 5.9 | ( 5.0 to 6.8 ) |
| 2016 | 163.2 | ( 158.7 to 167.9 ) | 15.2 | ( 13.8 to 16.7 ) | 12.5 | ( 11.3 to 13.8 ) | 7.6 | ( 6.7 to 8.7 ) | 5.8 | ( 5.0 to 6.7 ) |
| 2017 | 174.6 | ( 169.9 to 179.4 ) | 16.3 | ( 14.9 to 17.8 ) | 12.5 | ( 11.3 to 13.8 ) | 7.2 | ( 6.3 to 8.2 ) | 5.8 | ( 5.0 to 6.7 ) |
| 2018 | 197.7 | ( 192.7 to 202.7 ) | 19.6 | ( 18.1 to 21.2 ) | 13.3 | ( 12.0 to 14.6 ) | 8.2 | ( 7.2 to 9.2 ) | 6.1 | ( 5.2 to 7.0 ) |
| 2019 | 212.1 | ( 207.0 to 217.4 ) | 19.3 | ( 17.8 to 20.9 ) | 15.5 | ( 14.1 to 16.9 ) | 7.7 | ( 6.7 to 8.7 ) | 5.5 | ( 4.8 to 6.4 ) |
| 2020 | 180.4 | ( 175.7 to 185.3 ) | 16.6 | ( 15.2 to 18.1 ) | 12.3 | ( 11.1 to 13.6 ) | 7.6 | ( 6.6 to 8.6 ) | 6.1 | ( 5.2 to 7.0 ) |
| 2021 | 231.0 | ( 225.6 to 236.4 ) | 18.2 | ( 16.8 to 19.8 ) | 15.4 | ( 14.1 to 16.9 ) | 6.9 | ( 6.1 to 7.9 ) | 6.0 | ( 5.2 to 7.0 ) |
| 2022 | 211.4 | ( 206.3 to 216.5 ) | 19.0 | ( 17.5 to 20.6 ) | 16.4 | ( 15.1 to 17.9 ) | 6.1 | ( 5.3 to 7.0 ) | 6.5 | ( 5.7 to 7.5 ) |

Abbreviation: STD: standardized; CI: confidence interval

Supplemental Table 3-3, Standardised incidence rate of liver disease by aetiologies (2004 to 2022)

| Year | ArLD | NAFLD | Metabolic liver disease | HBV | HCV | Autoimmune liver disease | Haemochromatosis | ArLD overlap | Non-ArLD overlap | Hepatitis not specified | Congestive hepatopathy | Toxic liver disease | Miscellaneous |
| --- | --- | --- | --- | --- | --- | --- | --- | --- | --- | --- | --- | --- | --- |
|  | **STD incidence (95% CI)** | **STD incidence (95% CI)** | **STD incidence (95% CI)** | **STD incidence (95% CI)** | **STD incidence (95% CI)** | **STD incidence (95% CI)** | **STD incidence (95% CI)** | **STD incidence (95% CI)** | **STD incidence (95% CI)** | **STD incidence (95% CI)** | **STD incidence (95% CI)** | **STD incidence (95% CI)** | **STD incidence (95% CI)** |
| 2004 | 23.4 ( 21.7 to 25.3 ) | 11.8 ( 10.6 to 13.2 ) | 5.1 ( 4.3 to 6.1 ) | 0.7 ( 0.4 to 1.0 ) | 3.1 ( 2.5 to 3.9 ) | 16.9 ( 15.4 to 18.6 ) | 3.5 ( 2.8 to 4.2 ) | 0.3 ( 0.2 to 0.6 ) | 0.4 ( 0.2 to 0.8 ) | 4.3 ( 3.5 to 5.2 ) | 0.8 ( 0.5 to 1.3 ) | 1.9 ( 1.4 to 2.5 ) | 6.4 ( 5.5 to 7.4 ) |
| 2005 | 22.8 ( 21.0 to 24.6 ) | 15.2 ( 13.8 to 16.7 ) | 5.4 ( 4.6 to 6.4 ) | 0.6 ( 0.4 to 1.0 ) | 2.9 ( 2.3 to 3.6 ) | 21 ( 19.3 to 22.8 ) | 4.3 ( 3.6 to 5.2 ) | 0.4 ( 0.2 to 0.7 ) | 0.4 ( 0.2 to 0.7 ) | 4.6 ( 3.8 to 5.4 ) | 0.6 ( 0.3 to 1.0 ) | 2.5 ( 2.0 to 3.1 ) | 6.8 ( 5.9 to 7.9 ) |
| 2006 | 22.7 ( 21.0 to 24.5 ) | 19 ( 17.4 to 20.6 ) | 5.8 ( 4.9 to 6.8 ) | 0.7 ( 0.5 to 1.1 ) | 3.9 ( 3.2 to 4.7 ) | 20 ( 18.3 to 21.8 ) | 5.5 ( 4.7 to 6.5 ) | -- | 0.8 ( 0.5 to 1.2 ) | 5.4 ( 4.6 to 6.4 ) | 0.7 ( 0.4 to 1.1 ) | 2 ( 1.5 to 2.6 ) | 7.3 ( 6.3 to 8.4 ) |
| 2007 | 22.7 ( 21.0 to 24.5 ) | 18.1 ( 16.6 to 19.7 ) | 5.7 ( 4.8 to 6.7 ) | 0.6 ( 0.4 to 1.0 ) | 2.8 ( 2.2 to 3.5 ) | 18.8 ( 17.2 to 20.5 ) | 4.5 ( 3.8 to 5.4 ) | -- | 0.8 ( 0.5 to 1.2 ) | 5.4 ( 4.5 to 6.3 ) | 0.7 ( 0.4 to 1.0 ) | 1.7 ( 1.2 to 2.2 ) | 7.5 ( 6.4 to 8.6 ) |
| 2008 | 20.7 ( 19.0 to 22.4 ) | 21.9 ( 20.3 to 23.7 ) | 5.2 ( 4.4 to 6.2 ) | 0.8 ( 0.5 to 1.2 ) | 3.7 ( 3.1 to 4.5 ) | 21.5 ( 19.8 to 23.3 ) | 5.1 ( 4.3 to 6.0 ) | -- | 0.7 ( 0.4 to 1.1 ) | 6.8 ( 5.8 to 7.8 ) | 0.9 ( 0.6 to 1.3 ) | 1.3 ( 0.9 to 1.7 ) | 8.2 ( 7.2 to 9.4 ) |
| 2009 | 21.4 ( 19.7 to 23.1 ) | 23.7 ( 22.0 to 25.5 ) | 5.7 ( 4.9 to 6.7 ) | 0.7 ( 0.4 to 1.1 ) | 3.1 ( 2.5 to 3.8 ) | 25 ( 23.2 to 26.9 ) | 5.8 ( 5.0 to 6.8 ) | 0.5 ( 0.3 to 0.8 ) | 0.6 ( 0.3 to 0.9 ) | 4.9 ( 4.1 to 5.8 ) | 0.6 ( 0.4 to 1.0 ) | 1.0 ( 0.7 to 1.4 ) | 8.2 ( 7.2 to 9.3 ) |
| 2010 | 21.1 ( 19.5 to 22.8 ) | 29.4 ( 27.4 to 31.4 ) | 5.5 ( 4.6 to 6.4 ) | 0.8 ( 0.5 to 1.1 ) | 3.4 ( 2.7 to 4.1 ) | 26.9 ( 25 to 28.9 ) | 5.8 ( 4.9 to 6.7 ) | 0.4 ( 0.2 to 0.6 ) | 0.5 ( 0.2 to 0.8 ) | 5.5 ( 4.7 to 6.5 ) | 0.7 ( 0.4 to 1.1 ) | 0.9 ( 0.6 to 1.3 ) | 8.2 ( 7.2 to 9.4 ) |
| 2011 | 18.8 ( 17.3 to 20.4 ) | 32.9 ( 30.9 to 35.0 ) | 6.6 ( 5.7 to 7.6 ) | 1.3 ( 0.9 to 1.7 ) | 3.6 ( 2.9 to 4.3 ) | 25.1 ( 23.3 to 27.0 ) | 5.9 ( 5.1 to 6.9 ) | 0.4 ( 0.2 to 0.7 ) | 0.6 ( 0.4 to 1.0 ) | 6.1 ( 5.2 to 7.1 ) | 0.4 ( 0.2 to 0.8 ) | 1.5 ( 1.1 to 2.0 ) | 10.4 ( 9.2 to 11.6 ) |
| 2012 | 17.4 ( 15.9 to 18.9 ) | 32.8 ( 30.8 to 35.0 ) | 8.3 ( 7.3 to 9.4 ) | 0.8 ( 0.5 to 1.2 ) | 4.2 ( 3.5 to 5.0 ) | 23.4 ( 21.7 to 25.3 ) | 6.4 ( 5.5 to 7.4 ) | 0.4 ( 0.2 to 0.7 ) | 0.6 ( 0.4 to 1.0 ) | 6.3 ( 5.4 to 7.3 ) | 0.9 ( 0.6 to 1.3 ) | 1.4 ( 1.0 to 1.9 ) | 10.6 ( 9.5 to 11.9 ) |
| 2013 | 17.8 ( 16.3 to 19.4 ) | 36 ( 33.9 to 38.2 ) | 8.6 ( 7.5 to 9.7 ) | 1 ( 0.7 to 1.5 ) | 4.7 ( 4 to 5.6 ) | 18.4 ( 16.9 to 20.0 ) | 5.8 ( 5.0 to 6.7 ) | 0.4 ( 0.2 to 0.7 ) | 0.6 ( 0.4 to 1.0 ) | 8.3 ( 7.3 to 9.4 ) | 0.8 ( 0.5 to 1.2 ) | 1.1 ( 0.7 to 1.5 ) | 11.9 ( 10.7 to 13.2 ) |
| 2014 | 17.2 ( 15.8 to 18.8 ) | 44.9 ( 42.5 to 47.4 ) | 8.2 ( 7.2 to 9.3 ) | 0.7 ( 0.4 to 1.1 ) | 5.7 ( 4.9 to 6.7 ) | 17.4 ( 15.9 to 18.9 ) | 6.4 ( 5.5 to 7.4 ) | 0.3 ( 0.2 to 0.6 ) | 0.8 ( 0.5 to 1.1 ) | 7.7 ( 6.7 to 8.7 ) | 0.8 ( 0.6 to 1.2 ) | 0.9 ( 0.6 to 1.4 ) | 15.3 ( 13.9 to 16.7 ) |
| 2015 | 17.6 ( 16.2 to 19.2 ) | 53.6 ( 51.0 to 56.3 ) | 8.6 ( 7.6 to 9.7 ) | 0.9 ( 0.6 to 1.4 ) | 5.3 ( 4.5 to 6.3 ) | 18.9 ( 17.3 to 20.5 ) | 5.8 ( 5.0 to 6.8 ) | 0.4 ( 0.2 to 0.8 ) | 0.5 ( 0.3 to 0.8 ) | 8.0 ( 7.0 to 9.1 ) | 0.9 ( 0.6 to 1.3 ) | 1.1 ( 0.7 to 1.5 ) | 16.2 ( 14.8 to 17.7 ) |
| 2016 | 18.3 ( 16.8 to 19.9 ) | 67.6 ( 64.7 to 70.6 ) | 11.6 ( 10.4 to 12.9 ) | 1.9 ( 1.4 to 2.5 ) | 7.0 ( 6.1 to 8.0 ) | 18.4 ( 16.9 to 20.0 ) | 7.3 ( 6.3 to 8.3 ) | 0.6 ( 0.3 to 0.9 ) | 1.0 ( 0.7 to 1.5 ) | 9.4 ( 8.3 to 10.6 ) | 1.1 ( 0.7 to 1.5 ) | 1.1 ( 0.8 to 1.6 ) | 18.0 ( 16.5 to 19.6 ) |
| 2017 | 19.6 ( 18 to 21.2 ) | 77.3 ( 74.2 to 80.5 ) | 10.5 ( 9.4 to 11.8 ) | 2.5 ( 2.0 to 3.2 ) | 7.2 ( 6.2 to 8.3 ) | 16.7 ( 15.3 to 18.2 ) | 7.3 ( 6.3 to 8.3 ) | 0.9 ( 0.6 to 1.3 ) | 0.9 ( 0.6 to 1.3 ) | 10.2 ( 9.1 to 11.4 ) | 1.1 ( 0.8 to 1.6 ) | 1.2 ( 0.8 to 1.6 ) | 19.2 ( 17.6 to 20.8 ) |
| 2018 | 19.2 ( 17.6 to 20.8 ) | 94.9 ( 91.5 to 98.5 ) | 11.2 ( 10.0 to 12.4 ) | 1.9 ( 1.5 to 2.5 ) | 6.0 ( 5.2 to 7.0 ) | 18.0 ( 16.5 to 19.5 ) | 7.9 ( 7.0 to 9.0 ) | 0.8 ( 0.5 to 1.2 ) | 1.0 ( 0.6 to 1.4 ) | 11.0 ( 9.9 to 12.3 ) | 1.2 ( 0.8 to 1.6 ) | 1.5 ( 1.1 to 2.1 ) | 23.0 ( 21.4 to 24.8 ) |
| 2019 | 20.8 ( 19.2 to 22.5 ) | 107.2 ( 103.6 to 111.0 ) | 11.2 ( 10.0 to 12.4 ) | 2.5 ( 1.9 to 3.1 ) | 5.7 ( 4.9 to 6.7 ) | 17.3 ( 15.9 to 18.8 ) | 8.2 ( 7.2 to 9.3 ) | 0.7 ( 0.4 to 1.1 ) | 0.8 ( 0.5 to 1.2 ) | 11.3 ( 10.2 to 12.6 ) | 1.3 ( 0.9 to 1.7 ) | 1.1 ( 0.8 to 1.6 ) | 24.0 ( 22.3 to 25.8 ) |
| 2020 | 18.5 ( 17 to 20.1 ) | 83 ( 79.8 to 86.3 ) | 9.8 ( 8.7 to 11.0 ) | 1.5 ( 1.1 to 2.1 ) | 5.1 ( 4.3 to 6.1 ) | 13.6 ( 12.4 to 15 ) | 7.1 ( 6.2 to 8.1 ) | 0.6 ( 0.3 to 0.9 ) | 0.8 ( 0.5 to 1.2 ) | 11.1 ( 10.0 to 12.4 ) | 1.4 ( 1.0 to 1.8 ) | 1.6 ( 1.2 to 2.1 ) | 26.2 ( 24.5 to 28.0 ) |
| 2021 | 20.3 ( 18.8 to 22.0 ) | 117.6 ( 113.8 to 121.5 ) | 11.8 ( 10.6 to 13.1 ) | 1.7 ( 1.2 to 2.2 ) | 3.6 ( 3.0 to 4.4 ) | 15.7 ( 14.3 to 17.1 ) | 7.6 ( 6.6 to 8.6 ) | 0.4 ( 0.2 to 0.7 ) | 1.3 ( 0.9 to 1.8 ) | 13.5 ( 12.3 to 14.9 ) | 1.1 ( 0.7 to 1.5 ) | 1.3 ( 0.9 to 1.8 ) | 35.1 ( 33.0 to 37.2 ) |
| 2022 | 20.7 ( 19.1 to 22.4 ) | 111.2 ( 107.5 to 115.0 ) | 12.0 ( 10.8 to 13.2 ) | 1.2 ( 0.8 to 1.6 ) | 2.2 ( 1.7 to 2.9 ) | 13.8 ( 12.6 to 15.2 ) | 8.1 ( 7.1 to 9.2 ) | 0.4 ( 0.2 to 0.6 ) | 1.2 ( 0.8 to 1.7 ) | 11.8 ( 10.7 to 13.1 ) | 1.3 ( 0.9 to 1.8 ) | 1.0 ( 0.7 to 1.5 ) | 26.4 ( 24.7 to 28.2 ) |

Abbreviation: ArLD: alcohol-related liver disease; NAFLD: non-alcohol fatty liver disease; HBV: hepatitis B virus; HCV: hepatitis C virus; STD: standardised; CI: confidence interval

Supplemental Table 4-1, Standardised incidence rate of NAFLD by data sources (2004 to 2022)

| Year | PEDW-only | | WLGP-only | | ADDE-only | | ****Two or more data sources**** | |
| --- | --- | --- | --- | --- | --- | --- | --- | --- |
|  | **STD incidence** | **STD 95% CI** | **STD incidence** | **STD 95% CI** | **STD incidence** | **STD 95% CI** | **STD incidence** | **STD 95% CI** |
| 2004 | 3.8 | ( 3.2 to 4.6 ) | 4.6 | ( 3.8 to 5.5 ) | 0.5 | ( 0.3 to 0.9 ) | 2.9 | ( 2.3 to 3.6 ) |
| 2005 | 5.0 | ( 4.2 to 5.9 ) | 5.6 | ( 4.8 to 6.6 ) | 0.4 | ( 0.2 to 0.7 ) | 4.2 | ( 3.4 to 5.0 ) |
| 2006 | 6.4 | ( 5.5 to 7.4 ) | 7.7 | ( 6.7 to 8.8 ) | -- | -- | 4.6 | ( 3.9 to 5.5 ) |
| 2007 | 6.6 | ( 5.7 to 7.6 ) | 6.9 | ( 6.0 to 8.0 ) | 0.4 | ( 0.2 to 0.7 ) | 4.2 | ( 3.5 to 5.0 ) |
| 2008 | 6.2 | ( 5.4 to 7.2 ) | 10.6 | ( 9.5 to 11.9 ) | 0.7 | ( 0.4 to 1.0 ) | 4.4 | ( 3.7 to 5.2 ) |
| 2009 | 6.8 | ( 5.8 to 7.8 ) | 10.8 | ( 9.6 to 12.0 ) | 0.6 | ( 0.3 to 0.9 ) | 5.6 | ( 4.8 to 6.5 ) |
| 2010 | 7.2 | ( 6.2 to 8.2 ) | 14.9 | ( 13.5 to 16.4 ) | 0.7 | ( 0.4 to 1.0 ) | 6.6 | ( 5.7 to 7.6 ) |
| 2011 | 10.0 | ( 8.9 to 11.2 ) | 15.4 | ( 14.0 to 16.9 ) | 0.4 | ( 0.2 to 0.6 ) | 7.1 | ( 6.2 to 8.1 ) |
| 2012 | 11.7 | ( 10.5 to 13.0 ) | 13.8 | ( 12.5 to 15.2 ) | 0.4 | ( 0.2 to 0.7 ) | 6.9 | ( 6.0 to 7.9 ) |
| 2013 | 13.0 | ( 11.8 to 14.4 ) | 15.2 | ( 13.8 to 16.7 ) | 1.0 | ( 0.7 to 1.4 ) | 6.7 | ( 5.8 to 7.7 ) |
| 2014 | 17.5 | ( 16.0 to 19.0 ) | 18.2 | ( 16.7 to 19.8 ) | 1.1 | ( 0.7 to 1.5 ) | 8.2 | ( 7.2 to 9.3 ) |
| 2015 | 20.6 | ( 19.0 to 22.3 ) | 22.9 | ( 21.2 to 24.7 ) | 1.0 | ( 0.7 to 1.4 ) | 9.1 | ( 8.1 to 10.3 ) |
| 2016 | 23.3 | ( 21.6 to 25.1 ) | 31.8 | ( 29.9 to 33.9 ) | 1.1 | ( 0.8 to 1.6 ) | 11.3 | ( 10.1 to 12.5 ) |
| 2017 | 28.1 | ( 26.3 to 30.1 ) | 34.4 | ( 32.4 to 36.6 ) | 1.2 | ( 0.8 to 1.6 ) | 13.5 | ( 12.3 to 14.9 ) |
| 2018 | 36.5 | ( 34.4 to 38.7 ) | 43.1 | ( 40.8 to 45.6 ) | 1.5 | ( 1.1 to 2.0 ) | 13.8 | ( 12.5 to 15.1 ) |
| 2019 | 41.7 | ( 39.4 to 44.0 ) | 50.5 | ( 48.0 to 53.1 ) | 1.4 | ( 1.0 to 1.9 ) | 13.6 | ( 12.4 to 15.0 ) |
| 2020 | 40.8 | ( 38.6 to 43.2 ) | 32.0 | ( 30.0 to 34.1 ) | 1.8 | ( 1.3 to 2.3 ) | 8.4 | ( 7.4 to 9.5 ) |
| 2021 | 58.2 | ( 55.6 to 61.0 ) | 49.0 | ( 46.5 to 51.5 ) | 1.4 | ( 1.0 to 1.9 ) | 9.0 | ( 8.0 to 10.1 ) |
| 2022 | 52.9 | ( 50.4 to 55.5 ) | 53.6 | ( 51.0 to 56.2 ) | 1.3 | ( 0.9 to 1.8 ) | 3.4 | ( 2.7 to 4.0 ) |

Abbreviation: STD: standardized; CI: confidence interval

Supplemental Table 4-2, Standardised incidence rate of ArLD by data sources (2004 to 2022)

| Year | PEDW-only | | WLGP-only | | ADDE-only | | ****Two or more data sources**** | |
| --- | --- | --- | --- | --- | --- | --- | --- | --- |
|  | **STD incidence** | **STD 95% CI** | **STD incidence** | **STD 95% CI** | **STD incidence** | **STD 95% CI** | **STD incidence** | **STD 95% CI** |
| 2004 | 4.7 | ( 4.0 to 5.6 ) | 3.2 | ( 2.5 to 3.9 ) | -- | -- | 15.2 | ( 13.8 to 16.7 ) |
| 2005 | 5.3 | ( 4.5 to 6.2 ) | 2.8 | ( 2.3 to 3.5 ) | 0.6 | ( 0.3 to 1.0 ) | 14.0 | ( 12.6 to 15.5 ) |
| 2006 | 5.3 | ( 4.5 to 6.2 ) | 2.8 | ( 2.2 to 3.4 ) | 0.4 | ( 0.2 to 0.7 ) | 14.3 | ( 12.9 to 15.7 ) |
| 2007 | 5.6 | ( 4.7 to 6.5 ) | 2.3 | ( 1.8 to 2.9 ) | 0.6 | ( 0.4 to 1.0 ) | 14.2 | ( 12.9 to 15.7 ) |
| 2008 | 4.9 | ( 4.1 to 5.8 ) | 2.1 | ( 1.6 to 2.7 ) | 0.8 | ( 0.5 to 1.2 ) | 12.9 | ( 11.7 to 14.3 ) |
| 2009 | 5.8 | ( 4.9 to 6.7 ) | 2.1 | ( 1.6 to 2.7 ) | 0.9 | ( 0.6 to 1.4 ) | 12.6 | ( 11.3 to 13.9 ) |
| 2010 | 5.2 | ( 4.4 to 6.1 ) | 2.5 | ( 1.9 to 3.1 ) | 0.9 | ( 0.6 to 1.3 ) | 12.6 | ( 11.3 to 13.9 ) |
| 2011 | 4.5 | ( 3.8 to 5.3 ) | 2.5 | ( 1.9 to 3.1 ) | 0.7 | ( 0.4 to 1.1 ) | 11.1 | ( 10.0 to 12.4 ) |
| 2012 | 4.7 | ( 4.0 to 5.6 ) | 1.8 | ( 1.4 to 2.4 ) | 0.7 | ( 0.4 to 1.1 ) | 10.1 | ( 9.0 to 11.3 ) |
| 2013 | 4.8 | ( 4.0 to 5.6 ) | 2.0 | ( 1.5 to 2.6 ) | 0.6 | ( 0.4 to 1.0 ) | 10.4 | ( 9.2 to 11.6 ) |
| 2014 | 4.6 | ( 3.9 to 5.5 ) | 2.2 | ( 1.7 to 2.8 ) | 0.6 | ( 0.4 to 1.0 ) | 9.8 | ( 8.7 to 11.0 ) |
| 2015 | 4.4 | ( 3.6 to 5.2 ) | 2.3 | ( 1.8 to 2.9 ) | 0.7 | ( 0.4 to 1.0 ) | 10.3 | ( 9.2 to 11.5 ) |
| 2016 | 4.5 | ( 3.8 to 5.4 ) | 3.8 | ( 3.2 to 4.6 ) | 0.8 | ( 0.6 to 1.2 ) | 9.0 | ( 8.0 to 10.2 ) |
| 2017 | 4.1 | ( 3.4 to 4.9 ) | 4.4 | ( 3.7 to 5.2 ) | 0.6 | ( 0.4 to 0.9 ) | 10.5 | ( 9.4 to 11.8 ) |
| 2018 | 4.0 | ( 3.3 to 4.8 ) | 5.0 | ( 4.2 to 5.9 ) | 0.7 | ( 0.4 to 1 ) | 9.5 | ( 8.4 to 10.6 ) |
| 2019 | 4.7 | ( 3.9 to 5.5 ) | 5.4 | ( 4.6 to 6.3 ) | 0.9 | ( 0.6 to 1.3 ) | 9.8 | ( 8.7 to 11.0 ) |
| 2020 | 5.0 | ( 4.2 to 5.9 ) | 4.8 | ( 4.1 to 5.7 ) | 1.0 | ( 0.7 to 1.4 ) | 7.7 | ( 6.7 to 8.7 ) |
| 2021 | 4.4 | ( 3.7 to 5.2 ) | 7.0 | ( 6.1 to 8.0 ) | 1.2 | ( 0.8 to 1.6 ) | 7.8 | ( 6.8 to 8.9 ) |
| 2022 | 5.6 | ( 4.8 to 6.6 ) | 8.9 | ( 7.9 to 10.1 ) | 1.0 | ( 0.7 to 1.4 ) | 5.2 | ( 4.4 to 6.1 ) |

Abbreviation: STD: standardized; CI: confidence interval

Supplemental Table 5-1 Comorbidities associated with liver disease by stages (2004 to 2022)

| Comorbidities | Stage 1, N = 94,529^1^ | Stage 2, N = 4,562^1^ | Stage 3, N = 3,040^1^ | Stage 4, N = 3,221^1^ | Stage 5, N = 2,233^1^ |
| --- | --- | --- | --- | --- | --- |
| CVD related conditions | 7,018(7.4%) | 587(12.9%) | 310(10.2%) | 457(14.2%) | 264(11.8%) |
| Diabetes | 6,135(6.5%) | 612(13.4%) | 303(10.0%) | 234(7.3%) | 200(9.0%) |
| Hypertension/anti-hypertensive | 34,022(36.0%) | 1,939(42.5%) | 1,165(38.3%) | 1,232(38.2%) | 1,006(45.1%) |
| ^1^ n(%) | | | | | |

Supplemental Table 5-2 Comorbidities associated with liver disease by aetiologies (2004-2022)

| Comorbidities | ArLD, N = 19,760^1^ | NAFLD, N = 33,655^1^ | Metablic liver disease, N = 5,469^1^ | HBV, N = 1,063^1^ | HCV, N = 3,539^1^ | Autoimmune liver disease, N = 13,582^1^ | Haemochromatosis, N = 4,111^1^ | Hepatitis not specified, N = 4,783^1^ | Congestive hepatopathy, N = 574^1^ | Toxic liver disease, N = 757^1^ | Miscellaneous, N = 8,426^1^ |
| --- | --- | --- | --- | --- | --- | --- | --- | --- | --- | --- | --- |
| CVD related conditions | 1,442(7.3%) | 1,837(5.5%) | 578(10.9%) | 54(5.2%) | 177(5.5%) | 1,159(8.6%) | 227(5.6%) | 466(9.7%) | 135(23.6%) | 58(7.7%) | 885(10.5%) |
| Diabetes | 1,185(6.0%) | 2,776(8.4%) | 380(7.2%) | 46(4.4%) | 132(4.1%) | 752(5.6%) | 175(4.3%) | 368(7.7%) | 42(7.3%) | 26(3.4%) | 253(3.0%) |
| Hypertension/antihypertensives | 6,911(35.0%) | 12,455(37.5%) | 1,916(36.1%) | 197(19.1%) | 632(19.5%) | 4,670(34.8%) | 1,422(35.2%) | 1,875(39.2%) | 273(47.6%) | 203(26.9%) | 3,468(41.2%) |
| ^1^ n(%) |  |  |  |  |  |  |  |  |  |  |  |
