# Supplementary material for: Incidence rate and associated patient characteristics of liver disease in Wales 2004–2022: a retrospective population-scale observational study
Source: BMJ Open. 2025 Feb 10;15(2):e093335. doi: 10.1136/bmjopen-2024-093335 (PMC11815464; doi:10.1136/bmjopen-2024-093335)
Supplement: Supplementary file 2 [file bmjopen-2024-093335.reviewer_comments.pdf]

PEER REVIEW HISTORY

BMJ Open publishes all reviews undertaken for accepted manuscripts. Reviewers are asked to complete a checklist review form and are provided with free text boxes to elaborate on their assessment. These free text comments are reproduced below.

ARTICLE DETAILS

Title (Provisional)

Incidence rate and associated patient characteristics of liver disease in Wales 2004-2022: a retrospective population-scale observational study

Authors

Gao, Jingwei; Akbari, Ashley; Ahmed, Haroon; Davies, Aled; Yeoman, Andrew; Pembroke , Thomas Peter Ignatius

VERSION 1 - REVIEW

**Reviewer**	**1**
**Name**	**Jepsen, P**
**Affiliation**	**Aarhus University**
**Date**	**01-Oct-2024**
**COI**	**None**

Gao and colleagues present their study of the incidence of liver disease in Wales. It is an interesting topic, but I found the paper unexpectedly difficult to read, and I would have liked to see more epidemiologic data. Specific comments follow:

* The title says "epidemiology", but "incidence" would have been more appropriate, I believe.

* I was thrown off by the repeated segmentation into three different source registries, i.e., WLGP, PEDW, and ADDE. As an international reader, I am not interested in which register identifies which patients - I strongly suggest that you limit the study period and population to what is covered by the SAIL Databank. If you do so, you do not have to describe your study population by source registry, and you can instead focus on more clinically relevant aspects. For example, I do find it interesting whether patients were diagnosed with liver disease whilst they were still alive, but the Results section should not present results by source register.

* The main message is that the incidence has increased because patients are identified earlier, likely owing to more screening in the at-risk populations. I thought it would be obvious to study time-trends in survival and stage-specific survival to examine whether

screening leads to better outcomes or just more patients. As it is, your final sentence about "It is therefore crucial to focus more attention on early detection, ..." appears unfounded.

* What is the point of looking for comorbidities in different time windows prior to diagnosis of liver disease? As a reader, I am interested to know whether patients had or did not have a particular comorbid disease. You might choose to examine different definitions in a sensitivity analysis, but in that case you should label it as a sensitivity analysis and explain your reasons for doing it.

* Changes in coding practice may have affected your findings and should be discussed. This is particularly true if hospitals have had changing (financial) incentives during the study period.

* I take some issue with the staging of liver disease. For example, labeling primary liver cancer as 'stage 5' implies that patients went through stages 1-4 first, but that may not be true. Also, codes for ascites (and ascites puncture/drainage) may not be reliable as a way to identify patients with liver disease, but among patients with liver disease it is a very sensitive way to identify those with portal hypertension. Please explain or amend your definition with respect to ascites. (I do not object to your decision to stage liver disease.)

* You should include your definitions of comorbidities in a Supplementary Appendix. I could not access the Github list.

* The stacked bar charts make it difficult to assess trends in the "middle stages", i.e., stages 2-4 in Figure 3 and all but 'ArLD' and 'Miscelleaneous' in Figure 4. Please consider presenting the rates as line charts, too.

* In the footnote to Supplemental Table 1, I believe you mean "comply" instead of "complain".

**Reviewer**	**2**
**Name**	**Zhang, Tiejun**
**Affiliation**	**Fudan University School of Public Health, Epidemiology**
**Date**	**17-Oct-2024**
**COI**	**None**

The present study has detailed a study on the incidence of liver disease in Wales from 2004 to 2022, utilizing data from the SAIL Databank. It revealed a significant increase in liver disease incidence over the period, driven primarily by a rise in non-alcoholic fatty liver disease (NAFLD). The research highlights the growing importance of primary care in early detection and the high prevalence of comorbidities such as hypertension, diabetes, and cardiovascular diseases among liver disease patients. Overall, the manuscript presents

valuable insights into the changing landscape of liver disease incidence in Wales. I have some suggestions for the authors.

1. The manuscript should discuss the potential impact of changes in diagnostic practices, coding, and healthcare-seeking behavior over the study period, as these factors could influence the observed trends.

2. The electronic health records for primary and secondary care may miss cases of liver disease that are managed outside of these systems, potentially leading to an underestimation of the true incidence.

3.Also the reliance on ICD-10 coding for identifying liver disease cases might lead to underdiagnosis or misdiagnosis, particularly if there have been changes in coding practices or if primary care providers vary in their use of specific codes.

VERSION 1 - AUTHOR RESPONSE

Response to Reviewer: 1

Thank you for your review of our paper. We have answered each of your points below.

1. The title says "epidemiology", but "incidence" would have been more appropriate, I believe.

Thank you for this helpful suggestion. We agree that "incidence" more accurately reflects the focus of our study. In response, we have revised the title to **Incidence rate and associated patient characteristics of liver disease in Wales 2004-2022: a retrospective population-scale observational study** to ensure precision and clarity.

2. I was thrown off by the repeated segmentation into three different source registries, i.e., WLGP, PEDW, and ADDE. As an international reader, I am not interested in which register identifies which patients - I strongly suggest that you limit the study period and population to what is covered by the SAIL Databank. If you do so, you do not have to describe your study population by source registry, and you can instead focus on more clinically relevant aspects. For example, I do find it interesting whether patients were diagnosed with liver disease whilst they were still alive, but the Results section should not present results by source register.

Thank you for your thoughtful feedback on our study's data presentation. In response, we have revised the grouping of data to reflect primary care, secondary care, mortality, and multi-source group, rather than segmenting by specific registries (WLGP, PEDW, and ADDE). This restructuring simplifies the study's presentation and enhances its accessibility for international readers.

We would also like to emphasize that a key strength and novelty of our work lies in the integration of these data sources, which allows us to compare patient characteristics across different clinical settings. By combining primary, secondary, and mortality data, we provide a more comprehensive perspective on liver disease that captures variations across healthcare

contexts. This integrated approach not only deepens our understanding of the clinical and socio-demographic factors associated with liver disease but also offers insights into different pathways and stages of disease management.

Thank you again for your valuable suggestions, which have enabled us to enhance the clarity and relevance of our findings.

3. The main message is that the incidence has increased because patients are identified earlier, likely owing to more screening in the at-risk populations. I thought it would be obvious to study time-trends in survival and stage-specific survival to examine whether screening leads to better outcomes or just more patients. As it is, your final sentence about "It is therefore crucial to focus more attention on early detection, ..." appears unfounded.

Thank you for your insightful feedback on our discussion. In line with your suggestions, we have revised the discussion section to more accurately reflect the current understanding of early detection's impact on liver disease outcomes. We have removed the final sentence calling for more attention on early detection, ensuring that the discussion remains balanced and evidence-based.

As international guidelines propose, early detection may indeed lead to improved long-term outcomes. However, we agree that specific studies are needed to directly demonstrate these effects and to assess whether increased screening efforts lead to better outcomes or simply identify more cases. Addressing this question is an important aspect of our future work, where we aim to study patient outcomes associated with early-stage screening.

4. What is the point of looking for comorbidities in different time windows prior to diagnosis of liver disease? As a reader, I am interested to know whether patients had or did not have a particular comorbid disease. You might choose to examine different definitions in a sensitivity analysis, but in that case you should label it as a sensitivity analysis and explain your reasons for doing it.

Thank you for your thoughtful feedback regarding the presentation of comorbidity data. In response, we have revised this section to present comorbidity frequencies based on data sources, disease stages, and aetiologies, rather than examining time windows prior to diagnosis. This approach aligns more directly with your suggestion, providing a clearer view of whether patients had or did not have specific comorbidities at the time of their liver disease diagnosis.

5. Changes in coding practice may have affected your findings and should be discussed. This is particularly true if hospitals have had changing (financial) incentives during the study period.

Thank you for raising this point. In Wales, there have been no changes to coding practices over the study period, as health board allocations are based on criteria outlined in Welsh government policy rather than shifting financial incentives. We consider this a strength of our research, as it helps to minimize potential biases related to coding changes and provides a reliable, objective reflection of disease incidence and management trends. We have clarified

this point in the discussion, referencing the Welsh government document on health board and public health allocations (2021-22 health board and public health Wales NHS trust allocations), which outlines the allocation criteria and emphasizes the consistency in practices.

6. I take some issue with the staging of liver disease. For example, labeling primary liver cancer as 'stage 5' implies that patients went through stages 1-4 first, but that may not be true. Also, codes for ascites (and ascites puncture/drainage) may not be reliable as a way to identify patients with liver disease, but among patients with liver disease it is a very sensitive way to identify those with portal hypertension. Please explain or amend your definition with respect to ascites. (I do not object to your decision to stage liver disease.)

Thank you for your thoughtful feedback on our staging criteria. In response:

- We have excluded ascites as a marker of advanced liver disease because it could be caused by other conditions, such as malignancy, cardiac, and renal diseases, which could confound its association with liver disease specifically. Ascites is generally recognized as a late sign of liver disease decompensation rather than solely an indicator of portal hypertension, so we have chosen not to group it with portal hypertension without decompensation.
- We acknowledge the possibility of developing hepatocellular carcinoma (HCC) without prior cirrhosis. However, given that over 90% of HCC cases in Wales are associated with cirrhosis, we have classified HCC as a late stage in our staging model. This classification reflects both the high mortality associated with HCC and the elevated risk of decompensation.

We hope these clarifications address your concerns and enhance the clarity of our staging approach.

7. You should include your definitions of comorbidities in a Supplementary Appendix. I could not access the Github list.
Thank you for your suggestion and for highlighting the access issue. We apologize for any inconvenience caused by the GitHub link. In response, we have now included the definitions of comorbidities in a Supplementary Appendix to ensure accessibility for all readers.

8. The stacked bar charts make it difficult to assess trends in the "middle stages", i.e., stages 2-4 in Figure 3 and all but 'ArLD' and 'Miscelleaneous' in Figure 4. Please consider presenting the rates as line charts, too.

Thank you for your feedback on the clarity of trend presentation in the stacked bar charts. In response, we have added line charts alongside the bar charts in Figures 2-4. This addition provides a clearer view of trends for incidence rates for all data sources, stages and aetiologies, allowing for easier comparison and interpretation.

9. In the footnote to Supplemental Table 1, I believe you mean "comply" instead of "complain".

Thank you for catching this error. We apologize for the oversight and have corrected "complain" to "comply" in the footnote of Supplemental Table 1.

Response to Reviewer: 2

Thank you for your review of our paper. We have answered each of your points below.

1. The manuscript should discuss the potential impact of changes in diagnostic practices, coding, and healthcare-seeking behavior over the study period, as these factors could influence the observed trends.

Thank you for your insightful comments. We agree that changes in diagnostic practices, coding, and healthcare-seeking behavior over the study period are important factors that could influence the observed trends in our data. We have addressed this in the limitations section, acknowledging that changes in healthcare-seeking behavior may influence our findings. However, there have been no changes to coding practices over the study period, as health board allocations are based on criteria outlined in Welsh government policy rather than shifting financial incentives and we consider it a strength of our study. We have clarified this point in the discussion, referencing the Welsh government document on health board and public health allocations (2021-22 health board and public health Wales NHS trust allocations), which outlines the allocation criteria and emphasizes the consistency in practices.

2. The electronic health records for primary and secondary care may miss cases of liver disease that are managed outside of these systems, potentially leading to an underestimation of the true incidence.

Thank you for pointing out the limitations inherent in using electronic health records (EHR) for primary and secondary care. We acknowledge that our reliance on these systems may not capture cases of liver disease managed outside of these settings, potentially leading to an underestimation of the true incidence. Particularly, our study exclusively included individuals with a history of GP registration and residency information in Wales, which means we may have missed cases among individuals unable to register with a GP—an at-risk subpopulation for liver disease.

Despite these limitations, we have made a concerted effort to mitigate this issue by combining data from primary, secondary, and mortality records to comprehensively capture all possible cases across Wales. This will also cover paper-based notes as they are captured and coded within NHS Wales, and the universal primary care structure includes all healthcare providers, including private practice. This integration aims to provide the most inclusive representation of liver disease incidence within the constraints of available data sources. We believe that this approach significantly strengthens our study and enhances the robustness of our findings.

3.Also the reliance on ICD-10 coding for identifying liver disease cases might lead to underdiagnosis or misdiagnosis, particularly if there have been changes in coding practices or if primary care providers vary in their use of specific codes.

Thank you for raising concerns regarding the reliance on ICD-10 coding for identifying liver disease cases in our study. Indeed, the potential for underdiagnosis or misdiagnosis due to variations in coding practices or inconsistencies in code usage by primary care providers is an important consideration. However, as we discussed in previous response, coding practice in Wales has not changed in response to funding incentives. This stability in coding practice helps mitigate some of the potential biases and misdiagnoses associated with changes in diagnostic criteria or coding habits over time.

For further details on the funding structures and their lack of direct association with coding practices in Wales, readers are referred to the governmental publication on health allocations: Welsh Government Health Board and Public Health Wales NHS Trust Allocations 2021-22. This source provides additional context on how our data collection methods are shielded from some of the financial pressures that might otherwise affect diagnostic coding in healthcare.

VERSION 2 - REVIEW

**Reviewer**	**1**
**Name**	**Jepsen, P**
**Affiliation**	**Aarhus University**
**Date**	**14-Dec-2024**
**COI**	

Thank you for revising your manuscript. I think it is better now, but I still have a few points that I would like you to clarify:

* Page 7: You write that "We defined the time of the first chronic or acute liver disease diagnosis as the index date." This is irrespective of WHERE you identified the patient, right? I assume that a patient cannot be included more than once, but please clarify this for the readers.

* On page 6, you write that patients were followed until "GP de-registration". Please describe in greater detail what this means. Specifically, what happened if a patient moved from one SAIL-contributing GP to another?

* On page 8, you write that "The incidence rate was calculated as the number of incident cases per Welsh residents", but on page 6 you write that "We excluded individuals not registered with a SAIL-contributing general practice (GP)". If these people are not in the numerator of the incidence calculation, I would think that they should be excluded from the

denominator, too. Can you compute the incidence rate of liver disease in the SAIL-contributing GP practice population?

* Following from the previous comment, can the increasing incidence rate of liver disease in the primary care group be explained by an increasing number of SAIL-contributing GP practices? This could be in addition to the explanation you provide on page 12 ("an increasing detection rate of liver disease in primary care").

**Reviewer**	**2**
**Name**	**Zhang, Tiejun**
**Affiliation**	**Fudan University School of Public Health, Epidemiology**
**Date**	**08-Dec-2024**
**COI**	

The revisions are fine. I have no further comments.

VERSION 2 - AUTHOR RESPONSE

Reviewer: 2
Dr. Tiejun Zhang, Fudan University School of Public Health
Comments to the Author:
The revisions are fine. I have no further comments.

Thank you for reviewing the revisions to our manuscript and for your comments confirming their adequacy. We appreciate your time and the thoughtful attention you have given to our work.

We are pleased to hear that no further revisions are required and are grateful for your contribution to refining our manuscript.

Thank you once again for your valuable insights and support throughout the review process.

Reviewer: 1
Dr. P Jepsen, Aarhus University
Comments to the Author:
Thank you for revising your manuscript. I think it is better now, but I still have a few points that I would like you to clarify:

* Page 7: You write that "We defined the time of the first chronic or acute liver disease diagnosis as the index date." This is irrespective of WHERE you identified the patient, right? I assume that a patient cannot be included more than once, but please clarify this for the readers.

Thank you for your insightful observation regarding the clarity needed in defining the index date for liver disease diagnosis. In response to your comment, we clarified on page 6 that

“The first liver disease diagnosis across all data sources is considered to be the index liver disease event” and page 7: “we defined the time of the first chronic or acute liver disease diagnosis across all data sources as the index date”.

I believe this clarification would confirm that each patient is included only once in our analysis, irrespective of where or how many times the diagnosis has been recorded.

* On page 6, you write that patients were followed until "GP de-registration". Please describe in greater detail what this means. Specifically, what happened if a patient moved from one SAIL-contributing GP to another?

Thank you for prompting a more detailed description of what constitutes GP de-registration in our study and its implications for patient follow-up. In response to your valuable feedback, we have revised the manuscript to include a clear definition and a comprehensive explanation of our follow-up methodology, please see page 6: "GP de-registration is defined as the termination of an individual’s registration with a SAIL-contributing GP, as indicated by the last end date in the registration records”. As we required GP registration for individuals identified from primary care, this is applicable only to those whose index event was identified from WLGP data source.

Furthermore, we’d like to clarify that we tracked each patient’s GP registration history, including all start and end dates at different GP practices. This detailed tracking allows us to manage and maintain the continuity of GP registration, even when patients switch between different SAIL-contributing GPs. We address potential gaps in registration—short gaps of less than 30 days are automatically closed by SAIL, as short gaps do not indicate a permanent relocation out of Wales, thus ensuring uninterrupted follow-up.

In some rare situations, patients may experience longer gaps between different GP registrations. We continued to follow these patients provided the last end date of their registration falls within the study period, thereby capturing all relevant and eligible health events that occur within this timeframe. It is important to note, however, that any gaps in GP registration will specifically exclude WLGP records from the analysis during those periods where GP registration is lacking. This exclusion is necessary to maintain the accuracy and reliability of the data, as GP registration is a prerequisite for validating the eligibility of health events recorded in the WLGP data source.

We hope this clarifies the methodologies and address your concerns fully and clarify the processes we use to maintain the continuity and accuracy of our patient tracking.

* On page 8, you write that "The incidence rate was calculated as the number of incident cases per Welsh residents", but on page 6 you write that "We excluded individuals not registered with a SAIL-contributing general practice (GP)". If these people are not in the numerator of the incidence calculation, I would think that they should be excluded from the denominator, too. Can you compute the incidence rate of liver disease in the SAIL-contributing GP practice population?

Thank you for your insightful comment regarding the calculation of the incidence rate of liver disease within our study.

In our manuscript, we aimed to provide a comprehensive analysis of the changes in the incidence rate across the entire Welsh population. To achieve this, we included data from individuals in primary care, secondary care, and mortality records. We required all

individuals to be Welsh residents to ensure accuracy in capturing the target population. GP registration was mandated only for those individuals whose index event was identified from the primary care data source. As noted on page 6: "Individuals were required to be residents in Wales at the time of cohort entry. GP registration was required if the index event was from WLGP." Therefore, we chose to use the Mid-year population estimates for all Welsh residents as the denominator to reflect the individuals captured outside primary care data, which represent over 60% of the full cohort.

Furthermore, SAIL represents approximately 80% of Welsh GP practices, providing effective coverage and robust data for our eligible cohort. However, we acknowledge your concern regarding the potential exclusion of individuals not registered with a SAIL-contributing GP. This exclusion might lead to an underestimation of the incidence rate based on primary care data alone. We have addressed this limitation in our manuscript, ensuring that our study remains transparent and reflective of its methodological scope. Please see page 13: The exclusion may also introduced a potential underestimation of the true incidence rate of liver disease in the primary care data.

As requested, we'd like to provide results of additional analysis where we recalculated the crude incidence rate using only the population registered at SAIL primary care general practices as the denominator in this response letter. The trends observed in this adjusted analysis closely mirrored those of our original findings, indicating the choice of denominator does not influence our primary finding (Table 1, Figure 1).

* Following from the previous comment, can the increasing incidence rate of liver disease in the primary care group be explained by an increasing number of SAIL-contributing GP practices? This could be in addition to the explanation you provide on page 12 ("an increasing detection rate of liver disease in primary care").

Thank you for your query about the potential effects of the increasing number of SAIL-contributing GP practices on the observed incidence rates of liver disease in the primary care group.

Based on our internal reports, we acknowledge that the number of SAIL-contributing GP practices has indeed changed annually (https://docs.hiru.swan.ac.uk/display/SATP/WLGP+coverage+reports). There has been a small increase in the total number of individuals registered at SAIL primary care practices, and our analyses suggest that this minimal increase is unlikely to influence the results due to its limited scale.

In addition, the additional data we provided in response to question 3 demonstrated similar trends observed to our original findings, indicating that the increase in GP practices and registered individuals does not substantively affect the overall trend of increasing liver disease incidence rates we reported (Table 1, Figure 1).

Table 1. Crude incidence rate of chronic liver disease using total population and GP registered population as denominator: 2004-2022

Year	Wales population	GP registered population	Incident cases	Crude incidence (95% CI)	GP registered incidence (95% CI)
**2004**	3,131,640	2,419,036	3,060	97.7 (94.3 - 101.2)	126.5 (122.1 - 131.1)
**2005**	3,105,633	2,467,327	3,403	109.6 (105.9 - 113.3)	137.9 (133.3 - 142.6)
**2006**	3,104,483	2,487,920	3,670	118.2 (114.4 - 122.1)	147.5 (142.8 - 152.4)
**2007**	3,087,732	2,535,695	3,537	114.6 (110.8 - 118.4)	139.5 (134.9 - 144.2)
**2008**	3,083,840	2,557,993	3,867	125.4 (121.5 - 129.4)	151.2 (146.4 - 156.0)
**2009**	3,081,366	2,568,238	4,008	130.1 (126.1 - 134.2)	156.1 (151.3 - 161,0)
**2010**	3,077,165	2,578,611	4,268	138.7 (134.6 - 142.9)	165.5 (160.6 - 170.6)
**2011**	3,072,739	2,593,787	4,519	147.1 (142.8 - 151.4)	174.2 (169.2 - 179.4)
**2012**	3,073,788	2,612,223	4,546	147.9 (143.6 - 152.3)	174.0 (169.0 - 179.2)
**2013**	3,071,058	2,626,951	4,733	154.1 (149.8 - 158.6)	180.2 (175.1 - 185.4)
**2014**	3,070,928	2,644,394	5,145	167.5 (163.0 - 172.2)	194.6 (189.3 - 200.0)
**2015**	3,063,758	2,666,465	5,659	184.7 (179.9 - 189.6)	212.2 (206.7 - 217.8)
**2016**	3,049,971	2,711,448	6,548	214.7 (209.5 - 220.0)	241.5 (235.7 - 247.4)
**2017**	3,038,872	2,727,719	6,981	229.7 (224.4 - 235.2)	255.9 (250.0 – 262.0)
**2018**	3,025,867	2,741,466	7,953	262.8 (257.1 - 268.7)	290.1 (283.8 - 296.5)
**2019**	3,006,299	2,731,439	8,481	282.1 (276.1 - 288.2)	310.5 (303.9 - 317.2)
**2020**	2,985,668	2,740,753	7,327	245.4 (239.8 - 251.1)	267.3 (261.2 - 273.5)
**2021**	2,969,309	2,751,083	9,136	307.7 (301.4 - 314.1)	332.1 (325.3 - 339.0)
**2022**	2,957,422	2,766,919	8,596	290.7 (284.5 - 296.9)	310.7 (304.1 - 317.3)

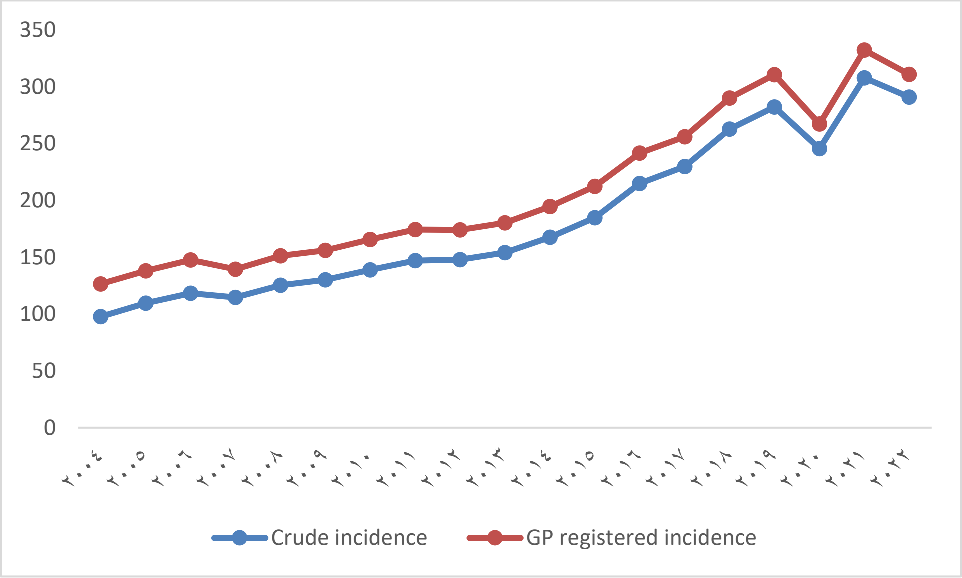

Figure 1. Crude incidence rate of chronic liver disease using total population and GP registered population as denominator: 2004-2022
