# Supplementary material for: Correction: Incidence rate and associated patient characteristics of liver disease in Wales 2004–2022: a retrospective population-scale observational study
Source: BMJ Open. 2025 Sep 3;15(9):e093335corr1. doi: 10.1136/bmjopen-2024-093335corr1 (PMC12410657; doi:10.1136/bmjopen-2024-093335corr1)
Supplement: online supplemental file 1 [file bmjopen-15-9-s001.pdf]

Supplemental Materials

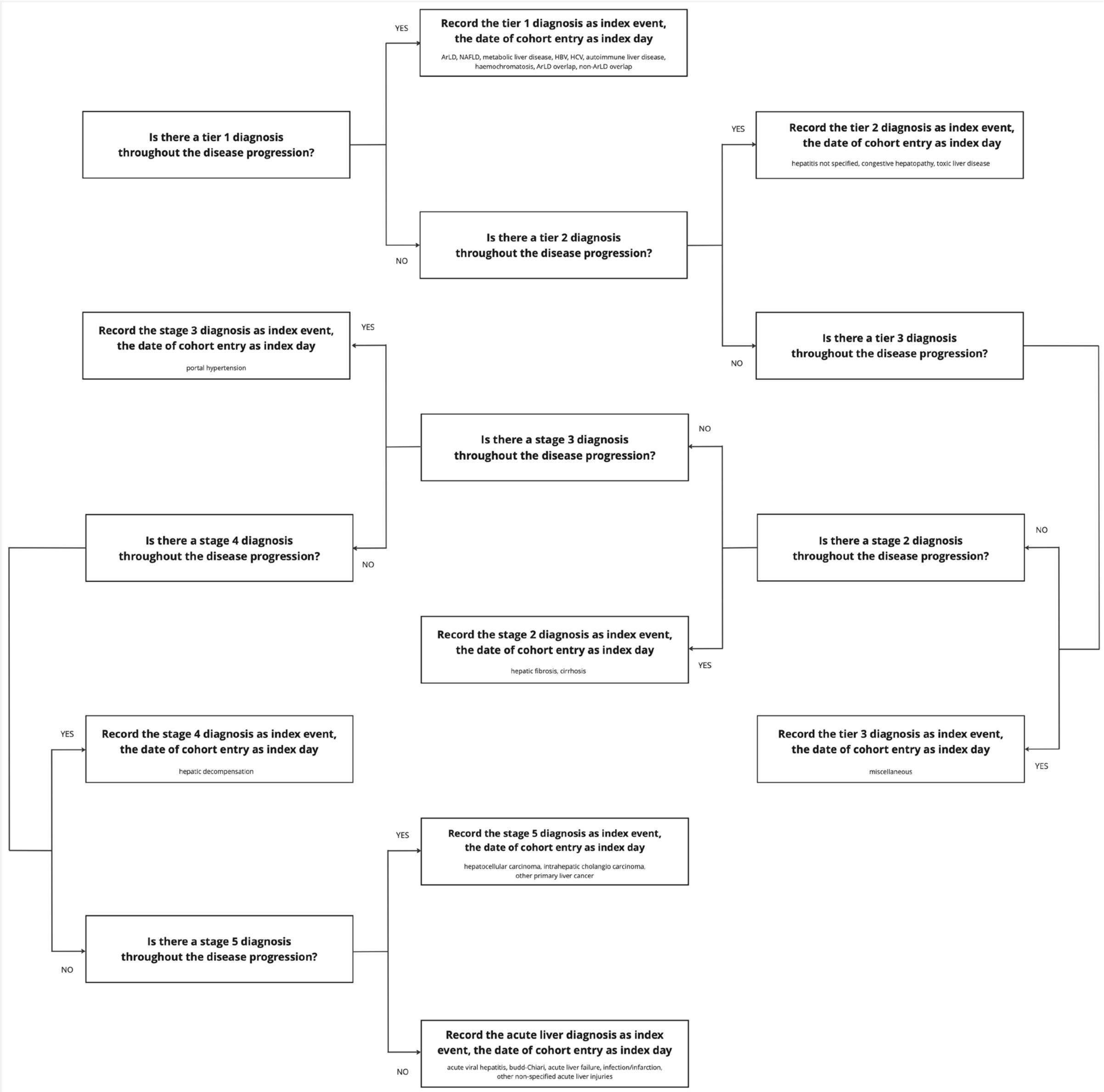

Supplemental Fig. 1 Flowchart for Identifying Index Events and Determining Cohort Entry Date.

This flowchart illustrates the decision rules for identifying the index event and determining the cohort entry date for study participants. The index date is defined as the date of the first diagnosis between 2004 and 2022. The aetiological diagnoses throughout the disease progression were identified as the index event entering the cohort. If no aetiological diagnosis is present, the index event is defined based on the following sequence: stage 2 diagnoses, stage 3 diagnoses, stage 4 diagnoses, and stage 5 diagnoses.

The decision flow proceeds as follows:

Tier 1 Diagnosis: If present at any point during the disease progression, the tier 1 diagnosis is recorded as the index event, and the date of the cohort entry is the index day. Tier 1 diagnoses include ArLD, NAFLD, metabolic liver disease, HBV, HCV, autoimmune liver disease, and haemochromatosis.

Tier 2 Diagnosis: If there is no tier 1 diagnosis, the presence of a tier 2 diagnosis is checked. If present, it is recorded as the index event, and the date of cohort entry is the index day. Tier 2 diagnoses include hepatitis not specified, congestive hepatopathy, and toxic liver disease.

Tier 3 Diagnosis: In the absence of tier 1 and tier 2 diagnoses, the presence of a tier 3 diagnosis is checked. If found, it is recorded as the index event. Tier 3 diagnoses include miscellaneous conditions.

Stage 2 Diagnosis: If none of the Stage 1 diagnoses are present, the presence of a stage 2 diagnosis is considered. If found, it is recorded as the index event. Stage 2 diagnoses include hepatic fibrosis and cirrhosis.

Stage 3 Diagnosis: If no stage 1 or stage 2 diagnoses are present, a stage 3 diagnosis is checked next. If found, it is recorded as the index event. Stage 3 diagnoses include portal hypertension.

Stage 4 Diagnosis: If the stage 1, stage 2, and stage 3 diagnoses are absent, a stage 4 diagnosis is checked. If present, it is recorded as the index event. Stage 4 diagnoses include hepatic decompensation.

Stage 5 Diagnosis: If no above diagnoses are found, a stage 5 diagnosis is considered. If found, it is recorded as the index event. Stage 5 diagnoses include hepatocellular carcinoma, intrahepatic cholangiocarcinoma, other primary liver cancer.

Acute Liver Diagnosis: If none of the chronic diagnoses are present, an acute liver diagnosis is checked. If found, it is recorded as the index event. Acute liver diagnoses include acute viral hepatitis, Budd-Chiari, acute liver failure, infections/sepsis, and other non-specified acute liver injuries.

Supplemental Tables

Supplemental Table 1 Code list for identifying liver disease

Phenotype	ICD10 codes	Read codes
**Acute viral hepatitis**	B15, B19, B16, B17 (B171 excluded), B172, B178, B179, B180, B159, B169, B199	A70z1, AyuB0, XE2u., Q4090, A700., A701., A7052, A70.., A706., A708., A709., A70z., A70G., A704., AyuB3
**Acute liver failure**	K720	J6000
**Budd-Chiari**	I820	G820.
**Infection/infarction**	K750, K763, K751	J62.., J620., J6200, J6201, J6202, J6203, J6204, J620z, A053., J634., J621.
**Other non-specified acute liver injuries**	K752	J63y1
**Autoimmune liver disease**	K754, K743, K831, K753	J63B., J6141, J6160, J6617, J63X.
**Haemochromatosis**	E831	C3500
**Metabolic liver disease**	E880, E830	C3762, C3761, C3510
**HBV** *	B181, B180	
**HCV** *	B182	A70E., A70F.
**Alcohol-related liver disease**	K70	J613., J6130, J612., J6120, J610., J617., J6170, J611.
**Non-alcoholic fatty liver disease**	K760, K7581	J61y1, J61y8
**Hepatitis not specified**	K769, K7589, K73	Jyu72, J614y
**Congestive hepatopathy**	K761, K762, K765	J630., J636., J637.
**Toxic liver disease**	K71	J635., J6350, J6351, J63252, J6353, J6354, J6355, J6356, J6357, J635X
**Miscellaneous**	K764, K768, K77	J638., Jyu73, J63yz, Jyu75
**Hepatic fibrosis**	K740, K741, K742	J61y4, J61y6, J61y5
**Cirrhosis**	K703, K744, K745, K746, K749	J6161, J616z, J615z
**Portal hypertension**	K766, I81, I859, I982, I85	J623., G81.., G8523, G852., G8521, G8522, G852z
**Hepatic decompensation**	K721, K767, I850, K72, C220	J624., SP143, G850., G8520, J625., B1503, BB5D7,

		BB5D5, BB5D8
**Hepatocellular carcinoma (HCC)**	C220	B1503, BB5D7, BB5D5, BB5D8
**Intrahepatic cholangio carcinoma (ICC)**	C221	B150.
**Other primary liver cancers**	C222, C223, C224, C225, C226. C227	B808.

*We identified Read codes(A7071, A7073, ZV02B, Q4091, 43B4., A7070, A7051, A7072, A70z0, A70A., A70B., A70C., A70D., A70E.,A70F., ZV02C) and ICD-10 codes (B180, B181, B182) for HBV and HCV. However, in order to comply with Data Protection Act 2018 and the UK General Data Protection Regulation, we could not include Read codes (A7071, A7073, ZV02B, Q4091, 43B4., A7070, A7051, A7072, A70z0, A70A., A70B., A70C., A70D., ZV02C) and ICD-10 codes (B171) as these were flagged as sensitive in the latest version of known sensitive code list of SAIL Databank.

Supplemental Table 2-1 ICD-10 code list for identifying comorbidities

Comorbidities	ICD-10 codes
**Atrial fibrillation**	I481, I482
**Angina**	I200, I201, I208, I209
**Asthma**	J45
**Diabetes**	E09, E10, E102, E103, E11, E13, K86
**Heart failure**	I50, I501, I502, I503, I508
**Hypertension**	I10, I11, I12, I13, I15
**Peripheral vascular disease**	E106, E116, I70
**Renal disease**	N18
**Stroke**	I691, I61, I63, I64, I60, I66, G45.0, G45.1, G45.3, G46.0, G46.2, G45.8, I65, G46.1, 45.9, G45.2, G45.4
**Transient ischaemic attack**	I61, I63, I66
**Other ischaemic**	I20, I21, I22, I23, I24, I25

Supplemental Table 2-2 Read codes list for identifying comorbidities

Comorbidities	Read codes
**Atrial fibrillation**	14AN., 14AR., 3272, 3273, 8CMW2, G573., G5730, G5731, G5732, G5733, G5734, G5735, G5736, G5737, G5738, G5739, G573z, 3272, 3273, 793M1, 793M3
**Angina**	G3112, G33.., G330., G3300, G330z, G33z., G33z3, G33z7, G33zz, 662K., 662K0, 662K1, 662K2, 662Kz, 8B27., G33z1, G33z2, G33z5, G33z6, G34y0, Gyu30
**Asthma**	H33.., H330., H3300, H3301, H330z, H331., H3310, H3311, H331z, H332., H333., H334., H335., H33z., H33z0, H33z1, H33z2, H33zz,
**Diabetes**	66AJ., 66AJ1, 66AJz, 66An., 66Ao., 8CR2., 9OLA., 9OLA., C10.., C100., C1000, C100z, C101., C1010, C1011, C101y, C102., C1020, C1021, C102z, C103., C1030, C1031, C103y, C103z, C104., C1040, C1041, C104y, C104z, C105., C1050, C1051, C105y, C105z, C106., C1060, C1061, C106y, C106z, C107., C1070, C1071, C1072, C1073, C1074, C107y, C107z, C108., C1080, C1081, C1082, C1083, C1084, C1085, C1086, C1087, C1088, C1089, C108A, C108B, C108C, C108D, C108E, C108F, C108G, C108H, C108J, C108y, C108z, C109., C1090, C1091, C1092, C1093, C1094, C1095, C1096, C1097, C1099, C109A, C109B, C109C, C109D, C109E, C109F, C109G, C109H, C109J, C109K, C10A., C10A0, C10A1, C10A2, C10A3, C10A4, C10A5, C10A6, C10A7, C10AW, C10AX, C10B., C10B0, C10C., C10D., C10E., C10E0, C10E1, C10E2, C10E3, C10E4, C10E5, C10E6, C10E7, C10E8, C10E9, C10EA, C10EB, C10EC, C10ED, C10EE, C10EF, C10EG, C10EH, C10EJ, C10EK, C10EL, C10EM, C10EN, C10EP, C10EP, C10EQ, C10ER, C10F., C10F0, C10F1 C10F2, C10F3, C10F4, C10F5, C10F6, C10F7, C10F9, C10FA, C10FB, C10FC, C10FD, C10FE, C10FF, C10FG, C10FH, C10FJ, C10FK, C10FL, C10FM, C10FN, C10FP, C10FQ, C10FR, C10FS, C10G., C10G0, C10H., C10H0, C10K., C10K0, C10M., C10M0, C10N., C10N0, C10N1, C10P., C10P0, C10P1, C10y., C10y1, C10yy, C10yz, C10z., C10z0, C10z1, C10zy, C10zz, F372., F3720, F3721, F3722, 1434, 14F4., 14P3., 9OL9.
**Heart failure**	G58.., G580., G5800, G5801, G5802, G5803, G5804, G581., G5810, G582., G583., G584., G58z., G232., G234., G1yz1, 1O1.., 662W., 662p., 8B29., 8H2S., 9Or0., G400., G41z., G5540, G5540, G5yy9, G5yyA, R2y10, 585f., 585g., 14A6., 14AM., 1736, 1J60., 23E1., 388D., 662T., 662f., 662g., 662h., 662i., 679X., 8CL3., 8HBE., 8HHz., 8Hg8., 8Hk0., 9N0k., 9N2p., 9N4s., 9N4w., 9N6T., 9On.., 9On0., 9On1., 9On2., 9On3., 9On4., 9Or.., 9Or1., 9Or2., 9Or3., 9Or4., 9Or5., 9h1.., 9h11., 9h12., 9hH.., 9hH0., 9hH1., G581., H54.., H541., H5410, H541z, H54z., H584., H584z, ZRad.
**Hypertension**	14A2., G2..., G20.., G200., G201., G202., G203., G20z., G21.., G210., G2100, G2101, G210z, G211., G2110, G2111, G211z, G21z., G21z0, G21z1, G21zz, G22.., G220., G221., G222., G22z., G23.., G230., G231., G232., G233., G234., G23z., G24.., G240., G2400, G240z, G241., G2410, G241z, G244., G24z., G24z0, G24z1, G24zz, G25.., G250., G251., G26.., G27.., G28.., G2y.., G2z.., 6627, 6628, 662F., 662G., 662O., 662b., 662c., 662d., 662r., 7Q01., 8B26., 8BL0., 8I3N., F4042, F4213, G672., Gyu2., L122., L1220, L1221, L1223, L122z, L127., L127z, L128., L1280, L1282, Gyu21

Comorbidities	Read codes
**Peripheral vascular disease**	G73.., G734., G73y., G73z., G73z0, G73zz, Gyu74, 2G63., A3A0F, C107., C1070, C1071, C1073, C1074, C107z, C108G, C109F, C109F, C10EG, C10FF, G700., G702., G702z, G731., G7310, G731z, G732., G7320, G7321, G733., G73y0, G73y1, G73yz, G740., G742z, M271., M2710, M2713, R0550, R0550
**Renal disease**	1Z13., 1Z14., 1Z1H., 1Z1J., 1Z1K., 1Z1L., K050., K054., K055., K060., K060., K08z., K0D.., 1Z10., 1Z17., 1Z18., 1Z11., 1Z19., 1Z1A., 1Z12., 1Z15., 1Z16., 1Z1B., 1Z1C., 1Z1D., 1Z1E., 1Z1F., 1Z1G.,
**Stroke**	G6..., G61.., G610., G611., G612., G613., G614., G615., G616., G617., G618., G619., G61X., G61X0, G61X1, G61z., G63.., G630., G631., G632., G633., G634., G63y., G63y0, G63y1, G63z., G64.., G640., G6400, G641., G6410, G64z., G64z0, G64z1, G64z2, G64z3, G64z4, G66.., G660., G661., G662., G663., G664., G665., G666., G667., G668., G67.., G670., G671., G6710, G6711, G671z, G677., G6770, G6771, G6772, G6773, G6774, G679., G67y., G67z., G6y.., G67z.
**Transient ischaemic attack**	G65z., G65zz, G65z1, G65y., 14AB., G65z0, Fyu55, G65.., G65.., G650., G651., G6510, G652., G653., G654., G655., G656., G657., G65y., F4236, 14AB0
**Other ischaemic**	G33z4, G34.., G34y., G34y0, G34y1, G34yz, G34z., G34z0, G3y.., G3z.., G31y3, G332., 6A2.., 6A4.., 8B3k., 8H2V., G3..., G31.., G3110, G31y., G31y2, G31yz, G340., G343., G344., Gyu3., Gyu32, Gyu33
**Anti-hypertensive**	bi1.., bi11., bi12., bi13., bi14., bi15., bi16., bi17., bi18., bi19., bi1A., bi1B., bi1C., bi1H., bi1I., bi1J., bi1K., bi1a., bi1b., bi1c., bi1d., bi1g., bi1h., bi1i., bi1j., bi1k., bi1l., bi1m., bi1n., bi1o., bi1p., bi1q., bi1r., bi1v., bi1w., bi1x., bi1y., bi1z., bi2.., bi21., bi22., bi23., bi24., bi25., bi26., bi27., bi29., bi2A., bi2B., bi2C., bi2D., bi2E., bi2F., bi2G., bi2H., bi2J., bi2K., bi2L., bi2M., bi2a., bi2t., bi2u., bi2v., bi2w., bi2x., bi2y., bi2z., bi3.., bi31., bi32., bi33., bi34., bi35., bi36., bi37., bi38., bi39., bi3a., bi3b., bi3c., bi3c., bi3d., bi3d., bi3e., bi3f., bi3g., bi3h., bi3i., bi3j., bi3k., bi3l., bi3m., bi3q., bi3r., bi3y., bi4.., bi41., bi42., bi43., bi44., bi45., bi46., bi47., bi49., bi4A., bi4B., bi4C., bi4D., bi4E., bi5.., bi51., bi52., bi53., bi54., bi57., bi58., bi6.., bi61., bi62., bi63., bi64., bi65., bi66., bi67., bi68., bi69., bi6A., bi6B., bi6C., bi6D., bi6E., bi6F., bi6G., bi6o., bi6p., bi6q., bi6r., bi6s., bi6t., bi6u., bi6v., bi6w., bi6x., bi6y., bi6z., bi7.., bi71., bi72., bi73., bi74., bi8.., bi81., bi82., bi82., bi83., bi83., bi84., bi84., bi85., bi86., bi86., bi87., bi87., bi88., bi88., bi89., bi89., bi8a., bi9.., bi91., bi92., bi93., bi94., bi94., bi95., bi95., bi96., bi96., bi97., bi98., bi99., bi9A., bi9A., bi9z., biA.., biA1., biA2., biA3., biA4., biB.., biB1., biB2., biB3., biBx., biBy., biBz., biC.., biC1., biC2., biC3., biC4., biC5., biC6., bk3.., bk31., bk32., bk33., bk34., bk37., bk38., bk3B., bk3C., bk3D., bk3E., bk3F., bk3G., bk3H., bk4.., bk41.,

Comorbidities	Read codes
	bk42., bk43., bk44., bk45., bk46., bk4A., bk4B., bk4C., bk4s., bk4t., bk4u., bk4v., bk4w., bk5.., bk51., bk52., bk53., bk54., bk55., bk56., bk7.., bk71., bk72., bk73., bk74., bk75., bk76., bk77., bk78., bk79., bk7z., bk8.., bk81., bk82., bk83., bk84., bk85., bk8z., bk9.., bk91., bk92., bk92., bk93., bk9x., bk9y., bk9z., bkB.., bkB1., bkB2., bkB3., bkB4., bkB5., bkB6., bkJ.., bkJ1., bkJ2., bkJ3., bkJ4., bkJ5., bkJ6., bd□c, bd1.., bd11., bd12., bd13., bd14., bd15., bd16., bd17., bd18., bd19., bd1A., bd1B., bd1C., bd1D., bd1E., bd1F., bd1G., bd1I., bd1J., bd1K., bd1L., bd1M., bd1N., bd1O., bd1P., bd1Q., bd1R., bd1S., bd1T., bd1U., bd1V., bd1W., bd1X., bd1Y., bd1Z., bd1a., bd1b., bd1c., bd1d., bd1e., bd1f., bd1g., bd1h., bd1i., bd1j., bd1k., bd1l., bd1l., bd1m., bd1n., bd1o., bd1p., bd1r., bd1r., bd1s., bd1t., bd1u., bd1v., bd1w., bd1x., bd1y., bd1z., bd2.., bd21., bd22., bd23., bd2w., bd2x., bd2y., bd3.., bd31., bd32., bd34., bd35., bd36., bd37., bd3a., bd3b., bd3c., bd3c., bd3d., bd3e., bd3f., bd3g., bd3h., bd3i., bd3j., bd3k., bd3l., bd3x., bd3z., bd4.., bd41., bd4z., bd5.., bd51., bd52., bd53., bd54., bd55., bd56., bd57., bd58., bd59., bd5a., bd5t., bd5u., bd5v., bd5w., bd5x., bd5y., bd6.., bd61., bd62., bd64., bd65., bd66., bd67., bd68., bd6b., bd6c., bd6d., bd6e., bd6w., bd6x., bd6z., bd7.., bd71., bd72., bd7y., bd7z., bd8.., bd81., bd82., bd83., bd84., bd85., bd86., bd87., bd88., bd89., bd8a., bd8b., bd8c., bd8d., bd8e., bd8f., bd8g., bd8h., bd8i., bd8k., bd8l., bd8m., bd8n., bd8o., bd8u., bd9.., bda.., bda1., bda2., bda3., bda4., bday., bdaz., bdb.., bdc.., bdc1., bdc1., bdc2., bdc3., bdc4., bdc5., bdcu., bdcv., bdcw., bdcx., bdd.., bdd1., bdd1., bdd2., bddz., bde.., bde1., bde2., bde3., bde4., bde5., bde6., bde7., bde8., bde9., bdeQ., bdeR., bdea., bdeb., bdec., bded., bdee., bdef., bdeg., bdeh., bdei., bdej., bdek., bdel., bdf.., bdf1., bdf2., bdf3., bdf4., bdf5., bdf5., bdf6., bdf6., bdf7., bdf8., bdf9., bdfA., bdfB., bdfC., bdfD., bdfE., bdfF., bdfG., bdfH., bdfI., bdfJ., bdfK., bdfL., bdfM., bdfw., bdfx., bdfy., bdfz., bdg.., bdg1., bdg2., bdh.., bdh1., bdh2., bdh3., bdh4., bdi.., bdi1., bdi2., bdj.., bdj1., bdj2., bdj3., bdj4., bdj5., bdl.., bdl1., bdl1., bdl2., bdl3., bdl4., bdl5., bdl6., bdl7., bdl8., bdl8., bdm.., bdm1., bdm2., bdmy., bdmz., bdn.., bdn1., bdn2., bdn3., bdn4., bdn5., bdn6., bb3.., bb31., bb32., bb33., bb34., bb35., bb36., bb37., bb38., bb39., bb3A., bb3B., bb3C., bb3D., bb3F., bb3G., bb3H., bb3J., bb3K., bb3L., bb3M., bb3N., bb3O., bb3P., bb3Q., bb3a., bb3b., bb3d., bb3e., bb3f., bb3g., bb3h., bb3i., bb3j., bb3k., bb3l., bb3m., bb3n., bb3p., bb3q., bb3r., bb3s., bb3v., bb3w., bb3x., bb3y., bb3z., bl5.., bl51., bl52., bl53., bl54., bl55., bl56., bl57., bl58., bl59., bl5A., bl5B., bl5C., bl5D., bl5E., bl5F., bl5G., bl5H., bl5I., bl5J., bl5K., bl5L., bl5M., bl5N., bl5O., bl5P., bl5Q., bl5R., bl5S., bl5T., bl5U., bl5V., bl5V., bl5W., bl5W., bl5X., bl5Y., bl5Z., bl5a., bl5b., bl5c., bl5d., bl5e., bl5f., bl5g., bl5h., bl5j., bl5k., bl5l., bl5m., bl5n., bl5o., bl5p., bl5q., bl5r., bl5s., bl5t., bl5u., bl5v., bl5w., bl5x., bl5y., bl5z., bl7.., bl71., bl72., bl73., bl74., bl7w., bl7x., bl7y., bl7z., bl8.., bl81., bl82., bl83., bl84., bl85., bl86., bl89., bl8A., bl8B., bl8C., bl8D., bl8E., bl8F., bl8G., bl8H., bl8J., bl8K., bl8L., bl8M., bl8O., bl8P., bl8Q., bl8R., bl8S., bl8T., bl8U., bl8V., bl8W., bl8X., bl8Y., bl8Z., bl8a., bl8b., bl8c., bl8d., bl8e., bl8f., bl8g., bl8h., bl8i., bl8j., bl8k., bl8l., bl8m., bl8n., bl8o., bl8p., bl8q., bl8r., bl8s., bl8t., bl8u., bl8v., bl8w., bl8x., bl8y., bl8z., bla.., bla.., bla1., bla1., bla2., bla2., blb.., blb1., blb2., blb3., blb4., blb5., blb5., blb6., blb6., blb7., blb8., blc.., blc1., blc2., blc3., blc4., blc5., blc6., blc7., blc8., blc9., blca., blcb., blcc., blcd., blce., blcf., blcg., blch., blci., blcj., blck., blcl., blcm., blcn., blco., blcp., blcq., blcr., blcs., blct., ble.., ble1., ble2., ble3., ble4., ble5., blg.., blg1., blg2., blg3., blg4., blg5., blg6., blh.., blh1., blh2., blh3., blh4., blj.., blj1., blj2., blj3., blj4., blj5., blj6., blj7., blj8., blj9., bljA., bljB., bljC., bljD., bljE., bljF., bljG., bljH., bljJ., bljK., bljL., bljM., bljN., bljO., bljP., bljQ., bljR., bljS., bljT., bljU., bljV., bljW., bljX., bljY., bljZ., blja., bljb., bljc., bljd., blje., bljf., bll.., bll1., bll2., bll3., bll4., bll5., bll6., bll7., bll8., bll9., blla., bllb., bllc., blld., blle., bllf., bllg., bllh., blli., bllj., bllk., blll., dt1.., dt13., dt14., b2..., b21.., b211., b212., b213., b214., b215., b216., b217., b218., b219., b21A., b21B., b21a., b21b., b22.., b221., b222., b22y., b22z., b23.., b231., b232., b23y., b23z.,

Comorbidities	Read codes
	b24.., b25.., b251., b25z., b26.., b261., b262., b263., b264., b26y., b26z., b27.., b271., b27z., b28.., b281., b282., b283., b284., b285., b286., b287., b288., b289., b28z., b29.., b291., b29z., b2a.., b2a1., b2az., b2b.., b2b1., b2b2., b2b3., b2bz., b2c.., b2c1., b2cz., b2d.., b2d1., b2dz., bA1.., bA11., bA12., bA1y., bA1z., bi1D., bi1E., bi1F., bi1G., bi1e., bi1f., bi1s., bi28., bi2b., bi3n., bi3p., bi3s., bi3t., bi3u., bi3v., bi3w., bi3x., bi48., bi4F., bi55., bi56., biC7., biC8., bk35., bk36., bk39., bk3A., bk3y., bk3z., bk47., bk48., bk49., bk4x., bk4y., bk4z., bk57., bk58., bk59., bk5x., bk5y., bk5z., bk86., bk87., bk88., bk8w., bk8x., bk8y., bkC.., bkC1., bkC2., bkC3., bkCx., bkCy., bkCz., bkH.., bkH1., bkH2., bkH3., bkHx., bkHy., bkHz., bkI.., bkI1., bkI2., bkI3., bkI4., bkI5., bkL.., bkL1., bkL2., bkL3., bkL4., bkL5., bkL6., bd38., bd39., bdeA., bdeB., bdeC., bdeD., bdeE., bdeF., bdeG., bdeH., bdeJ., bdeK., bdeL., bdeM., bdeN., bdeO., bdeP., bdem., bden., bdeo., bdep., bdeq., bder., bdes., bdes., bdet., bdeu., bdev., bdew., bdex., bdey., bdez., bl5i., bh4.., bh5y., bh56., bh41., bh4x., bh5z., bh63., bh55., bh54., bh6B., bh1y., bh65., bh4z., bh14., bh4D., bh6A., bh68., bh4v., bh4B., bh6F., bh61., bh69., bh46., bh21., bh6E., bh4y., bh45., bh47., bh6H., bh42., bh6y., bh1z., bh5.., bh6C., bh6D., bh57., bh4C., bh4A., bh6G., bh66., bh1.., bh4w., bh53., bh44., bh52., bh43., bh5x., bh6z., bh13., bh51., bh64., bh67., bh49., bh48., bh11., bh2y., bh6.., bh12., bh62., bf39., bf26., bf1w., bf1x., bf35., bf3b., bf42., bf44., bf3a., bf4.., bf2.., bf2d., bf22., bf2v., bf13., bf2c., bf27., bf2j., bf23., bf24., bf12., bf3d., bf25., bf3c., bf31., bf43., bf36., bf2e., bf33., bf2b., bf2g., bf21., bf11., bf2h., bf2z., bf32., bf34., bf41., bf29., bf2a., bf2f., bf45., bf37., bf38., bf46., be3x., be2y., be3.., be3z., be3y., be1.., be2x., be22., be32., be21., be31., be2.., be33.,

Supplemental Table 3-1. Standardised incidence rate of liver disease by data sources (2004 to 2022)

Year	PEDW only group		WLGP only group		ADDE only group		Two or more data sources	
	STD incidence	STD 95% CI	STD incidence	STD 95% CI	STD incidence	STD 95% CI	STD incidence	STD 95% CI
**2004**	47.7	(45.1 to 50.4)	14.5	(13.1 to 16.0)	4.6	(3.8 to 5.6)	43.5	(41.1 to 46.1)
**2005**	56.2	(53.4 to 59.1)	16.2	(14.8 to 17.8)	4.4	(3.6 to 5.3)	44.7	(42.2 to 47.2)
**2006**	60.6	(57.7 to 63.6)	17.1	(15.6 to 18.6)	4.3	(3.5 to 5.1)	47.9	(45.4 to 50.5)
**2007**	60.0	(57.1 to 63.0)	14.4	(13.1 to 15.9)	4.9	(4.1 to 5.8)	44.4	(42.0 to 47.0)
**2008**	63.4	(60.5 to 66.5)	18.6	(17.1 to 20.3)	6.2	(5.3 to 7.3)	45.9	(43.4 to 48.5)
**2009**	67.3	(64.3 to 70.4)	18.7	(17.1 to 20.3)	5.9	(5.0 to 6.9)	45.7	(43.2 to 48.2)
**2010**	70.2	(67.2 to 73.4)	21.9	(20.2 to 23.6)	5.8	(5.0 to 6.8)	47.2	(44.7 to 49.7)
**2011**	76.3	(73.1 to 79.5)	23.6	(21.9 to 25.4)	5.3	(4.5 to 6.2)	48.0	(45.5 to 50.6)
**2012**	77.6	(74.4 to 80.8)	21.6	(19.9 to 23.3)	5.6	(4.7 to 6.5)	47.9	(45.5 to 50.5)
**2013**	82.7	(79.4 to 86.0)	22.3	(20.6 to 24.1)	6.0	(5.1 to 6.9)	47.1	(44.6 to 49.6)
**2014**	88.8	(85.5 to 92.3)	26.1	(24.3 to 28.0)	6.0	(5.2 to 7.0)	49.2	(46.7 to 51.7)
**2015**	95.6	(92.1 to 99.1)	31.6	(29.6 to 33.7)	6.0	(5.1 to 6.9)	53.1	(50.5 to 55.7)
**2016**	109.5	(105.8 to 113.3)	44.1	(41.8 to 46.6)	6.0	(5.1 to 6.9)	55.5	(52.9 to 58.3)
**2017**	113.9	(110.1 to 117.8)	47.1	(44.7 to 49.6)	5.8	(5.0 to 6.7)	60.6	(57.9 to 63.5)
**2018**	128.2	(124.2 to 132.3)	59.6	(56.8 to 62.4)	6.4	(5.5 to 7.4)	63.0	(60.2 to 65.9)
**2019**	140.5	(136.4 to 144.8)	67.3	(64.4 to 70.2)	5.5	(4.7 to 6.4)	59.5	(56.8 to 62.3)
**2020**	133.6	(129.5 to 137.7)	46.9	(44.5 to 49.4)	7.5	(6.6 to 8.5)	45.6	(43.3 to 48.1)
**2021**	168.4	(163.9 to 173)	67.6	(64.7 to 70.6)	6.3	(5.5 to 7.3)	46.2	(43.8 to 48.6)
**2022**	151.8	(147.6 to 156.2)	80.2	(77.0 to 83.4)	6.4	(5.5 to 7.3)	31.1	(29.2 to 33.1)

Abbreviation: STD: standardized; CI: confidence interval

Supplemental Table 3-2 Standardised incidence rate of liver disease by disease stages (2004 to 2022)

Year	Stage 1		Stage 2		Stage 3		Stage 4		Stage 5	
	STD incidence	STD 95% CI	STD incidence	STD 95% CI	STD incidence	STD 95% CI	STD incidence	STD 95% CI	STD incidence	STD 95% CI
**2004**	78.7	(75.4 to 82.1)	6.8	(5.8 to 7.8)	5.9	(5.0 to 6.9)	5.8	(4.9 to 6.8)	3.3	(2.6 to 4.0)
**2005**	87.5	(84.0 to 91.0)	7.8	(6.7 to 8.9)	5.8	(5.0 to 6.8)	6.4	(5.4 to 7.5)	3.6	(2.9 to 4.5)
**2006**	94.1	(90.5 to 97.8)	7.9	(6.9 to 9.0)	6.9	(6.0 to 8.0)	7.1	(6.1 to 8.1)	4.3	(3.5 to 5.1)
**2007**	89.5	(86.0 to 93.1)	8.2	(7.1 to 9.3)	6.3	(5.4 to 7.3)	7.0	(6.1 to 8.1)	4.2	(3.5 to 5.0)
**2008**	97.2	(93.6 to 100.9)	8.9	(7.8 to 10.1)	8.9	(7.8 to 10.1)	6.3	(5.4 to 7.3)	5.0	(4.2 to 5.9)
**2009**	101.1	(97.5 to 104.9)	9.3	(8.2 to 10.4)	8.0	(7.0 to 9.1)	6.8	(5.9 to 7.8)	4.7	(3.9 to 5.7)
**2010**	108.9	(105.1 to 112.8)	8.5	(7.4 to 9.6)	8.6	(7.6 to 9.7)	6.9	(5.9 to 7.9)	3.9	(3.2 to 4.7)
**2011**	113.6	(109.7 to 117.5)	10.4	(9.3 to 11.7)	8.7	(7.7 to 9.9)	6.8	(5.8 to 7.8)	4.9	(4.1 to 5.9)
**2012**	113.5	(109.7 to 117.5)	9.6	(8.5 to 10.8)	8.3	(7.3 to 9.4)	6.7	(5.8 to 7.7)	4.9	(4.1 to 5.8)
**2013**	115.4	(111.6 to 119.4)	10.3	(9.1 to 11.5)	8.9	(7.9 to 10.1)	7.4	(6.4 to 8.5)	6.0	(5.2 to 7.0)
**2014**	126.4	(122.4 to 130.5)	11.4	(10.2 to 12.6)	9.3	(8.2 to 10.4)	7.0	(6.1 to 8.0)	5.1	(4.3 to 6.0)
**2015**	137.9	(133.7 to 142.2)	13.5	(12.2 to 14.9)	12.0	(10.8 to 13.3)	6.2	(5.4 to 7.2)	5.9	(5.0 to 6.8)
**2016**	163.2	(158.7 to 167.9)	15.2	(13.8 to 16.7)	12.5	(11.3 to 13.8)	7.6	(6.7 to 8.7)	5.8	(5.0 to 6.7)
**2017**	174.6	(169.9 to 179.4)	16.3	(14.9 to 17.8)	12.5	(11.3 to 13.8)	7.2	(6.3 to 8.2)	5.8	(5.0 to 6.7)
**2018**	197.7	(192.7 to 202.7)	19.6	(18.1 to 21.2)	13.3	(12.0 to 14.6)	8.2	(7.2 to 9.2)	6.1	(5.2 to 7.0)
**2019**	212.1	(207.0 to 217.4)	19.3	(17.8 to 20.9)	15.5	(14.1 to 16.9)	7.7	(6.7 to 8.7)	5.5	(4.8 to 6.4)
**2020**	180.4	(175.7 to 185.3)	16.6	(15.2 to 18.1)	12.3	(11.1 to 13.6)	7.6	(6.6 to 8.6)	6.1	(5.2 to 7.0)
**2021**	231.0	(225.6 to 236.4)	18.2	(16.8 to 19.8)	15.4	(14.1 to 16.9)	6.9	(6.1 to 7.9)	6.0	(5.2 to 7.0)
**2022**	211.4	(206.3 to 216.5)	19.0	(17.5 to 20.6)	16.4	(15.1 to 17.9)	6.1	(5.3 to 7.0)	6.5	(5.7 to 7.5)

Abbreviation: STD: standardized; CI: confidence interval

Supplemental Table 3-3, Standardised incidence rate of liver disease by aetiologies (2004 to 2022)

Year	ArLD	NAFLD	Metabolic liver disease	HBV	HCV	Autoimmune liver disease	Haemochromatosis	ArLD overlap	Non-ArLD overlap	Hepatitis not specified	Congestive hepatopathy	Toxic liver disease	Miscellaneous
	STD incidence (95% CI)	STD incidence (95% CI)	STD incidence (95% CI)	STD incidence (95% CI)	STD incidence (95% CI)	STD incidence (95% CI)	STD incidence (95% CI)	STD incidence (95% CI)	STD incidence (95% CI)	STD incidence (95% CI)	STD incidence (95% CI)	STD incidence (95% CI)	STD incidence (95% CI)
2004	23.4 (21.7 to 25.3)	11.8 (10.6 to 13.2)	5.1 (4.3 to 6.1)	0.7 (0.4 to 1.0)	3.1 (2.5 to 3.9)	16.9 (15.4 to 18.6)	3.5 (2.8 to 4.2)	0.3 (0.2 to 0.6)	0.4 (0.2 to 0.8)	4.3 (3.5 to 5.2)	0.8 (0.5 to 1.3)	1.9 (1.4 to 2.5)	6.4 (5.5 to 7.4)
2005	22.8 (21.0 to 24.6)	15.2 (13.8 to 16.7)	5.4 (4.6 to 6.4)	0.6 (0.4 to 1.0)	2.9 (2.3 to 3.6)	21 (19.3 to 22.8)	4.3 (3.6 to 5.2)	0.4 (0.2 to 0.7)	0.4 (0.2 to 0.7)	4.6 (3.8 to 5.4)	0.6 (0.3 to 1.0)	2.5 (2.0 to 3.1)	6.8 (5.9 to 7.9)
2006	22.7 (21.0 to 24.5)	19 (17.4 to 20.6)	5.8 (4.9 to 6.8)	0.7 (0.5 to 1.1)	3.9 (3.2 to 4.7)	20 (18.3 to 21.8)	5.5 (4.7 to 6.5)	--	0.8 (0.5 to 1.2)	5.4 (4.6 to 6.4)	0.7 (0.4 to 1.1)	2 (1.5 to 2.6)	7.3 (6.3 to 8.4)
2007	22.7 (21.0 to 24.5)	18.1 (16.6 to 19.7)	5.7 (4.8 to 6.7)	0.6 (0.4 to 1.0)	2.8 (2.2 to 3.5)	18.8 (17.2 to 20.5)	4.5 (3.8 to 5.4)	--	0.8 (0.5 to 1.2)	5.4 (4.5 to 6.3)	0.7 (0.4 to 1.0)	1.7 (1.2 to 2.2)	7.5 (6.4 to 8.6)
2008	20.7 (19.0 to 22.4)	21.9 (20.3 to 23.7)	5.2 (4.4 to 6.2)	0.8 (0.5 to 1.2)	3.7 (3.1 to 4.5)	21.5 (19.8 to 23.3)	5.1 (4.3 to 6.0)	--	0.7 (0.4 to 1.1)	6.8 (5.8 to 7.8)	0.9 (0.6 to 1.3)	1.3 (0.9 to 1.7)	8.2 (7.2 to 9.4)
2009	21.4 (19.7 to 23.1)	23.7 (22.0 to 25.5)	5.7 (4.9 to 6.7)	0.7 (0.4 to 1.1)	3.1 (2.5 to 3.8)	25 (23.2 to 26.9)	5.8 (5.0 to 6.8)	0.5 (0.3 to 0.8)	0.6 (0.3 to 0.9)	4.9 (4.1 to 5.8)	0.6 (0.4 to 1.0)	1.0 (0.7 to 1.4)	8.2 (7.2 to 9.3)
2010	21.1 (19.5 to 22.8)	29.4 (27.4 to 31.4)	5.5 (4.6 to 6.4)	0.8 (0.5 to 1.1)	3.4 (2.7 to 4.1)	26.9 (25 to 28.9)	5.8 (4.9 to 6.7)	0.4 (0.2 to 0.6)	0.5 (0.2 to 0.8)	5.5 (4.7 to 6.5)	0.7 (0.4 to 1.1)	0.9 (0.6 to 1.3)	8.2 (7.2 to 9.4)
2011	18.8 (17.3 to 20.4)	32.9 (30.9 to 35.0)	6.6 (5.7 to 7.6)	1.3 (0.9 to 1.7)	3.6 (2.9 to 4.3)	25.1 (23.3 to 27.0)	5.9 (5.1 to 6.9)	0.4 (0.2 to 0.7)	0.6 (0.4 to 1.0)	6.1 (5.2 to 7.1)	0.4 (0.2 to 0.8)	1.5 (1.1 to 2.0)	10.4 (9.2 to 11.6)
2012	17.4 (15.9 to 18.9)	32.8 (30.8 to 35.0)	8.3 (7.3 to 9.4)	0.8 (0.5 to 1.2)	4.2 (3.5 to 5.0)	23.4 (21.7 to 25.3)	6.4 (5.5 to 7.4)	0.4 (0.2 to 0.7)	0.6 (0.4 to 1.0)	6.3 (5.4 to 7.3)	0.9 (0.6 to 1.3)	1.4 (1.0 to 1.9)	10.6 (9.5 to 11.9)
2013	17.8 (16.3 to 19.4)	36 (33.9 to 38.2)	8.6 (7.5 to 9.7)	1 (0.7 to 1.5)	4.7 (4 to 5.6)	18.4 (16.9 to 20.0)	5.8 (5.0 to 6.7)	0.4 (0.2 to 0.7)	0.6 (0.4 to 1.0)	8.3 (7.3 to 9.4)	0.8 (0.5 to 1.2)	1.1 (0.7 to 1.5)	11.9 (10.7 to 13.2)
2014	17.2 (15.8 to 18.8)	44.9 (42.5 to 47.4)	8.2 (7.2 to 9.3)	0.7 (0.4 to 1.1)	5.7 (4.9 to 6.7)	17.4 (15.9 to 18.9)	6.4 (5.5 to 7.4)	0.3 (0.2 to 0.6)	0.8 (0.5 to 1.1)	7.7 (6.7 to 8.7)	0.8 (0.6 to 1.2)	0.9 (0.6 to 1.4)	15.3 (13.9 to 16.7)
2015	17.6 (16.2	53.6 (51.0	8.6 (7.6 to	0.9 (0.6 to	5.3 (4.5 to	18.9 (17.3 to	5.8 (5.0 to 6.8)	0.4 (0.2 to	0.5 (0.3 to	8.0 (7.0 to	0.9 (0.6 to	1.1 (0.7 to	16.2 (14.8 to

	to 19.2)	to 56.3)	9.7)	1.4)	6.3)	20.5)		0.8)	0.8)	9.1)	1.3)	1.5)	17.7)
**2016**	18.3 (16.8 to 19.9)	67.6 (64.7 to 70.6)	11.6 (10.4 to 12.9)	1.9 (1.4 to 2.5)	7.0 (6.1 to 8.0)	18.4 (16.9 to 20.0)	7.3 (6.3 to 8.3)	0.6 (0.3 to 0.9)	1.0 (0.7 to 1.5)	9.4 (8.3 to 10.6)	1.1 (0.7 to 1.5)	1.1 (0.8 to 1.6)	18.0 (16.5 to 19.6)
**2017**	19.6 (18 to 21.2)	77.3 (74.2 to 80.5)	10.5 (9.4 to 11.8)	2.5 (2.0 to 3.2)	7.2 (6.2 to 8.3)	16.7 (15.3 to 18.2)	7.3 (6.3 to 8.3)	0.9 (0.6 to 1.3)	0.9 (0.6 to 1.3)	10.2 (9.1 to 11.4)	1.1 (0.8 to 1.6)	1.2 (0.8 to 1.6)	19.2 (17.6 to 20.8)
**2018**	19.2 (17.6 to 20.8)	94.9 (91.5 to 98.5)	11.2 (10.0 to 12.4)	1.9 (1.5 to 2.5)	6.0 (5.2 to 7.0)	18.0 (16.5 to 19.5)	7.9 (7.0 to 9.0)	0.8 (0.5 to 1.2)	1.0 (0.6 to 1.4)	11.0 (9.9 to 12.3)	1.2 (0.8 to 1.6)	1.5 (1.1 to 2.1)	23.0 (21.4 to 24.8)
**2019**	20.8 (19.2 to 22.5)	107.2 (103.6 to 111.0)	11.2 (10.0 to 12.4)	2.5 (1.9 to 3.1)	5.7 (4.9 to 6.7)	17.3 (15.9 to 18.8)	8.2 (7.2 to 9.3)	0.7 (0.4 to 1.1)	0.8 (0.5 to 1.2)	11.3 (10.2 to 12.6)	1.3 (0.9 to 1.7)	1.1 (0.8 to 1.6)	24.0 (22.3 to 25.8)
**2020**	18.5 (17 to 20.1)	83 (79.8 to 86.3)	9.8 (8.7 to 11.0)	1.5 (1.1 to 2.1)	5.1 (4.3 to 6.1)	13.6 (12.4 to 15)	7.1 (6.2 to 8.1)	0.6 (0.3 to 0.9)	0.8 (0.5 to 1.2)	11.1 (10.0 to 12.4)	1.4 (1.0 to 1.8)	1.6 (1.2 to 2.1)	26.2 (24.5 to 28.0)
**2021**	20.3 (18.8 to 22.0)	117.6 (113.8 to 121.5)	11.8 (10.6 to 13.1)	1.7 (1.2 to 2.2)	3.6 (3.0 to 4.4)	15.7 (14.3 to 17.1)	7.6 (6.6 to 8.6)	0.4 (0.2 to 0.7)	1.3 (0.9 to 1.8)	13.5 (12.3 to 14.9)	1.1 (0.7 to 1.5)	1.3 (0.9 to 1.8)	35.1 (33.0 to 37.2)
**2022**	20.7 (19.1 to 22.4)	111.2 (107.5 to 115.0)	12.0 (10.8 to 13.2)	1.2 (0.8 to 1.6)	2.2 (1.7 to 2.9)	13.8 (12.6 to 15.2)	8.1 (7.1 to 9.2)	0.4 (0.2 to 0.6)	1.2 (0.8 to 1.7)	11.8 (10.7 to 13.1)	1.3 (0.9 to 1.8)	1.0 (0.7 to 1.5)	26.4 (24.7 to 28.2)

Abbreviation: ArLD: alcohol-related liver disease; NAFLD: non-alcohol fatty liver disease; HBV: hepatitis B virus; HCV: hepatitis C virus; STD: standardised; CI: confidence interval

Supplemental Table 4-1, Standardised incidence rate of NAFLD by data sources (2004 to 2022)

Year	PEDW-only		WLGP-only		ADDE-only		Two or more data sources	
	STD incidence	STD 95% CI	STD incidence	STD 95% CI	STD incidence	STD 95% CI	STD incidence	STD 95% CI
**2004**	3.8	(3.2 to 4.6)	4.6	(3.8 to 5.5)	0.5	(0.3 to 0.9)	2.9	(2.3 to 3.6)
**2005**	5.0	(4.2 to 5.9)	5.6	(4.8 to 6.6)	0.4	(0.2 to 0.7)	4.2	(3.4 to 5.0)
**2006**	6.4	(5.5 to 7.4)	7.7	(6.7 to 8.8)	--	--	4.6	(3.9 to 5.5)
**2007**	6.6	(5.7 to 7.6)	6.9	(6.0 to 8.0)	0.4	(0.2 to 0.7)	4.2	(3.5 to 5.0)
**2008**	6.2	(5.4 to 7.2)	10.6	(9.5 to 11.9)	0.7	(0.4 to 1.0)	4.4	(3.7 to 5.2)
**2009**	6.8	(5.8 to 7.8)	10.8	(9.6 to 12.0)	0.6	(0.3 to 0.9)	5.6	(4.8 to 6.5)
**2010**	7.2	(6.2 to 8.2)	14.9	(13.5 to 16.4)	0.7	(0.4 to 1.0)	6.6	(5.7 to 7.6)
**2011**	10.0	(8.9 to 11.2)	15.4	(14.0 to 16.9)	0.4	(0.2 to 0.6)	7.1	(6.2 to 8.1)
**2012**	11.7	(10.5 to 13.0)	13.8	(12.5 to 15.2)	0.4	(0.2 to 0.7)	6.9	(6.0 to 7.9)
**2013**	13.0	(11.8 to 14.4)	15.2	(13.8 to 16.7)	1.0	(0.7 to 1.4)	6.7	(5.8 to 7.7)
**2014**	17.5	(16.0 to 19.0)	18.2	(16.7 to 19.8)	1.1	(0.7 to 1.5)	8.2	(7.2 to 9.3)
**2015**	20.6	(19.0 to 22.3)	22.9	(21.2 to 24.7)	1.0	(0.7 to 1.4)	9.1	(8.1 to 10.3)
**2016**	23.3	(21.6 to 25.1)	31.8	(29.9 to 33.9)	1.1	(0.8 to 1.6)	11.3	(10.1 to 12.5)
**2017**	28.1	(26.3 to 30.1)	34.4	(32.4 to 36.6)	1.2	(0.8 to 1.6)	13.5	(12.3 to 14.9)
**2018**	36.5	(34.4 to 38.7)	43.1	(40.8 to 45.6)	1.5	(1.1 to 2.0)	13.8	(12.5 to 15.1)
**2019**	41.7	(39.4 to 44.0)	50.5	(48.0 to 53.1)	1.4	(1.0 to 1.9)	13.6	(12.4 to 15.0)
**2020**	40.8	(38.6 to 43.2)	32.0	(30.0 to 34.1)	1.8	(1.3 to 2.3)	8.4	(7.4 to 9.5)
**2021**	58.2	(55.6 to 61.0)	49.0	(46.5 to 51.5)	1.4	(1.0 to 1.9)	9.0	(8.0 to 10.1)
**2022**	52.9	(50.4 to 55.5)	53.6	(51.0 to 56.2)	1.3	(0.9 to 1.8)	3.4	(2.7 to 4.0)

Abbreviation: STD: standardized; CI: confidence interval

Supplemental Table 4-2, Standardised incidence rate of ArLD by data sources (2004 to 2022)

Year	PEDW-only		WLGP-only		ADDE-only		Two or more data sources	
	STD incidence	STD 95% CI	STD incidence	STD 95% CI	STD incidence	STD 95% CI	STD incidence	STD 95% CI
**2004**	4.7	(4.0 to 5.6)	3.2	(2.5 to 3.9)	--	--	15.2	(13.8 to 16.7)
**2005**	5.3	(4.5 to 6.2)	2.8	(2.3 to 3.5)	0.6	(0.3 to 1.0)	14.0	(12.6 to 15.5)
**2006**	5.3	(4.5 to 6.2)	2.8	(2.2 to 3.4)	0.4	(0.2 to 0.7)	14.3	(12.9 to 15.7)
**2007**	5.6	(4.7 to 6.5)	2.3	(1.8 to 2.9)	0.6	(0.4 to 1.0)	14.2	(12.9 to 15.7)
**2008**	4.9	(4.1 to 5.8)	2.1	(1.6 to 2.7)	0.8	(0.5 to 1.2)	12.9	(11.7 to 14.3)
**2009**	5.8	(4.9 to 6.7)	2.1	(1.6 to 2.7)	0.9	(0.6 to 1.4)	12.6	(11.3 to 13.9)
**2010**	5.2	(4.4 to 6.1)	2.5	(1.9 to 3.1)	0.9	(0.6 to 1.3)	12.6	(11.3 to 13.9)
**2011**	4.5	(3.8 to 5.3)	2.5	(1.9 to 3.1)	0.7	(0.4 to 1.1)	11.1	(10.0 to 12.4)
**2012**	4.7	(4.0 to 5.6)	1.8	(1.4 to 2.4)	0.7	(0.4 to 1.1)	10.1	(9.0 to 11.3)
**2013**	4.8	(4.0 to 5.6)	2.0	(1.5 to 2.6)	0.6	(0.4 to 1.0)	10.4	(9.2 to 11.6)
**2014**	4.6	(3.9 to 5.5)	2.2	(1.7 to 2.8)	0.6	(0.4 to 1.0)	9.8	(8.7 to 11.0)
**2015**	4.4	(3.6 to 5.2)	2.3	(1.8 to 2.9)	0.7	(0.4 to 1.0)	10.3	(9.2 to 11.5)
**2016**	4.5	(3.8 to 5.4)	3.8	(3.2 to 4.6)	0.8	(0.6 to 1.2)	9.0	(8.0 to 10.2)
**2017**	4.1	(3.4 to 4.9)	4.4	(3.7 to 5.2)	0.6	(0.4 to 0.9)	10.5	(9.4 to 11.8)
**2018**	4.0	(3.3 to 4.8)	5.0	(4.2 to 5.9)	0.7	(0.4 to 1)	9.5	(8.4 to 10.6)
**2019**	4.7	(3.9 to 5.5)	5.4	(4.6 to 6.3)	0.9	(0.6 to 1.3)	9.8	(8.7 to 11.0)
**2020**	5.0	(4.2 to 5.9)	4.8	(4.1 to 5.7)	1.0	(0.7 to 1.4)	7.7	(6.7 to 8.7)
**2021**	4.4	(3.7 to 5.2)	7.0	(6.1 to 8.0)	1.2	(0.8 to 1.6)	7.8	(6.8 to 8.9)
**2022**	5.6	(4.8 to 6.6)	8.9	(7.9 to 10.1)	1.0	(0.7 to 1.4)	5.2	(4.4 to 6.1)

Abbreviation: STD: standardized; CI: confidence interval

Supplemental Table 5-1 Comorbidities associated with liver disease by stages (2004 to 2022)

**Comorbidities**	**Stage 1, N = 94,529**[1]	**Stage 2, N = 4,562**[1]	**Stage 3, N = 3,040**[1]	**Stage 4, N = 3,221**[1]	**Stage 5, N = 2,233**[1]
**CVD related conditions**	7,018(7.4%)	587(12.9%)	310(10.2%)	457(14.2%)	264(11.8%)
**Diabetes**	6,135(6.5%)	612(13.4%)	303(10.0%)	234(7.3%)	200(9.0%)
**Hypertension/anti-hypertensive**	34,022(36.0%)	1,939(42.5%)	1,165(38.3%)	1,232(38.2%)	1,006(45.1%)

[1] **n(%)**

Supplemental Table 5-2 Comorbidities associated with liver disease by aetiologies (2004-2022)

**Comorbidities**	**ArLD, N = 19,760**[1]	**NAFLD, N = 33,655**[1]	**Metablic liver disease, N = 5,469**[1]	**HBV, N = 1,063**[1]	**HCV, N = 3,539**[1]	**Autoimmune liver disease, N = 13,582**[1]	**Haemochromatosis, N = 4,111**[1]	**Hepatitis not specified, N = 4,783**[1]	**Congestive hepatopathy, N = 574**[1]	**Toxic liver disease, N = 757**[1]	**Miscellaneous, N = 8,426**[1]
**CVD related conditions**	1,442(7.3%)	1,837(5.5%)	578(10.9%)	54(5.2%)	177(5.5%)	1,159(8.6%)	227(5.6%)	466(9.7%)	135(23.6%)	58(7.7%)	885(10.5%)
**Diabetes**	1,185(6.0%)	2,776(8.4%)	380(7.2%)	46(4.4%)	132(4.1%)	752(5.6%)	175(4.3%)	368(7.7%)	42(7.3%)	26(3.4%)	253(3.0%)
**Hypertension/antihypertensives**	6,911(35.0%)	12,455(37.5%)	1,916(36.1%)	197(19.1%)	632(19.5%)	4,670(34.8%)	1,422(35.2%)	1,875(39.2%)	273(47.6%)	203(26.9%)	3,468(41.2%)

[1] n(%)
